# Genome-wide analysis of 53,400 people with irritable bowel syndrome highlights shared genetic pathways with mood and anxiety disorders

**DOI:** 10.1038/s41588-021-00950-8

**Published:** 2021-11-05

**Authors:** Chris Eijsbouts, Tenghao Zheng, Nicholas A. Kennedy, Ferdinando Bonfiglio, Carl A. Anderson, Loukas Moutsianas, Joanne Holliday, Jingchunzi Shi, Suyash Shringarpure, 23andMe Research Team, Michelle Agee, Stella Aslibekyan, Adam Auton, Robert K. Bell, Katarzyna Bryc, Sarah K. Clark, Sarah L. Elson, Kipper Fletez-Brant, Pierre Fontanillas, Nicholas A. Furlotte, Pooja M. Gandhi, Karl Heilbron, Barry Hicks, David A. Hinds, Karen E. Huber, Ethan M. Jewett, Yunxuan Jiang, Aaron Kleinman, Keng-Han Lin, Nadia K. Litterman, Marie K. Luff, Jey C. McCreight, Matthew H. McIntyre, Kimberly F. McManus, Joanna L. Mountain, Sahar V. Mozaffari, Priyanka Nandakumar, Elizabeth S. Noblin, Carrie A. M. Northover, Jared O’Connell, Aaron A. Petrakovitz, Steven J. Pitts, G. David Poznik, J. Fah Sathirapongsasuti, Anjali J. Shastri, Janie F. Shelton, Chao Tian, Joyce Y. Tung, Robert J. Tunney, Vladimir Vacic, Xin Wang, Amir S. Zare, Alexandru-Ioan Voda, The Bellygenes Initiative, Purna Kashyap, Lin Chang, Emeran Mayer, Margaret Heitkemper, Gregory S. Sayuk, Tamar Ringel-Kulka, Yehuda Ringel, William D. Chey, Shanti Eswaran, Juanita L. Merchant, Robert J. Shulman, Luis Bujanda, Koldo Garcia-Etxebarria, Aldona Dlugosz, Greger Lindberg, Peter T. Schmidt, Pontus Karling, Bodil Ohlsson, Susanna Walter, Åshild O. Faresjö, Magnus Simren, Jonas Halfvarson, Piero Portincasa, Giovanni Barbara, Paolo Usai-Satta, Matteo Neri, Gerardo Nardone, Rosario Cuomo, Francesca Galeazzi, Massimo Bellini, Anna Latiano, Lesley Houghton, Daisy Jonkers, Alexander Kurilshikov, Rinse K. Weersma, Mihai Netea, Jonas Tesarz, Annika Gauss, Miriam Goebel-Stengel, Viola Andresen, Thomas Frieling, Christian Pehl, Rainer Schaefert, Beate Niesler, Wolfgang Lieb, Kurt Hanevik, Nina Langeland, Knut-Arne Wensaas, Sverre Litleskare, Maiken E. Gabrielsen, Laurent Thomas, Vincent Thijs, Robin Lemmens, Lukas Van Oudenhove, Mira Wouters, Gianrico Farrugia, Andre Franke, Matthias Hübenthal, Gonçalo Abecasis, Matthew Zawistowski, Anne Heidi Skogholt, Eivind Ness-Jensen, Kristian Hveem, Tõnu Esko, Maris Teder-Laving, Alexandra Zhernakova, Michael Camilleri, Guy Boeckxstaens, Peter J. Whorwell, Robin Spiller, Gil McVean, Mauro D’Amato, Luke Jostins, Miles Parkes

**Affiliations:** 1 Big Data Institute, Li Ka Shing Centre for Health Information and Discovery, University of Oxford, Oxford, UK; 2 Wellcome Centre for Human Genetics, University of Oxford, Oxford, UK; 3 Center for Molecular Medicine & Clinical Epidemiology Unit, Department of Medicine Solna, Karolinska Institutet, Stockholm, Sweden; 4 School of Biological Sciences, Monash University, Clayton, Victoria Australia; 5 IBD Pharmacogenetics, College of Medicine and Health, University of Exeter, Exeter, UK; 6 Wellcome Sanger Institute, Wellcome Genome Campus, Hinxton, UK; 7 William Harvey Research Institute, Barts & The London School of Medicine & Dentistry, Queen Mary University of London, London, UK; 8 Nuffield Department of Population Health, University of Oxford, Oxford, UK; 9 23andMe, Inc., Sunnyvale, CA USA; 10 Kennedy Institute of Rheumatology, University of Oxford, Oxford, UK; 11 Saint Edmund Hall, University of Oxford, Oxford, UK; 12 Enteric NeuroScience Program, Division of Gastroenterology and Hepatology, Department of Medicine, Mayo Clinic, Rochester, MN USA; 13 Institute of Clinical Molecular Biology, Christian-Albrechts-University of Kiel, Kiel, Germany; 14 Department of Dermatology, Quincke Research Center, University Hospital Schleswig-Holstein, Kiel, Germany; 15 Department of Biostatistics, University of Michigan, School of Public Health, Ann Arbor, MI USA; 16 Department of Laboratory Medicine, Children’s and Women’s Health, Norwegian University of Science and Technology, Trondheim, Norway; 17 Department of Public Health and Nursing, Norwegian University of Science and Technology, Trondheim, Norway; 18 Department of Medicine, Levanger Hospital, Nord-Trøndelag Hospital Trust, Levanger, Norway; 19 Department of Molecular Medicine and Surgery, Karolinska Institutet, Karolinska University Hospital, Stockholm, Sweden; 20 Estonian Genome Center, Institute of Genomics, University of Tartu, Tartu, Estonia; 21 Department of Genetics, University Medical Center Groningen, Groningen, the Netherlands; 22 Clinical Enteric Neuroscience Translational and Epidemiological Research and Division of Gastroenterology and Hepatology, Department of Medicine, Mayo Clinic, Rochester, MN USA; 23 David Geffen School of Medicine, University of California, Los Angeles, Los Angeles, CA USA; 24 Neurogastroenterology Unit, Wythenshawe Hospital, Centre for Gastrointestinal Sciences, University of Manchester, Manchester, UK; 25 Nottingham Digestive Diseases Centre, National Institute for Health Research Nottingham Biomedical Research Centre, Nottingham University Hospitals NHS Trust and the University of Nottingham, Nottingham, UK; 26 Biodonostia Health Research Institute, San Sebastian, Spain; 27 Gastrointestinal Genetics Lab, CIC bioGUNE – Basque Research and Technology Alliance, Derio, Spain; 28 IKERBASQUE, The Basque Science Foundation, Bilbao, Spain; 29 Christ Church, University of Oxford, Oxford, UK; 30 Division of Gastroenterology and Hepatology, Department of Medicine, University of Cambridge, Cambridge, UK; 31 Department of Gastroenterology, Cambridge University Hospital, Cambridge, UK; 32 Division of Digestive Diseases/Gastroenterology, David Geffen School of Medicine at University of California, Los Angeles, Los Angeles, CA USA; 33 Department of Biobehavioral Nursing and Health Informatics, University of Washington, Seattle, WA USA; 34 Division of Gastroenterology, Washington University School of Medicine, St. Louis, MO USA; 35 Gillings School of Global Public Health, University of North Carolina at Chapel Hill, Chapel Hill, NC USA; 36 Department of Medicine, Division of Gastroenterology and Hepatology, University of North Carolina at Chapel Hill, Chapel Hill, NC USA; 37 Division of Gastroenterology and Hepatology, Meir Medical Center, affiliated to Tel-Aviv University, Kfar Saba, Israel; 38 Division of Gastroenterology, Michigan Medicine, Ann Arbor, MI USA; 39 Division of Gastroenterology, University of Arizona College of Medicine, Tucson, AZ USA; 40 Children’s Nutrition Research Center, Baylor College of Medicine, Texas Children’s Hospital, Houston, TX USA; 41 Universidad del País Vasco, San Sebastian, Spain; 42 Department of Gastrointestinal and Liver Diseases, Biodonostia Health Research Institute, Sebastian, Spain; 43 Centro de Investigación Biomédica en Red de Enfermedades Hepáticas y Digestivas, Instituto Carlos III, Madrid, Spain; 44 Department of Medicine Solna, Karolinska Institutet, Center for Digestive Diseases, Karolinska University Hospital, Stockholm, Sweden; 45 Division of Medicine, Department of Public Health and Clinical Medicine, Umeå University, Umeå, Sweden; 46 Department of Internal Medicine, Lund University, Skåne University Hospital, Lund, Sweden; 47 Division of Neuro and Inflammation Science, Department of Clinical and Experimental Medicine, Linköping University, Linköping, Sweden; 48 Department of Health, Medicine and Caring Science/Society and Health/Public health, Linköpings University, Linköping, Sweden; 49 Department of Internal Medicine & Clinical Nutrition, Institute of Medicine, Sahlgrenska Academy, University of Gothenburg, Gothenburg, Sweden; 50 Department of Gastroenterology, School of Medical Sciences, Örebro University, Örebro, Sweden; 51 Department of Biomedical Sciences and Human Oncology, Clinica Medica ‘A. Murri’, University of Bari Medical School, Bari, Italy; 52 Department of Medical and Surgical Sciences, University of Bologna, St. Orsola – Malpighi Hospital, Bologna, Italy; 53 S.C. Gastroenterologia, Azienda Ospedaliera G. Brotzu, Cagliari, Italy; 54 Department of Medicine and Aging Sciences and CESI, G. D’Annunzio University & Foundation, Chieti, Italy; 55 Gastroenterology Unit, Department of Clinical Medicine and Surgery, University Federico II, Naples, Italy; 56 Gastroenterology Unit, Department of Medical Science, ‘Sant’Anna e San Sebastiano’ Hospital, Caserta, Italy; 57 Unità Operativa Complessa Gastroenterologia, Padova University Hospital, Padova, Italy; 58 Gastroenterology Unit, Department of Gastroenterology, University of Pisa, Pisa, Italy; 59 Division of Gastroenterology, Istituto di Ricovero e Cura a Carattere Scientifico ‘Casa Sollievo della Sofferenza’ Hospital, San Giovanni Rotondo, Italy; 60 Division of Gastroenterology and Surgical Sciences, Leeds Institute of Medical Research at St. James’s, University of Leeds, Leeds, UK; 61 Department of Gastroenterology, Mayo Clinic, Jacksonville, FL USA; 62 GI Sciences, Division of Diabetes, Endocrinology & Gastroenterology, University of Manchester, Manchester, UK; 63 Department of Internal Medicine, School for Nutrition and Translational Research in Metabolism, Maastricht University Medical Center+, Maastricht, the Netherlands; 64 Department of Gastroenterology and Hepatology, University Medical Center Groningen, Groningen, the Netherlands; 65 Department of Internal Medicine and Radboud Center of Infectious Diseases, Radboud University Medical Center, Nijmegen, the Netherlands; 66 Department of General Internal Medicine and Psychosomatics, University Hospital Heidelberg, Heidelberg, Germany; 67 Department of Gastroenterology, Infectious Diseases and Intoxications, University of Heidelberg, Heidelberg, Germany; 68 Department of Psychosomatic Medicine, University Hospital Tübingen, Tübingen, Germany; 69 Department of Internal Medicine and Gastroenterology, HELIOS Clinic Rottweil, Rottweil, Germany; 70 Israelitisches Krankenhaus, Hamburg, Germany; 71 Helios Klinik Krefeld, Krefeld, Germany; 72 Krankenhaus Vilsbiburg, Vilsbiburg, Germany; 73 Department of Psychosomatic Medicine, Division of Internal Medicine, University Hospital Basel, Basel, Switzerland; 74 Faculty of Medicine, University of Basel, Basel, Switzerland; 75 Institute of Human Genetics, University of Heidelberg, Heidelberg, Germany; 76 Interdisciplinary Center for Neurosciences, Heidelberg University, Heidelberg, Germany; 77 Institute of Epidemiology, Christian-Albrechts-University Kiel, Kiel, Germany; 78 Department of Clinical Science, Faculty of Medicine, University of Bergen, Bergen, Norway; 79 Research Unit for General Practice, NORCE Norwegian Research Centre, Bergen, Norway; 80 Department of Global Public Health and Primary Care, University of Bergen, Bergen, Norway; 81 KG Jebsen Center for Genetic Epidemiology, Department of Public Health and Nursing, Faculty of Medicine and Health Sciences, Norwegian University of Science and Technology, Trondheim, Norway; 82 KG Jebsen Center for Genetic Epidemiology, Department of Clinical and Molecular Medicine, Faculty of Medicine and Health Sciences, Norwegian University of Science and Technology, Trondheim, Norway; 83 Florey Institute of Neuroscience and Mental Health, Heidelberg, Victoria Australia; 84 Department of Neurosciences, Katholieke Universiteit Leuven, Leuven, Belgium; 85 Translational Research Center for Gastrointestinal Disorders, Katholieke Universiteit Leuven, Leuven, Belgium

**Keywords:** Genome-wide association studies, Irritable bowel syndrome, Psychiatric disorders

## Abstract

Irritable bowel syndrome (IBS) results from disordered brain–gut interactions. Identifying susceptibility genes could highlight the underlying pathophysiological mechanisms. We designed a digestive health questionnaire for UK Biobank and combined identified cases with IBS with independent cohorts. We conducted a genome-wide association study with 53,400 cases and 433,201 controls and replicated significant associations in a 23andMe panel (205,252 cases and 1,384,055 controls). Our study identified and confirmed six genetic susceptibility loci for IBS. Implicated genes included *NCAM1*, *CADM2*, *PHF2/FAM120A*, *DOCK9*, *CKAP2/TPTE2P3* and *BAG6*. The first four are associated with mood and anxiety disorders, expressed in the nervous system, or both. Mirroring this, we also found strong genome-wide correlation between the risk of IBS and anxiety, neuroticism and depression (*r*_g_ > 0.5). Additional analyses suggested this arises due to shared pathogenic pathways rather than, for example, anxiety causing abdominal symptoms. Implicated mechanisms require further exploration to help understand the altered brain–gut interactions underlying IBS.

## Abstract (web summary)

Genome-wide association analysis of irritable bowel syndrome identifies genetic susceptibility loci and highlights shared pathways with mood and anxiety disorders.

## Main

IBS is common worldwide and typically presents in early adulthood with symptoms including abdominal pain, bloating and bowel dysfunction^1^. Symptom intensity varies over time and between individuals but IBS has been reported, in severe cases, to affect quality of life as much as renal impairment or diabetes^2^.

IBS accounts for approximately half of all referrals to gastroenterology clinics^3^. Although of doubtful clinical value, many patients undergo multiple investigations, including colonoscopies, to exclude conditions such as Crohn’s disease or cancer. To the frustration of patients and clinicians alike, all tests are characteristically normal. The healthcare costs, combined with indirect employment costs, of IBS amount to at least €13 billion (£11.7 billion) annually in Europe^4^.

Attempts have been made to identify positive clinical diagnostic features and reduce investigations. The widely used and recently revised Rome III criteria define IBS as unexplained abdominal pain or discomfort eased by defecation, with altered stool form or frequency, for more than six months^5^. Three main subtypes are recognized: constipation-predominant (IBS-C), diarrhea-predominant (IBS-D) or ‘mixed’/alternating constipation and diarrhea (IBS-M). Individuals with functional constipation and functional diarrhea share the disordered bowel pattern with IBS but do not suffer from pain.

The commonly used IBS treatments, ranging from dietary exclusion to psychoactive medications, are relatively ineffective and their variety reflects the uncertain etiology^6^. Behavioral therapies, while more effective (number needed to treat = 4), are not widely available^7^. Given the high frequency, impact and cost of IBS, there is a pressing need for improved pathophysiological understanding to enable better therapeutic approaches.

The relative importance of peripheral and gut versus central and psychological factors to IBS etiology is uncertain. The consensus view is that IBS results from abnormal brain–gut interactions. Recent epidemiological data suggested that, in individuals developing both IBS and psychological features, the former preceded the latter in two thirds of cases and the latter preceded the former in one third^8^. IBS is associated with abnormalities of central pain processing but also increased gut permeability, mast cell activation, disordered motility and dysbiosis^9^. Up to one in ten cases are triggered after infection, so-called postinfectious IBS (PI-IBS)^10^.

IBS aggregates in families, with individuals being two to three times more likely to develop IBS if they have an affected relative. Estimates of heritability from twin studies range widely from 0 up to 57%^11^. Twin studies have indirectly investigated whether IBS and mental health conditions share a genetic basis but have proved inconclusive^12^. Although genetic association studies have provided pathogenic and therapeutic insights for many conditions^13^, only one variant (rs10512344) has previously been identified at genome-wide significance in IBS, with only modest replication^14^. Larger datasets are clearly required to characterize the recognized heritable component of IBS; the use of large population-based cohorts had been proposed^11,15^. Broad meta-analysis of cases from different cohorts and case definitions is also a proven method of increasing power^16^. Between 2006 and 2010, UK Biobank (UKB) recruited half a million people aged 40–69 years. Participants underwent baseline assessment and consented to long-term follow-up, including questionnaires, and linkage to routinely collected health data. All participants underwent genome-wide SNP genotyping. Therefore, UKB provides a powerful epidemiological resource for exploring risk factors for health outcomes^17,18^.

The main aim of the current study was to identify genetic risk factors for IBS through an analysis involving over 250,000 affected individuals. We report robustly validated genetic susceptibility loci for IBS and provide evidence of its shared genetic etiology with mood and anxiety disorders.

## Results

### Epidemiology

We designed a digestive health questionnaire (DHQ) for the UKB website, with a link e-mailed to 332,793 UKB participants with valid e-mail addresses. A total of 171,061 (51.4%) responses were received and analyzed (Supplementary Fig. 1). The DHQ included validated instruments for IBS diagnosis (Rome III symptom criteria), the IBS symptom severity score (IBS-SSS, measured using the IBS Severity Scoring System)^19^ and the Patient Health Questionnaire 12 Somatic Symptom score (PHQ-12)^20^. It also asked about previous IBS diagnosis, environmental exposures and associated conditions (including anxiety or depression, based on treatment sought or offered).

**Fig. 1:**
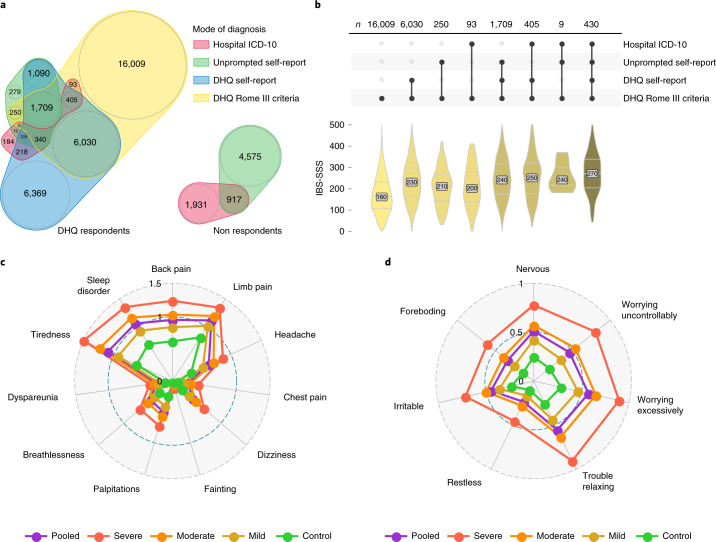
Diagnostic modalities and comorbidities of IBS. **a**, Venn diagram of overlap between UKB IBS cases by different diagnostic modality, split by DHQ respondents and nonrespondents. The areas and numbers indicate the sample size. Most participants with current symptoms (DHQ Rome III, yellow) did not report being diagnosed with IBS either when listing medical conditions unprompted at UKB enrollment (unprompted self-report, green) or when asked specifically about a previous IBS diagnosis when completing the DHQ (DHQ self-report, blue). Conversely, many participants previously diagnosed with IBS, even those formally recorded during a hospital admission (hospital ICD-10, red), did not have symptoms sufficient for Rome III criteria IBS diagnosis at the time of their DHQ response. **b**, Among individuals experiencing IBS symptoms (DHQ Rome III positive), those previously diagnosed by a clinician had greater symptom severity, with an increase in the number of IBS diagnostic modalities (connected dots, middle; top: sample size is shown) being associated with an increase in symptom severity score (IBS-SSS, bottom). Distributions are colored by the number of diagnoses and the groups shown are mutually exclusive. For post-hoc statistics, see Supplementary Table 3. **c**, Severity of different somatic symptoms in the past three months among digestively healthy controls and IBS cases (classified as mild, moderate and severe based on IBS-SSS). Mean scores for PHQ-12 items ranked from 0 (not bothered at all) to 2 (bothered a lot) are shown. Pooled refers to all UKB cases in the discovery cohort. **d**, As above, for symptoms of anxiety in the last two weeks, measured using average scores for GAD-7 items ranked from 0 (never bothered) to 3 (bothered nearly every day).

After sample exclusions and quality control, we identified a total of 40,548 UKB participants of European ancestry (Fig. 1a) who met the diagnostic criteria for IBS (based on DHQ Rome III symptom data, self-report of previous medical IBS diagnosis or electronic medical records; see Methods and Supplementary Tables 1 and 2). The demographics of the DHQ respondents are presented in Table 1. Females were affected by IBS more commonly than males (72.1%). IBS-M, with hard and loose stools present at least ‘sometimes’ (alternating), was the most common subtype in patients defined according to the Rome III criteria (55.0%).

**Table 1:** Demographics, symptom severity, family history and associated conditions for IBS patients diagnosed via different modalities and with different IBS subtypes

						Scores among DHQ participants completing the relevant sections			Among DHQ participants (%)					Atopy (%)
Group	*n*	Male (%)	Female (%)	Age (years)	Age (s.d.)	Mean IBS-SSS	Mean PHQ-12	Mean GAD-7	Family history of IBS	Childhood antibiotic exposure	Born by Cesarean section	Treatment for anxiety offered or sought	Treatment for depression offered or sought	Pooled asthma/hay fever/eczema
Controls (DHQ respondents)	72,356	50.3	49.7	65.3	7.5	33	4.0	1.5	9.5	9.6	2.5	16.1	18.0	18.1
Hospital ICD-10	4,237	23.4	76.6	65.9	7.9	202*	7.4*	3.6*	31.8*	24.0*	2.5	43.0*	43.6*	29.3*
Unprompted self-report	9,309	25.7	74.3	65.2	8.0	196*	7.1*	3.3*	30.7*	19.8*	2.3	41.1*	40.0*	29.6*
DHQ, Rome III criteria	24,845	27.9	72.1	63.9	7.7	194*	7.1*	3.3*	24.3*	20.4*	2.6	33.7*	35.1*	24.0*
DHQ, self-report	16,289	26.2	73.8	64.0	7.7	177*	6.9*	3.1*	29.0*	21.6*	2.7	39.7*	39.3*	25.9*
Pooled (any of the four definitions above)	40,548	27.9	72.1	64.3	7.8	173*	6.8*	3.1*	24.0*	20.0*	2.6	34.3*	35.2*	24.8*
DHQ, Rome III criteria, type C	3,989	16.9	83.1	64.5	7.8	190*	6.8*	3.2*	22.2*	19.2*	2.2	32.3*	34.1*	21.4*
DHQ, Rome III criteria, type D	6,506	33.2	66.8	63.8	7.7	185*	6.5*	3.0*	23.8*	19.1*	2.8	32.1*	33.0*	24.0*
DHQ, Rome III criteria, type M	13,666	28.5	71.5	63.8	7.8	204*	7.5*	3.6*	25.6*	21.5*	2.6	35.2*	36.9*	24.8*
DHQ, Rome III criteria, type U	672	31.0	69.0	65.6	7.5	124*	5.6*	2.4*	16.7*	17.7*	2.8	27.5*	24.9*	23.8*
DHQ, postinfectious	860	24.2	75.8	62.2	7.8	196*	7.4*	3.6*	30.3*	27.7*	3.5	42.0*	42.4*	26.6*
DHQ, functional constipation	3,502	33.9	66.1	67.1	7.4	64*	4.7*	1.8*	12.5*	11.0	2.5	23.1*	25.4*	19.0
DHQ, functional diarrhea	5,386	61.0	39.0	65.1	7.5	49*	4.3*	1.8*	11.0*	10.7*	2.6	20.3*	22.0*	20.5*

Digestively healthy controls and functional constipation as well as diarrhea groups are shown for reference. Gastrointestinal symptoms are captured by the IBS-SSS (range 0–500), while somatic symptoms are captured by the (modified) PHQ-12 (range 0–22). The GAD-7 score captures symptoms of anxiety (range 0–21). The single asterisk marks significant differences from the control group after adjusting for age, sex, DHQ participation and (Bonferroni) multiple testing at *P* < 0.05/108 (two-sided logistic regression test). Age and sex differences were not tested.

A total of 24,845 respondents reported current abdominal symptoms meeting standard diagnostic criteria (DHQ Rome III, Fig. 1) at the time of the survey, providing a point prevalence of IBS of 14.5%. Of these, only 8,836 (35.6%) had a hospital-documented IBS diagnosis, via an IBS ICD-10 code (International Statistical Classification of Diseases and Related Health Problems, 10th revision), or reported having been diagnosed with IBS by a doctor (DHQ self-report and/or unprompted self-report). They reported greater gastrointestinal symptom severity (quantified via IBS-SSS) than those not medically diagnosed (odds ratio (OR) and 95% confidence interval (CI) = 1.07 (1.07–1.08) per IBS-SSS unit); Fig. 1b and Supplementary Table 3).

### Risk factors and associated conditions

As reported previously, a family history of IBS was more common in cases than controls (24.0 versus 9.5%, OR and 95% CI = 3.73 (3.60–3.88)). However, birth by Cesarean section was not (2.6 versus 2.5%, OR and 95% CI = 1.02 (0.94–1.11); Table 1). A significantly higher proportion of cases with IBS recalled receiving long-term or recurrent antibiotics during childhood compared with controls (20.0 versus 9.6%, OR and 95% CI = 2.22 (2.13–2.30)). The severity of current IBS symptoms correlated positively with recalled childhood antibiotic exposure (OR and 95% CI = 1.04 (1.04–1.04) per IBS-SSS unit) and family history (OR and 95% CI = 1.05 (1.05–1.06)). Participants with anxiety also reported increased antibiotics use in childhood (18.4%, OR and 95% CI = 1.64 (1.59–1.70); Supplementary Table 4).

Regarding comorbidities, as documented previously, the rates of appendicectomy, cholecystectomy and hysterectomy were all increased in IBS (Supplementary Table 5), as were the rates of atopic disease (Table 1). Anxiety and depression were each approximately twice as common (Table 1 and Supplementary Table 4); 34.3% of cases reported treatment for anxiety compared with 16.1% of controls. This effect was more prominent in individuals medically diagnosed with IBS.

The median PHQ-12 score was 4 (interquartile range (IQR) = 2–6) in controls and 6 (IQR = 4–9) in pooled cases (7 in all four constituent subgroups, Supplementary Table 6; see also ‘Median scores among pooled and individual diagnoses’ in the Supplementary Note). The PHQ-12 score correlated with IBS symptom severity (Pearson’s correlation = 0.40 (95% CI = 0.39–0.41) among 31,402 IBS cases completing all PHQ-12 and IBS-SSS questions), with back pain, limb pain and tiredness driving this association (Fig. 1c and Supplementary Figs. 2 and 3). Among UKB participants with previous generalized anxiety disorder-7 (GAD-7) scores (*n* = 79,430; Supplementary Table 7) or Patient Health Questionnaire-9 (PHQ-9) depression scores (*n* = 79,087, Supplementary Table 8; see Supplementary Note for definitions), these scores were consistently higher in cases with IBS than controls (OR and 95% CI = 1.14 (1.14–1.15) per GAD-7 unit and 1.15 (1.15–1.16) per PHQ-9 unit) and correlated with IBS-SSS (Pearson’s correlations and 95% CIs among cases = 0.24 (0.22–0.25) and 0.27 (0.25–0.28), respectively; Fig. 1d and Supplementary Fig. 4).

The respective prevalence for functional constipation and functional diarrhea (that is, bowel disturbance without abdominal pain or discomfort; see ‘Definitions of IBS cases’ in the Supplementary Note) were 6.4% and 11.7% (Table 1). Somatic symptoms (PHQ-12) and treatment for anxiety or depression were less strongly associated with functional diarrhea than with IBS-D (excess OR and 95% CI = 1.24 (1.22–1.26) per PHQ-12 unit and 1.58 (1.46–1.72), respectively), with similar effects for functional constipation and IBS-C (Table 1).

### Genetics

**Fig. 2:**
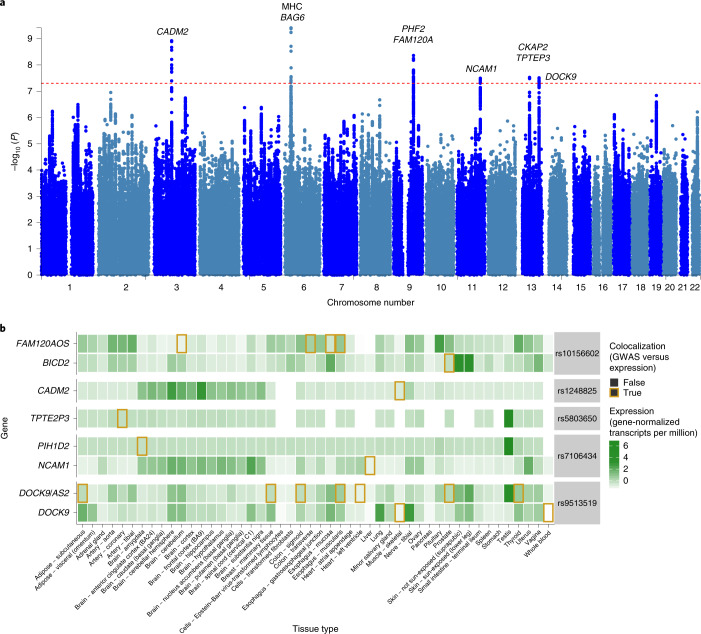
Genome-wide association results for IBS. **a**, Manhattan plot showing the distribution of IBS-associated SNPs across the genome. The dashed line indicates the genome-wide significance threshold at *P* = 5 × 10^−8^. *P* values are from a two-sided test, after inverse variance-weighted fixed-effects meta-analysis. See Supplementary Fig. 5 for zoomed-in visualizations with linkage disequilibrium data, posterior probabilities of causality and transcript annotations. **b**, Expression of genes implicated in IBS etiology through causal effects of associated variants on gene expression across a range of tissues. Darker green indicates higher gene expression and the golden outlines indicate tissue–gene combinations where the IBS-associated variant was known to influence gene expression in this tissue (colocalization posterior > 0.5). Genes with at least one colocalization event across tissues with expression quantitative trait locus data from the GTEx dataset are shown.

We identified six independent IBS susceptibility loci at genome-wide significance (*P* < 5 × 10^−8^) in a discovery cohort totaling 53,400 cases and 433,201 controls (Fig. 2a and Supplementary Fig. 5). This resulted from pooling IBS cases across all case definitions to maximize power, in a meta-analysis of data from UKB (40,548 cases and 293,220 controls; Supplementary Tables 1 and 2) and the international collaborative Bellygenes initiative (12,852 cases and 139,981 controls; Methods and Supplementary Table 9). Using data from an independent panel from 23andMe (Supplementary Note), all six loci were replicated at Bonferroni significance (*P* < 0.0083) with the same direction of effect (Table 2). All were found on autosomal chromosomes (none on the X chromosome) and conferred modest ORs < 1.05. Three out of six loci also had reported associations with mood and anxiety disorders and related phenotypes^21–25^.

**Table 2:** Variants associated with IBS, their effect measured in the discovery cohort and *P* values for association in the discovery cohort, the replication cohort and the meta-analysis of these two

Variant					Effect (discovery)		*P* values			Annotation	
SNP	Chromosome number	Position	Alleles	Frequency	OR	95% CI	Discovery	Replication	Meta-analysis	Mapped gene	Previously implicated in
rs1248825	3	84,993,411	C/A	0.33	1.05	1.03–1.07	1.20 × 10^−9^	4.90 × 10^−8^	7.48 × 10^−15^	*CADM2*	Personality traits (risk-taking, neuroticism, anxiety)^21^, cannabis use^22^
rs2736155	6	31,605,199	G/C	0.48	1.05	1.02–1.07	3.88 × 10^−10^	8.28 × 10^−6^	3.19 × 10^−12^	*BAG6*	
rs10156602	9	96,345,328	G/A	0.63	1.04	1.02–1.06	4.36 × 10^−9^	1.18 × 10^−8^	3.04 × 10^−15^	*PHF2, FAM120AOS*	Neuroticism^23^, depression^23^, autism^24^
rs7106434	11	112,860,579	C/T	0.41	1.04	1.02–1.06	3.19 × 10^−8^	2.27 × 10^−5^	9.17 × 10^−11^	*NCAM1*	Neuroticism^23^, depression^25^, cannabis use^22^, anorexia nervosa^40^
rs5803650	13	53,939,598	CT/C	0.48	1.05	1.03–1.07	2.97 × 10^−8^	2.25 × 10^−8^	6.31 × 10^−14^	*CKAP2, TPTE2P3*	
rs9513519	13	99,610,146	G/A	0.62	1.04	1.02–1.06	3.09 × 10^−8^	4.20 × 10^−5^	2.31 × 10^−10^	*DOCK9*	

The reported frequencies and effects are those of the second allele. The second allele is defined such that it increases IBS risk. Allele frequencies are taken from UKB. Previous associations were obtained from the literature and GWAS Catalog (Supplementary Note).

We undertook genetic fine-mapping to establish plausible causal variants (Supplementary Fig. 5) and used several techniques to identify candidate causal genes within IBS risk loci (Supplementary Table 10; see ‘Gene mapping’ in the Supplementary Note). Among the genes implicated (Table 2) were two encoding neural adhesion molecules: neural cell adhesion molecule 1 (*NCAM1*) and cell adhesion molecule 2 (*CADM2*). Ranking tissues according to enrichment for risk gene expression (Supplementary Fig. 6), the brain came top of the list (LDSC applied to specifically expressed genes^26^ coefficient = 8.32 × 10^−10^, s.e.m. = 4.5 × 10^−10^, *P* = 0.03). However, this result was not statistically significant after correcting for multiple testing, which may in part reflect lack of power due to low SNP heritability. Using expression colocalization analysis as a separate method to implicate specific gene–tissue combinations, we found evidence that the six IBS-associated variants regulate gene expression across a number of tissues, with many genes particularly expressed in the brain (Fig. 2b).

One association mapped to the major histocompatibility complex (MHC) class 3 region close to BAG cochaperone 6 (*BAG6*). The signal is not driven by human leukocyte antigen (HLA) alleles and is independent of known MHC associations with ulcerative colitis, celiac disease or microscopic colitis (Supplementary Fig. 7 and Supplementary Tables 11 and 12) (refs. ^27–30^). It is also independent of lead variants for neuroticism at this locus (highest *r*^2^ = 0.51)^23^.

Eight additional loci showed genome-wide significant association with various IBS definitions (Methods) but not the whole discovery cohort, of which five were replicated in the 23andMe data (Supplementary Fig. 8 and Supplementary Tables 13 and 14). These require further study. The female-specific signal identified previously^14^ for unprompted self-reported IBS in the UKB was also observed in our female-specific analysis of unprompted self-reported data but was not detected in female-specific analyses of any other case definitions from UKB or Bellygenes initiative, nor replicated in the 23andMe unstratified analyses of both sexes (Supplementary Table 15), possibly suggesting survey-specific factors playing a role. Specific candidate gene associations previously reviewed in the literature^31,32^ also did not show significant evidence of association after multiple testing correction (all *P* > 0.015).

LDSC estimated a modest but significant genome-wide SNP heritability for IBS of 5.77% (s.e.m. = 0.35%) in the discovery cohort, with no evidence of population stratification (LDSC intercept = 0.9951, s.e.m.= 0.007). This was consistent across case definitions within UKB (*h*^2^ range of 5.42–7.71%), with similar values seen in the Bellygenes (*h*^2^ = 3.14%, s.e.m. = 0.74%) and 23andMe cohorts (*h*^2^ = 5.39%, s.e.m.= 0.02%).

IBS-C showed weak genetic correlation with functional constipation, as did IBS-D with functional diarrhea (Supplementary Fig. 9). IBS-C and IBS-D correlated with each other but there were no cross-correlations, that is, IBS-C did not correlate with functional diarrhea. Heritability for the IBS subtypes was comparable with IBS overall; IBS subtypes showed similar genetic correlation with mental health and personality traits (Supplementary Table 16).

**Fig. 3:**
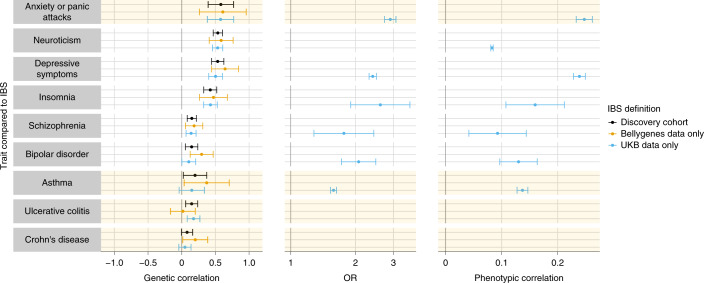
Genetic and phenotypic correlations between IBS and other traits. Correlations of genetic risk (coheritability estimates, left) and phenotype (ORs, middle, and liability-scale correlation, right) between IBS and other traits. The subset of all LD Hub traits (excluding rapid GWAS results) with significant genetic correlation (two-tailed coheritability test as implemented in the LDSC with unadjusted *P* < 0.05) in both UKB data (light blue, 40,548 cases and 293,220 controls) and the independent Bellygenes cohort (orange, 12,852 cases and 139,981 controls) is shown, as well as the meta-analysis used for discovery (black, 53,400 cases and 433,201 controls). IBS had a similar genetic risk profile to traits such as neuroticism, depression and insomnia (for which *P* values were significant in all datasets after multiple testing correction for the number of traits tested). Traits highlighted in yellow were added manually given their clinical relevance. Anxiety was not initially included since it is only available in the LD Hub as a rapid GWAS result. Among UKB participants, we present ORs (middle) and phenotypic correlations values (right) for these traits. Sample sizes (restricted to UKB participants who were either cases or controls in the discovery cohort; *n* = 333,768) were as follows: anxiety or panic attacks, 8,714; neuroticism, 271,423 (scores); depressive symptoms, 24,311; insomnia, 326; schizophrenia, 574; bipolar disorder, 1,207; asthma, 41,178.

We compared the overlap between susceptibility with IBS and 751 other traits and diseases listed in the LD Hub^33^. The strongest correlations in genome-wide risk were with mood and anxiety disorders and related phenotypes, including anxiety (*r*_g_ = 0.58, s.e.m. = 0.10), neuroticism (*r*_g_ = 0.54, s.e.m. = 0.04), depression (*r*_g_ = 0.53, s.e.m. = 0.05) and insomnia (*r*_g_ = 0.42, s.e.m. = 0.05)^33^. Across the genome, the same alleles that predisposed to IBS also predisposed to mood and anxiety disorders. The correlations were consistent regardless of the mode of diagnosis of anxiety or depression (Supplementary Fig. 10) (refs. ^34,35^). We calculated phenotypic correlations for these traits on a comparable liability scale (Fig. 3 and Supplementary Table 16). Mostly, the phenotypic and genotypic correlations mirrored each other, although genetic correlations were often larger. Notably, other digestive diseases presenting with similar symptoms, including celiac disease (*r*_g_ = 0.03, s.e.m. = 0.08, *P* = 0.69) and Crohn’s disease (*r*_g_ = 0.08, s.e.m. = 0.04, *P* = 0.06), were not genetically correlated with IBS.

We also ran higher-specificity (IBS cases meeting at least 2 of the 4 UKB case definitions, 11,201 cases and 293,220 controls) and high-severity (IBS-SSS > 300, 4,296 cases and 72,356 controls) analyses in UKB. The former produced no new associations. The latter, while being more heritable (liability scale *h*^2^ = 0.42, s.e.m. = 0.05, Cochran’s *Q* = 51.7, *P* = 6.31 × 10^−13^ compared with the discovery cohort IBS), produced one association (rs9947289, *P* = 2.80 × 10^−8^) that did not replicate (*P* = 0.57 in the 23andMe data; Supplementary Table 13). Both of these phenotypes recapitulated the same genetic correlation with mood and anxiety disorders as found in the discovery cohort (Supplementary Fig. 11).

To explore the role of shared genetic risk versus direct phenotypic overlap, we compared the genome-wide association study (GWAS) results for IBS having removed participants with anxiety to the GWAS results for anxiety having removed participants with IBS (for anxiety definitions, see Supplementary Tables 17 and 18). The genetic correlation between IBS and anxiety attenuated but remained strong (*r*_g_ = 0.31, s.e.m. = 0.06; Supplementary Fig. 12). We next used bidirectional Mendelian randomization^36^ with an independent anxiety GWAS^37^, as well as genome-wide latent variable Mendelian randomization^38^, to explore directionality. Multiple models could explain our data (Supplementary Table 19) but they were best explained by shared genetic risk pathways rather than causal effects between the two traits. Similar complex causal relationships were evident between IBS and mental health and personality traits other than anxiety (Supplementary Table 19).

## Discussion

The importance of this study lies in its scale and therefore the robustness of its genetic results. We have identified replicable genetic associations for IBS, providing new biological insights, while demonstrating that overall its heritability is modest. Two observations are particularly striking: the genetic overlap between IBS and mood and anxiety disorders and the lack of signals implicating genes expressed specifically in the gut or overlapping other intestinal disorders. Our findings suggest that, with respect to the genetically determined risk for IBS, neuronal pathways play a dominant role.

Increasing abdominal symptom severity correlated with increasing PHQ-12 somatic symptom scores, particularly for the domains of tiredness, back pain, limb pain and headache (Fig. 1). Multifocal pain suggests either poor coping skills, perhaps relating to psychological comorbidity, or visceral hypersensitivity from aberrant antinociceptive mechanisms^39^. By contrast, the painless bowel disorders, functional constipation and functional diarrhea, were less strongly associated with raised PHQ-12 scores or psychological comorbidity.

IBS showed the strongest genome-wide overlap with psychological traits: anxiety, neuroticism, depression and schizophrenia (Fig. 3). GAD-7 anxiety scores correlated with IBS severity (Fig. 1) and 34.3% of cases with IBS had sought or had been treated for anxiety versus 16.1% of controls (Table 1). Although the phenotypic correlation was strong, the genetic correlation appeared quantitatively even greater (Fig. 3). Furthermore, this genetic correlation between IBS and anxiety persisted even after eliminating data from individuals with phenotypic overlap (that is, between GWAS for ‘IBS excluding anxiety’ and ‘anxiety excluding IBS’; Supplementary Fig. 12). Thus, their co-occurrence probably reflects shared etiologic pathways between IBS and anxiety rather than one condition simply causing the other. This conclusion was supported by the Mendelian randomization analysis.

Four out of six of the confirmed IBS loci implicated genes influencing mood or anxiety disorders, genes expressed in the nervous system or both. These include *NCAM1* (also associated with neuroticism, anxiety, mood disorders and anorexia nervosa)^23,25,40^, *CADM2* (also associated with neuroticism, anxiety and cannabis use)^21,22^, PHD finger protein 2 (*PHF2*)/family with sequence similarity 120A (*FAM120A*) (also associated with neuroticism, depression and autism)^23,24^ and dedicator of cytokinesis 9 (*DOCK9*). Brain expression of *NCAM1* and *CADM2* was implicated in our colocalization analysis (Fig. 2b and Supplementary Table 10): both regulate neural circuit formation and influence changes in white matter microstructure found in both mood disorders and IBS^25,41,42^. *PHF2* and *DOCK9* also play key roles in brain development^43,44^. Of note, *NCAM1*, *PHF2* and *DOCK9* are also expressed in the rich network of nerve fibers and ganglia of the gut, while *CADM2* is not^45^. Predominant brain expression, combined with the coassociation of IBS with several psychological traits, perhaps most strongly implicates the central nervous system as the site where these gene variants exert their action. However, the genetic variants may also be acting peripherally for the subset expressed in the enteric nervous system, which shares many neurotransmitters, signaling pathways and anatomical properties as well as rich communication with the brain.

The MHC signal is independent of known HLA associations with ulcerative colitis and celiac disease; in fact, it localizes to *BAG6* (Supplementary Fig. 7 and Supplementary Tables 10–12). BAG6 is known to chaperone misfolded proteins, regulate membrane protein dynamics and affect diverse processes from apoptosis to antigen presentation^46,47^. Functional exploration of *BAG6* may yield new IBS pathophysiological insights unconnected to the nervous system.

IBS genome-wide SNP heritability was just 5.8% (s.e.m. < 0.01) in the European ancestry population in this study and the effect sizes of our susceptibility loci were modest (OR < 1.05). Earlier genetic studies of IBS were underpowered to detect such small effects. By comparison, SNP heritability estimates for Crohn’s disease, ulcerative colitis and anxiety are 41%, 23% and 26%, respectively^48,49^. Previous IBS heritability estimates, from family and twin studies, varied widely at 0–57% (ref. ^11^). Our results indicate that the genetic contribution to IBS heritability is modest and imply that additional environmental factors, including dysbiosis, diet, stress and learned behaviors, all potentially shared within families, play a more prominent role.

Regarding dysbiosis, we noted increased childhood exposure to antibiotics among IBS cases (20.0%) versus controls (9.6%). While there are clearly biases inherent to recall of events from childhood, this result is corroborated by previous studies specifically set up to address this question^50^. Interestingly, we saw the same association with anxiety (18.4%). Among possible explanations, childhood antibiotics might increase the risk of IBS (and perhaps anxiety) by embedding a dysbiotic gut flora and disturbing the balance of short-chain fatty acid metabolites known to influence microglial development and mood^50,51^. Equally, anxiety in late adulthood might influence recall of childhood antibiotic exposure, and familial anxiety might lead parents to take their offspring to the doctor repeatedly for minor ailments, resulting in recurrent antibiotic exposure. While enteric infection can alter the baseline gut microbiota and trigger PI-IBS, in the UKB PI-IBS closely mirrored ‘conventional’ IBS in terms of symptom severity, frequency of family history and association with psychological traits, suggesting that the infectious ‘seed’ falls on fertile ground to trigger IBS in predisposed individuals.

One question is whether the neuronal emphasis of our results derives from our strategy of combining multiple IBS definitions to increase statistical power, including pooling ‘opposite’ subtypes (for example, IBS-C and IBS-D), that is, whether gut-specific effects might be lost in the pooling such that the brain remains the common link between these. However, the heritability of IBS subtypes is comparable with IBS overall; IBS-C and IBS-D share approximately 50% of their genetic susceptibility and each of the subgroups also individually genetically correlates with mental health and personality traits (Supplementary Table 16). Furthermore, subtype GWAS identified only one significant signal in IBS-C and none in IBS-D, suggesting an absence of strong subtype-specific, possibly gut-focused genetic effects.

Aside from the pooling strategy, justified by our LDSC analysis (Methods), other potential weaknesses include the use of Rome III criteria instead of the more restricted Rome IV criteria, since the former were the standard at the time of study design^52^, the fact the IBS diagnosis was made based on Rome III symptoms reported via the DHQ rather than by medical review for nearly half of cases in the UKB cohort and the limited age range and ancestry of UKB. However, we believe that the fact that all of the loci identified at genome-wide significance thresholds in the discovery panel replicated in the independent 23andMe panel validates both the findings and the approach taken.

Our GWAS and the results of our polygenic analyses provide important new insights. Individual loci identified by the GWAS implicate new target genes within previously under-researched pathways (for example, neuronal adhesion). Mendelian randomization and genome-wide correlation analyses demonstrate shared genetic risk pathways between anxiety and IBS that are independent of the comorbidity between these two traits. This may point toward a mechanistic rationale for the efficacy of psychoactive medications and behavioral therapies and suggest that more attention should be paid to identifying new therapeutics that target neuronal function. We anticipate that future research will build on our discoveries, both by investigating the target genes identified and exploring the shared genetic risk across traits to improve our understanding of the disordered brain–gut interactions that characterize IBS.

## Methods

For details of the cohorts, descriptive statistics, association analysis methodologies and functional interpretation of associations, see the Supplementary Note.

### Ethics oversight

The UKB DHQ was approved as a substantial amendment to the UKB protocol by the North West – Haydock Research Ethics Committee, reference no. 11/NW/038e. The Bellygenes initiative study received ethical approval from the Stockholm Ethics Examination Authority (EPN ID 2016/1620-31/2) and Monash University Human Research Ethics Committee (MUHREC ID 20326). Written informed consent was obtained from all participants. This study did not award compensation to any participant.

### Data and study participants

Our discovery cohort combined cases of IBS identified in UKB with cases from the Bellygenes initiative. Replication was sought in an independent panel from 23andMe. Cases ascertained in UKB met at least one of the following four conditions (the DHQ is viewable online – UKB resource 595): (1) DHQ Rome III: met Rome III symptom criteria for IBS diagnosis without other diagnostic explanations for these symptoms; (2) DHQ ‘self-report’: answered ‘yes’ to the question ‘Have you ever been diagnosed with IBS?’; (3) Unprompted ‘self-report’: self-reported IBS diagnosis in response to question ‘Has a doctor ever told you that you have any … serious medical conditions?’; (4) hospital ICD-10: linked hospital episode statistics indicating inpatient or day-case admission with clinician diagnosis of IBS entered as main or secondary ICD-10 diagnosis.

Participants with conditions such as celiac disease, inflammatory bowel disease or previous intestinal resectional surgery that could result in IBS-like symptoms were excluded from both cases and digestively healthy controls to avoid signal contamination. For detailed case and control inclusion and exclusion criteria, see Supplementary Tables 1 and 2, respectively. To maximize sample size, cases from the 4 UKB groups were pooled (*n* = 40,548). This approach was supported by demonstrating high genetic correlations between them using LDSC^48^ following a separate GWAS on each (minimum pairwise *r*_g_ = 0.70, s.e.m. = 0.06; Supplementary Fig. 13) and by previous literature on the consistency of genetic results obtained from different diagnostic definitions in UKB^16^.

We then meta-analyzed IBS GWAS data from UKB (40,548 cases) and Bellygenes initiative (12,852 cases and 139,981 controls; Supplementary Table 9), an international collaboration studying IBS genetics based on electronic medical records, specialist diagnoses form tertiary clinics and questionnaire data (including Rome III criteria) across multiple cohorts, having again demonstrated high genetic correlation between them (*r*_g_ = 0.998, s.e.m. = 0.129). This produced a total discovery cohort of 53,400 cases and 433,201 controls. Evidence of replication was sought in a large 23andMe dataset (Supplementary Note). 23andMe cases (*n* = 205,252) self-reported being diagnosed or treated for IBS while controls (*n* = 1,384,055) did not.

Analyses of IBS subtypes were conducted solely using UKB DHQ data based on standard definitions of IBS-C, IBS-D, IBS-M and IBS-U according to the frequency of hard or lumpy stools versus loose, mushy or watery stools. Functional constipation and functional diarrhea cases were identified similarly, and with the same exclusions per IBS cases, but (in contrast to the Rome III definition of IBS) needed to have responded ‘never’ when asked about the frequency of abdominal pain in the last three months. Likewise, analyses of IBS severity (using the IBS-SSS) and associated somatic symptoms (using the PHQ-12) were restricted to DHQ respondents. Anxiety and depression were identified among UKB participants based on previously surveyed responses to GAD-7 anxiety and PHQ-9 depression questionnaires, self-report of diagnosis with depression or anxiety/panic attack, diagnostic codes for major depression and phobic or generalized anxiety disorder in electronic healthcare records or reporting of treatment being sought or offered for these conditions in our DHQ (Supplementary Note).

### Statistical analysis

Association between IBS and nongenetic risk factors, including risk factors assayed by recall from the DHQ, was tested using logistic regression conditioning on age and sex (Supplementary Note, ‘Nongenetic associations’).

Standard genetic quality control was carried out to remove samples with poor genotype quality and variants with poor genotyping or imputation performance. Only participants of European ancestry were included in the discovery dataset due to the limited number of non-European ancestry participants. GWAS were conducted using a linear mixed model (BOLT-LMM v.2.3.2)^53^ to control for population stratification and relatedness. Meta-analysis of GWAS summary statistics was carried out using METAL (March 2011 release)^54^. The UKB GWAS was stratified into DHQ respondents and nonrespondents, with results meta-analyzed to avoid genetic confounding with questionnaire response (Supplementary Fig. 14).

We assigned loci to candidate genes using annotations from FUMA v.1.3.4^55^, as well as from a colocalization analysis using Coloc v.3.2-1^56^ on multi-tissue expression data from the Genotype-Tissue Expression (GTEx) consortium^56,57^. We calculated SNP heritability and coheritability (*r*_g_, genetic correlation) using univariate and bivariate LDSC^48^ against a range of traits via the LD Hub website^33^. Other statistical analyses were carried out using R v.3.6.1; any *P* values were obtained from two-sided tests unless otherwise specified.

### Reporting Summary

Further information on research design is available in the Nature Research Reporting Summary linked to this article.

## Online content

Any methods, additional references, Nature Research reporting summaries, source data, extended data, supplementary information, acknowledgements, peer review information; details of author contributions and competing interests; and statements of data and code availability are available at 10.1038/s41588-021-00950-8.

## Supplementary information

Supplementary Information Supplementary Note and Figs. 1–14.

Reporting Summary

Peer Review Information

Supplementary Tables Supplementary Tables 1–22.

## Data Availability

Genome-wide summary statistics have been deposited to the European Bioinformatics Institute GWAS Catalog (https://www.ebi.ac.uk/gwas/) under accession no. GCST90016564. Individual-level data on the DHQ responses, along with matching genotype, electronic health record and survey data, are available via an application to the UK Biobank Access Management System (https://bbams.ndph.ox.ac.uk/ams/). Individual-level data for 23andMe were not shared as part of this project to protect the privacy of 23andMe participants. Data used for the analyses pertinent to the Bellygenes initiative include both individual-level and aggregate data. Individual-level data from the following sources can be obtained via applications to the respective biobanks and cohorts: TWINGENE (https://ki.se/en/research/swedish-twin-registry-for-researchers); HUNT (https://www.ntnu.edu/hunt/data); Michigan Genomics Initiative (https://precisionhealth.umich.edu/our-research/michigangenomics/); Estonian Genome Center of the University of Tartu (https://genomics.ut.ee/en/biobank.ee/data-access); Lifelines (https://www.lifelines.nl/researcher/how-to-apply); Gene-Environment and Gene-Gene Interaction Research Application (dbGaP Study accession no. phs000674.v2.p2, now superseded by phs000674.v3.p3). Data from IBS patients from tertiary centers can be requested from Mauro D’Amato at mdamato@cicbiogune.es and may be made available depending on specific material and data transfer agreements with principal investigators at respective collaborating institutions.

## Code availability

All software used is publicly available at the URLs or references cited. The R code for additional analyses is available at 10.5281/zenodo.5048820.
