# Supplementary material for: Genome-wide analysis of 53,400 people with irritable bowel syndrome highlights shared genetic pathways with mood and anxiety disorders
Source: Nat Genet. 2021 Nov 5;53(11):1543–52. doi: 10.1038/s41588-021-00950-8 (PMC8571093; doi:10.1038/s41588-021-00950-8)
Supplement: Supplementary file 1 — Supplementary Note and Figs. 1–14. [file 41588_2021_950_MOESM1_ESM.pdf]

nature portfolio

https://doi.org/10.1038/s41588-021-00950-8

Supplementary information

Genome-wide analysis of 53,400 people with irritable bowel syndrome highlights shared genetic pathways with mood and anxiety disorders

In the format provided by the authors and unedited

Supplementary Note for “Genome-wide analysis of 53,400 people with irritable bowel syndrome highlights shared genetic pathways with mood and anxiety disorders”

Table of Contents

Bellygenes initiative banner authorship **5**

Literature review **11**

Description of cohorts **11**

Description of the UK Biobank digestive health questionnaire 11

Definitions of IBS cases 12

DHQ-based Rome III definition 12

DHQ-based prompted self-report of previous IBS diagnosis 13

Unprompted self-report of previous IBS diagnosis 13

Hospital admission ICD-10 diagnosis 13

Definition of IBS and IBS subtypes from DHQ data by Rome III classification criteria 14

Description of UK Biobank control cohorts 15

Description of Bellygenes cohorts 15

Rome-based cohorts 15

The HUNT Study 15

TWINGENE 16

Lifelines 16

ICD-based cohorts 17

Mayo Genome Consortia 17

Genetic Epidemiology Research on Aging 17

Estonian Genome Center at the University of Tartu 17

Michigan Genomics Initiative 17

Tertiary center data 18

Description of 23andMe cohort 18

Definition of post-infectious IBS 19

Sample QC 19

Descriptive statistics **20**

Calculation of IBS symptom severity score 20

Calculation of PHQ-12 somatic symptom score 21

Calculation of GAD-7 anxiety score 22

Calculation of PHQ-9 depression score 22

Median scores among pooled and individual diagnoses 23

Association analyses **24**

Controlling for response bias 24

Non-genetic associations 24

Genetic association testing and SNP filtering 25

Meta-analysis 25

Clumping 25

Conditional analysis 26

Replication 26

Fine mapping 27

HLA association testing 27

Functional interpretation of associations **27**

Gene mapping 27

Gene expression enrichment 28

Comparison to previous GWAS 28

GWAS Catalog 28

Genetic correlation estimates 28

Phenotypic correlation estimates 29

Mendelian randomization analyses 29

References **30**

Supplementary figures **33**

Bellygenes initiative banner authorship

In addition to the authors listed in the main manuscript, contributors to this project under the Bellygenes initiative banner are:

Mauro D'Amato
Center for Molecular Medicine & Clinical Epidemiology Unit, Department of Medicine Solna, Karolinska Institutet, Stockholm, Sweden

School of Biological Sciences, Monash University, Clayton, VIC, Australia

Institute of Clinical Molecular Biology, Christian-Albrechts-University of Kiel, Kiel, Germany

Biodonostia Health Research Institute, San Sebastian, Spain

Gastrointestinal Genetics Lab, CIC bioGUNE - BRTA, Derio, Spain

IKERBASQUE, The Basque Science Foundation, Bilbao, Spain

Tenghao Zheng
Center for Molecular Medicine & Clinical Epidemiology Unit, Department of Medicine Solna, Karolinska Institutet, Stockholm, Sweden

School of Biological Sciences, Monash University, Clayton, VIC, Australia

Ferdinando Bonfiglio
Center for Molecular Medicine & Clinical Epidemiology Unit, Department of Medicine Solna, Karolinska Institutet, Stockholm, Sweden

School of Biological Sciences, Monash University, Clayton, VIC, Australia

Michael Camilleri
Clinical Enteric Neuroscience Translational and Epidemiological Research and Division of Gastroenterology and Hepatology, Department of Medicine, Mayo Clinic, Rochester, MN, USA

Gianrico Farrugia
Enteric NeuroScience Program (ENSP), Division of Gastroenterology and Hepatology, Department of Medicine, Mayo Clinic, Rochester, MN, USA

Purna Kashyap
Department of Gastroenterology and Hepatology, Mayo Clinic, Rochester, MN, USA

Lin Chang
Division of Digestive Diseases/Gastroenterology, David Geffen School of Medicine at UCLA, UCLA, Los Angeles, CA, USA

Emeran Mayer
David Geffen School of Medicine, University of California, Los Angeles (UCLA), Los Angeles, CA, USA

Margaret Heitkemper
Department of Biobehavioral Nursing and Health Informatics, University of Washington, Seattle, WA, USA

Gregory S Sayuk
Division of Gastroenterology, Washington University School of Medicine, St. Louis, MO, USA

Tamar Ringel-Kulka
Gillings School of Global Public Health, University of North Carolina at Chapel Hill, Chapel Hill, NC, USA

Yehuda Ringel
Department of Medicine, Division of Gastroenterology and Hepatology, University of North Carolina at Chapel Hill, Chapel Hill, NC, USA

Division of Gastroenterology and Hepatology, Meir Medical Center, affiliated with Tel-Aviv University, Kfar Saba, Israel

William D Chey
Division of Gastroenterology, Michigan Medicine, Ann Arbor, MI, USA

Shanti Eswaran
Division of Gastroenterology, Michigan Medicine, Ann Arbor, MI, USA

Juanita L Merchant
Division of Gastroenterology, University of Arizona College of Medicine, Tucson, AZ, USA

Robert J Shulman
Children's Nutrition Research Center, Baylor College of Medicine, Texas Children's Hospital, Houston, TX, USA

Luis Bujanda
Department of Gastrointestinal and Liver Diseases, Biodonostia HRI, San Sebastian, Spain

Centro de Investigación Biomédica en Red de Enfermedades Hepáticas y Digestivas (CIBERehd), Instituto Carlos III, Madrid, Spain

Universidad del País Vasco (UPV/EHU), San Sebastian, Spain

Koldo Garcia-Etxebarria
Department of Gastrointestinal and Liver Diseases, Biodonostia HRI, San Sebastian, Spain

Centro de Investigación Biomédica en Red de Enfermedades Hepáticas y Digestivas (CIBERehd), Instituto Carlos III, Madrid, Spain

Aldona Dlugosz
Department of Medicine Solna, Karolinska Institutet, Center for Digestive Diseases, Karolinska University Hospital, Stockholm, Sweden

Greger Lindberg
Department of Medicine Solna, Karolinska Institutet, Center for Digestive Diseases, Karolinska University Hospital, Stockholm, Sweden

Peter T Schmidt
Department of Medicine Solna, Karolinska Institutet, Center for Digestive Diseases, Karolinska University Hospital, Stockholm, Sweden

Pontus Karling
Division of Medicine, Department of Public Health and Clinical Medicine, Umeå University, Umeå, Sweden

Bodil Ohlsson
Lund University, Skåne University Hospital, Department of Internal Medicine, Lund, Sweden

Susanna Walter
Division of Neuro and Inflammation Science, Department of Clinical and Experimental Medicine, Linköping University, Linköping, Sweden

Åshild O Faresjö
Department of Health, Medicine and Caring Science /Society and Health/Public health, Linköpings University, Linköping, Sweden

Magnus Simren
Dept of Internal Medicine & Clinical Nutrition, Institute of Medicine, Sahlgrenska Academy, University of Gothenburg, Gothenburg, Sweden

Jonas Halfvarson
Department of Gastroenterology, School of Medical Sciences, Örebro University, Örebro, Sweden

Piero Portincasa
Department of Biomedical Sciences and Human Oncology (DIMO), Clinica Medica "A. Murri", University of Bari Medical School, Bari, Italy

Giovanni Barbara
Department of Medical and Surgical Sciences, University of Bologna, St. Orsola - Malpighi Hospital, Bologna, Italy

Paolo Usai-Satta
S.C. Gastroenterologia, Azienda Ospedaliera G. Brotzu, Cagliari, Italy

Matteo Neri

Department of Medicine and Aging Sciences and CESI, G. D'Annunzio University & Foundation, Chieti, Italy

Gerardo Nardone
Gastroenterology Unit, Department of Clinical Medicine and Surgery, University Federico II, Naples, Italy

Rosario Cuomo
Gastroenterology Unit, Department of Medical Science, "Sant'Anna e San Sebastiano" Hospital, Caserta, Italy

Francesca Galeazzi
UOC Gastroenterologia, Padova University Hospital, Padova, Italy

Massimo Bellini
Gastroenterology Unit, Department of Gastroenterology, University of Pisa, Pisa, Italy

Anna Latiano
Division of Gastroenterology, IRCCS "Casa Sollievo della Sofferenza" Hospital, San Giovanni Rotondo, Italy

Lesley Houghton
Division of Gastroenterology and Surgical Sciences, Leeds Institute of Medical Research at St. James's, University of Leeds, Leeds, UK

Department of Gastroenterology, Mayo Clinic, Jacksonville, USA

GI Sciences, Division of Diabetes, Endocrinology & Gastroenterology, University of Manchester, UK

Daisy Jonkers
Department of Internal Medicine, NUTRIM School for Nutrition and Translational Research in Metabolism, Maastricht University Medical Center+, Maastricht, The Netherlands

Alexandra Zhernakova
Department of Genetics, University Medical Center Groningen, Groningen, The Netherlands

Alexander Kurilshikov
Department of Genetics, University Medical Center Groningen, Groningen, the Netherlands

Rinse K Weersma
Department of Gastroenterology and Hepatology, University Medical Center Groningen, Groningen, the Netherlands

Mihai Netea
Department of Internal Medicine and Radboud Center of Infectious Diseases (RCI), Radboud University Medical Center, Nijmegen, The Netherlands

Andre Franke

Institute of Clinical Molecular Biology, Christian-Albrechts-University of Kiel, Kiel, Germany

Matthias Hübenthal
Institute of Clinical Molecular Biology, Christian-Albrechts-University of Kiel, Kiel, Germany

Department of Dermatology, Quincke Research Center, University Hospital Schleswig-Holstein, Kiel, Germany

Jonas Tesarz
Department of General Internal Medicine and Psychosomatics, University Hospital Heidelberg, Heidelberg, Germany

Annika Gauss
Department of Gastroenterology, Infectious Diseases and Intoxications, University of Heidelberg, Germany

Miriam Goebel-Stengel
Department of Psychosomatic Medicine, University Hospital Tübingen, Tübingen, Germany
Department of Internal Medicine and Gastroenterology, HELIOS Clinic Rottweil, Rottweil, Germany

Viola Andresen
Israelitisches Krankenhaus, Hamburg, Germany

Thomas Frieling
Helios Klinik Krefeld, Krefeld, Germany

Christian Pehl
Krankenhaus Vilsbiburg, Vilsbiburg, Germany

Rainer Schaefert
Department of Psychosomatic Medicine, Division of Internal Medicine, University Hospital Basel, Basel, Switzerland

Faculty of Medicine, University of Basel, Basel, Switzerland

Beate Niesler
Institute of Human Genetics, University of Heidelberg, Heidelberg, Germany

Interdisciplinary Center for Neurosciences, Heidelberg University, Heidelberg, Germany

Wolfgang Lieb
Institute of Epidemiology, Christian-Albrechts-University Kiel, Kiel, Germany

Kurt Hanevik
Department of Clinical Science, Faculty of Medicine, University of Bergen, Bergen, Norway

Nina Langeland
Department of Clinical Science, University of Bergen, Bergen, Norway

Knut-Arne Wensaas
Research Unit for General Practice, NORCE Norwegian Research Centre, Bergen, Norway

Sverre Litleskare
Department of Global Public Health and Primary Care, University of Bergen, Bergen, Norway

Anne Heidi Skogholt
Department of Laboratory Medicine, Children's and Women's Health, , NTNU, Norwegian University of Science and Technology, Trondheim, Norway

Department of Public Health and Nursing, NTNU, Norwegian University of Science and Technology, Trondheim, Norway

Maiken E Gabrielsen
KG Jebsen Center for Genetic Epidemiology, Department of Public Health and Nursing, Faculty of Medicine and Health Sciences, Norwegian University of Science and Technology, Trondheim, Norway

Laurent Thomas
KG Jebsen Center for Genetic Epidemiology, Department of Clinical and Molecular Medicine, Faculty of Medicine and Health Sciences, Norwegian University of Science and Technology, Trondheim, Norway

Kristian Hveem
Department of Public Health and Nursing, NTNU, Norwegian University of Science and Technology, Trondheim, Norway

Eivind Ness-Jensen
Department of Public Health and Nursing, NTNU, Norwegian University of Science and Technology, Trondheim, Norway

Department of Medicine, Levanger Hospital, Nord-Trøndelag Hospital Trust, Levanger, Norway

Department of Molecular Medicine and Surgery, Karolinska Institutet, Karolinska University Hospital, Stockholm, Sweden

Tõnu Esko
Estonian Genome Center, Institute of Genomics, University of Tartu, Tartu, Estonia

Maris Teder-Laving
Estonian Genome Center, Institute of Genomics, University of Tartu, Tartu, Estonia

Vincent Thijs
Florey Institute of Neuroscience and Mental Health, Heidelberg, Victoria, Australia

Robin Lemmens

Department of Neurosciences, KU Leuven, Leuven, Belgium

Lukas Van Oudenhove
Translational Research Center for Gastrointestinal Disorders, KU Leuven, Leuven, Belgium

Mira Wouters
Translational Research Center for Gastrointestinal Disorders, KU Leuven, Leuven, Belgium

Guy Boeckxstaens
Translational Research Center for Gastrointestinal Disorders, KU Leuven, Leuven, Belgium

Literature review

As a way of assessing previous evidence on the role of genetics of IBS and its heritability, we searched PubMed for any twin, family or genetic studies involving IBS and manually reviewed 139 publications from the last decade, excluding candidate gene studies without replicated results. The exact PubMed query used was "*(IBS[Title] OR (irritable bowel syndrome[Title])) AND ((genom*[Title]) OR (famil*[Title]) OR (twin*[Title]) OR (heritab*[Title]) OR (GWAS[Title]) OR (genetic*[Title]) OR (variant*[Title]) OR (locus[Title]) OR (loci[Title]) OR (polymorphism*[Title]))*".

Our search revealed that IBS aggregates in families, with individuals being 2-3 times more likely to develop IBS if they have an affected relative. Estimates of heritability from twin studies range widely from 0% up to 57%. Twin studies have indirectly investigated whether IBS and anxiety share a genetic basis, but have proved inconclusive.[1] Just one association has been previously reported at genome-wide significance in IBS. This was in a study of 11,621 cases. The association was identified in women with IBS subtype C and lacked robust replication.[2]

Description of cohorts

Description of the UK Biobank digestive health questionnaire

We compiled a 56-item questionnaire to assess digestive health, associated conditions and IBS risk factors in UK Biobank participants, titled the digestive health questionnaire (DHQ). The majority of the majority of content (>2/3rds of questions) consists of three validated instruments: the Rome III criteria used to diagnose IBS and categorise IBS patients into subtypes, the IBS Symptom Severity Score (IBS-SSS) designed to measure the severity of IBS symptoms (as detailed in the section "Calculation of IBS symptom severity score"), and the PHQ-12, designed to assess somatic symptoms in IBS patients (as detailed in the section "Calculation of PHQ-12 somatic symptom score").

The DHQ also includes a non-validated clinician-driven survey for identifying post-infectious IBS using standard diagnostic criteria, as well as the direct question “Have you ever been diagnosed with IBS?” (UK Biobank field 21024). Finally, the questionnaire asked a series of questions on correlates and risk factors for IBS, produced in consultation with clinician researchers in the field. These were based on self-reported recollection, and included the fields listed below:

Label (as in Table 1)	Prompt	UK Biobank field
family history of IBS	"Do you have a family history of IBS in your parents/siblings/children?"	21065
childhood antibiotics exposure	"During childhood or as a teenager did you receive long-term or recurrent courses (3 or more per year) of antibiotics (for example for tonsillitis or acne)?"	21067
born by caesarean	"Were you born by caesarian section?"	21066
treatment for anxiety offered or sought	"Have you ever been offered or sought treatment for anxiety?"	21062
treatment for depression offered or sought	"Have you ever been offered or sought treatment for depression?"	21063

The questionnaire was approved as a substantial amendment to the UK Biobank protocol by the North West - Haydock REC, reference 11/NW/038e.

The final DHQ (available online as UK Biobank resource 595) was incorporated into the UK Biobank Questionnaire platform and advertised via email as an online questionnaire entitled ‘digestive health’. Participants who had agreed to email contact were sent a hyperlink to their questionnaire, enabling linkage of results to other data in the UK Biobank dataset. The Digestive Health questionnaire was also available on the participant area of the UK Biobank website and participants without email addresses received a flyer with their annual postal newsletter encouraging them to login to the participants’ area of the UKB website, and complete the questionnaire. Prior to administration to participants with email addresses, the DHQ was piloted in 10,000 UK Biobank participants to assess acceptability. Following minor modifications this was presented online to a further 322,793 participants. Participants who did not respond or did not complete their questionnaire in full were sent reminders. The median time to complete the questionnaire was 8.5 min and 89.6% of participants completed the questionnaire in less than 20 minutes. Most DHQ data (>98%) used here was collected in 2017.

Definitions of IBS cases

IBS cases in Biobank were identified as meeting one or more of the following four criteria:

DHQ-based Rome III definition

Participants who completed the DHQ and whose abdominal symptoms were consistent with a diagnosis of IBS based on the Rome III case definitions given below, and who lacked other explanations for these symptoms (see exclusions below). This cohort is henceforth referred to as DHQ Rome III.

DHQ-based prompted self-report of previous IBS diagnosis

Participants who completed the DHQ and answered "Yes" to the question "Have you ever been diagnosed with IBS" (UK Biobank field 21024). This cohort is henceforth referred to as DHQ Self-report.

Unprompted self-report of previous IBS diagnosis

Participants who indicated a previous diagnosis of IBS without this being specifically prompted. At the Biobank recruitment visit (2006-2010), and in subsequent UK Biobank clinic follow-ups in 2012-2013 or 2014-2019, participants were asked via touch screen questionnaire about 'serious medical conditions previously diagnosed by a doctor'. If participants report the presence a serious medical condition, they are asked during a verbal interview with a nurse to state the condition(s) and if, in response to this, they indicated a previous diagnosis of IBS they were given diagnosis code 1154 ("irritable bowel syndrome") in the non-cancer illness field (UK Biobank field 20002). Participants with this diagnosis code in any of the three instances are henceforth referred to as Unprompted self-rep.

Hospital admission ICD-10 diagnosis

Participants diagnosed with IBS during a hospital admission and coded in the primary or secondary ICD-10 diagnosis as K58 (IBS) (UK Biobank fields 41202 and 41204). Data are linked to participants' UK Biobank records at the initial assessment or follow-up visit. This cohort is henceforth referred to as Hospital ICD-10.

We excluded cases whose symptoms met Rome III criteria but where HES data or medical history indicated a previous diagnosis with a potentially confounding condition that might also produce these symptoms. Excluded conditions included inflammatory bowel disease, GI malignancy, malabsorption, celiac or gluten sensitivity based on blood test or endoscopy, and a number of abdominal surgeries (see Supplementary Table 1 for the complete list). We did not exclude diverticular disease, dyspepsia, infectious gastroenteritis or gallbladder surgery due to common overlap or mis-specification of IBS into these diagnostic categories. We removed sample QC failures as described in "Sample QC" below.

Overlaps between the groups above were visualized using nVennR[3] and UpSetR[4].

For the functional constipation and functional diarrhea categories we applied the same exclusions as for DHQ Rome III and excluded any individuals previously diagnosed with IBS (as these were included in the IBS cohort).

Definition of IBS and IBS subtypes from DHQ data by Rome III classification criteria

We included questions from the Rome III IBS module[5] that allowed us to identify prevalent cases of IBS within UK Biobank from DHQ data. IBS cases met the following four criteria:

	Question	Positive answer	UK Biobank coding
Criteria 1	In the last 3 months, how often did you have discomfort or pain anywhere in your abdomen?	2-3 times per month or more	21025 in (3,4,5,6)
Criteria 2	For women: Did this discomfort or pain occur only during your menstrual bleeding and not at other times?	No or not applicable	21026 in (1,NA)
Criteria 3	Have you had this discomfort or pain 6 months or longer?	Yes	21027 = 1
Criteria 4: At least two of:	How often did this discomfort or pain get better or stop after you had a bowel movement?	At least sometimes	21028 in (-501,-502,-503,-504)
	When this discomfort or pain started, did you have more frequent bowel movements? OR When this discomfort or pain started, did you have less frequent bowel movements?	At least sometimes to at least one of these questions.	21029 in (-501, -502, -503, -504) OR 21030 in (-501, -502, -503, -504)
	When this discomfort or pain started, were your stools (bowel movements) looser? OR When this discomfort or pain started, were your stools (bowel movements) harder?	At least sometimes to at least one of these questions.	21031 in (-501, -502, -503, -504) OR 21032 in (-501, -502, -503, -504)

We defined IBS subtypes using standard Rome criteria on the basis of two DHQ questions: A) “In the last 3 months, how often did you have hard or lumpy stools?” (UK Biobank field 21033) and B) In the last 3 months, how often did you have loose, mushy or watery stools?” (UK Biobank field 21034). Constipation-predominant IBS (IBS-C) cases were defined as Rome III-positive IBS cases who answered at least “Sometimes” to the first question and “Never or rarely” to the second. Diarrhea-predominant IBS (IBS-D) cases were defined as Rome III-positive IBS cases who answer “Never or rarely” to A and at least “Sometimes” for B. Mixed IBS cases were defined as Rome III-positive IBS cases who answered at least “Sometimes” to both questions, and all other Rome-III positive cases were called unclassified IBS (IBS-U). Functional C and D subtypes were identified in the same way, but rather than being Rome-III positive, needed to have responded "Never" when asked about the frequency of abdominal pain in the last 3 months (UK Biobank field 21025).

Description of UK Biobank control cohorts

For the UK Biobank discovery cohort meta-analysis two control panels were identified according to DHQ response status (respondent or non-respondent) to match to respective case panels. The same exclusions were applied to both - namely exclusions as per the cases, but in addition those coded with diverticular disease, dyspepsia, infectious gastroenteritis or gallbladder surgery were excluded due to common overlap or mis-specification of IBS into these diagnostic categories, as well as IBS itself (Supplementary Table 2).

DHQ respondent controls were known to lack significant abdominal symptoms: they reported less than one day per month of abdominal pain; and hard/lumpy stools or loose/watery stools either ‘never’ or at most ‘sometimes’ in the last three months. For DHQ non-respondent controls we lacked this phenotype information and simply applied the exclusions as indicated above.

Description of Bellygenes cohorts

The *Bellygenes initiative* is a large international collaboration set up with the aim of identifying genes that affect IBS risk. It was originally conceived based on the exploitation of BBMRI resources and the bbmri-lpc cohorts (http://www.bbmri-lpc.org), and later expanded to include data from additional biobanks, population-based cohorts, and patient cohorts from tertiary IBS / neurogastroenterology clinics and expert IBS centers worldwide. Three independent datasets (and respective meta-analyses) from the *Bellygenes initiative* were included in this study (Supplementary Table 9), with the identification of IBS cases based on distinct definitions as outlined below. The Bellygenes initiative study received ethical approval from authorities at the Karolinska Institutet (ID 2016/1620-31/2) and Monash University (ID 20326).

Rome-based cohorts

The HUNT Study

The Nord-Trøndelag Health (HUNT) Study is a large population-based cohort in Norway including more than 125,000 Norwegian participants. Every citizen of Nord-Trøndelag County in Norway (>20 years old) has been invited to longitudinally health surveys and been followed-up in national health registers (PMID: 22978749). The HUNT Study has also collected biological samples (blood and urine) from participants. DNAs were extracted and genotyped using Illumina HumanCoreExome arrays. So far three health surveys have been completed in HUNT study. In HUNT3 survey (2006-2008), questions compatible with Rome III criteria have been included and are available for 14,894 unrelated participants. IBS patients in HUNT study were defined by Rome III criteria according to their answers in the HUNT3 surveys, and asymptomatic individuals from the remainder of the cohort were selected as controls. The demographics and GWAS pipeline of the HUNT study are reported in Supplementary Table 9. The study was approved by the Regional Committee for Medical and Health Research Ethics.

TWINGENE

The TWINGENE study includes epidemiological information from 45,750 Swedish twins (born in or before 1958) (PMID: 25248455). Participants were invited to telephone interviews between 1996 and 2002, during which GI symptoms were recorded by an adapted version of the Rome criteria, which allowed individuals to be classified as IBS patients. Controls were selected if they reported no bowel symptoms (negative answer to the question 'Ever had recurrent abdominal problems'). We further excluded individuals with reported diagnosis of IBD (both Crohn's disease and ulcerative colitis), celiac disease, peptic ulcer and GI cancer. As of 2012, GWAS genotyping data was available for 11,326 TWINGENE participants, the current GWAS has been performed on 5,154 independent singletons (504 IBS cases and 4650 asymptomatic controls). Singletons were selected from each individual twin pairs following an IBS-case-preferred algorithm. The demographics of TWINGENE samples and GWAS pipeline are reported in Supplementary Table 9. Informed consent was obtained from the study participants, and the study was approved by the Ethical Committee of the Karolinska Institutet.

Lifelines

Lifelines is a multi-disciplinary prospective population-based cohort study examining in a unique three-generation design the health and health-related behaviours of 167,729 persons living in the North of The Netherlands.[6] It employs a broad range of investigative procedures in assessing the biomedical, socio-demographic, behavioural, physical and psychological factors which contribute to the health and disease of the general population, with a special focus on multi-morbidity and complex genetics. Health questionnaire data, physical measurements and biological samples have been collected in Lifelines since 2006. IBS patients were identified in lifelines cohorts according to their answers to Rome III criteria-based questions being integrated into health questionnaires.

Controls were elected from the remainder of the cohort that had no sign of bowel symptoms. DNA samples from Lifelines participants were genotyped on Illumina CytoSNP-12v2 arrays, which allowed us to include 777 IBS patients and 7122 controls in the current GWAS. The demographics of Lifelines samples and GWAS pipeline are reported in Supplementary Table 9. The Ethics Committee of the University Medical Centre Groningen approved the study.

ICD-based cohorts

Mayo Genome Consortia

Mayo Genome Consortia (MayoGC) is a large cohort of Mayo Clinic patients in the USA with EMR and genotype data available for over 10,000 participants (PMID 21646302). Biological samples have been collected in the collaboration with Mayo Clinic Biobank, and genotype data is available for MayoGC participants. Participants were defined as IBS patients if they had any ICD9 code of IBS (564.1) in the EMR system. And controls were randomly selected from the remaining part of the cohorts, which allowed us to perform a GWAS on 342 IBS patients and 1,947 controls. The demographics of MayoGC samples and GWAS pipeline are reported in Supplementary Table 9. MayoGC Steering Committee has reviewed and approved this study.

Genetic Epidemiology Research on Aging

The Genetic Epidemiology Research on Aging (GERA) Cohort consists of more than 100,000 adults who are members of the Kaiser Permanente Medical Care Plan, Northern California Region (KPNC) of the USA. Health conditions of the GERA cohort participants were obtained from patient encounters at electronic medical records in KPNC facilities from January 1, 1995 to March 15, 2013. Biological samples have been collected from GERA participants and were genotyped on high-density custom-designed Affymetrix Axiom arrays. IBS patients were those who had at least two ICD9 code diagnoses of IBS (564.1), recorded on separate days. Their genetic data was compared to that of the remainder of the cohort, as controls. The demographics of GERA samples and GWAS pipeline are reported in Supplementary Table 9. The GERA data access has been applied for on the dbGaP website (https://www.ncbi.nlm.nih.gov/gap/, dbGaP Study Accession: phs000674.v2.p2) and the study has been approved by dbGap Access Review Committee.

Estonian Genome Center at the University of Tartu

The Estonian Biobank is a population-based biobank of the Estonian Genome Center at the University of Tartu (EGCUT), which comprises more than 52,000 participants over 18 years old (PMID: 24518929). At the recruitment, biological samples and health data were collected from GPs, other medical personnel in private practices and hospitals. A wide spectrum of '-omics' data, including genome-wide genotype data, are available for a significant part of the cohort. In this study, individuals with any record of an ICD10 code for IBS (K58) were included as cases, and we randomly selected study controls from the remainder of the cohort, which yielded 463 IBS patients and 2,244 controls in the current GWAS. The demographics of EGCUT samples and GWAS pipeline

are reported in Supplementary Table 9. This study has been reviewed and approved by the local ethics committee at the Estonian eHealth Foundation.

Michigan Genomics Initiative

The Michigan Genomics Initiative (MGI) is a longitudinal cohort of participants in Michigan Medicine, which has been described previously (PMID: 29779563). Briefly, MGI samples were recruited primarily through surgical encounters at Michigan Medicine and provided consent for linking of their EHRs and genetic data for research purposes. MGI samples were genotyped on customized Illumina HumanCoreExome v12.1 bead arrays. We have used a data freeze consisting of 40,000 European individuals for this analysis. Of these, 1,809 samples satisfied our criteria for being IBS cases (with any encounter of ICD10 code K58) and the remainder of the cohort (N=36,793) served as study controls. The demographics of MGI samples and GWAS pipeline are reported in Supplementary Table 9. This study has been reviewed and approved by the Michigan Institutional Review Board.

Tertiary center data

IBS patients of European ancestry were recruited from tertiary centers in Sweden (multi-center study), Italy (multi-center study), Belgium (TARGID Leuven), UK (Manchester), The Netherlands (Maastricht), Germany (IBSNet), Norway (Bergen) and the US (multi-center study). All these cohorts have been extensively characterized elsewhere (PMID: 24613995, 27974553, 26912503, 27151081, 24041540, 27725652, 26303129, 28107896, 25824902, 27263852, 29089619, 21911849). Ancestry-matched healthy controls were selected from previously published studies or general population cohorts. Swedish controls were blood donors being recruited at Örebro University Hospital (Örebro, Sweden). Italian healthy controls were blood donors previously being included in a study coordinated by the International IBD Genetics Consortium (IIBDGC). The healthy controls from the Stroke Genetics Network (SiGN) Study were used as controls in the Belgian dataset. UK controls were selected from a large general population cohort in the UK, Understanding Society, which integrates the health data of around 40,000 households. Dutch study controls consist of healthy individuals being recruited from the Maastricht area, and healthy volunteers in the 500 Functional Genomics Project. The healthy individuals from the PopGen Health study, a long-term population-based study in the North German population were chosen as German controls and we used the Nord-Trøndelag Health Study (HUNT), a large multiphase health study, as the Norwegian control data source. The healthy controls for the US were selected from a general population cohort, The Health and Retirement Study (HRS, based on the use of study data downloaded from the dbGaP web site, under phs000428.v2.p2) including over 12,000 Americans (PMID: 27263852, 21102463, 24021684, 29024973, 27814507, 16490960, 26950220, 24671021). The demographics of tertiary IBS samples and GWAS pipeline are reported in Supplementary Table 9. Informed consent of all IBS cases and controls was obtained locally at each center, and Karolinska Institutet's Research Ethics Committee approved the global study protocol.

Description of 23andMe cohort

The 23andMe cohort, used for replication, is described in Supplementary Table 20, and exclusively contains data from unrelated individuals of European descent. Based on data from multiple health questionnaires, participants indicating they had received a diagnosis of or treatment for IBS were defined as cases, and controls as participants who had not recorded an IBS diagnosis.

SNPs in the 23andMe dataset were imputed using Minimac3. An imputation quality filter with a minimum RSQR > 0.3 and an average RSQR > 0.5 was then applied. SNPs were additionally required to have p>1e-50 for a batch effect test, and genetic variance explained by sex, as a proportion of total genetic variance, < 0.01.

Test statistics from this cohort were generated by 23andMe using a linear model, and adjusted for an LDSC intercept of λ=1.149 (SE=0.011). Covariates included, age, sex, 4 genetic prinicipal components, as well as indicator variables for 23andMe genotyping platforms v2, v3_1, v4 and v5. Estimated heritability of IBS in the 23andMe cohort closely matched that in our discovery dataset at h^2=0.023 (SE=0.001).

Definition of post-infectious IBS

Evidence indicating a diagnosis of post-infectious IBS (PI-IBS) amongst DHQ respondents required onset of symptoms after an enteric infection. The latter was diagnosed either by a documented positive stool culture or acute onset of new bowel symptoms associated with two or more of fever, vomiting, diarrhea, rectal bleeding or onset during foreign travel.

Sample QC

Poorly genotyped samples, namely outliers in heterozygosity and missing rates were excluded via the "het.missing.outliers" and "excluded.from.kinship.inference" fields (see also section A6 of the UK Biobank Genotyping and Quality Control resource). The influence of familial relationships between samples was accounted for through our use of linear mixed models. To ensure our cases and controls shared similar ethnic backgrounds, the analysis was restricted to individuals with a British, Irish or any other white background as per UK Biobank field 21000. Individuals who did not know or preferred not to provide their ethnic background were also included, provided they were not genetic outliers: all samples were required to be within 7 standard deviations of the center defined across the first 6 principal components of the genetic data (UK Biobank field 22009). Analyses in non-white ethnicities were considered but not expected to produce reliable results in light of sample size limitations. Lastly, we removed any individuals who had withdrawn consent for use of their data since enrolment in UK Biobank. The total number of samples passing each of the filters above independently as well as cumulatively (N=436931) is shown in Supplementary Table 21.

Descriptive statistics

Calculation of IBS symptom severity score

We calculated an IBS symptom severity score (IBS-SSS) using a validated tool based on five subscores.[7] Three of the subscores (related to abdominal pain and distention) had a prompt question (to be answered yes or no), with “no” producing a zero score, and “yes” resulting in the user being given a 0 to 10 sliding scale to self-assess their severity for that subscore (see below). The other two subscores (related to bowel habit satisfaction and life interference) were scored via a sliding scale by all participants. The IBS severity score was taken as the total of the five subscores. The severity score was calculated across all 171061 individuals who answered the 5 questions, regardless of their IBS diagnosis. We divided participants with an IBS diagnosis into groups with mild (<175), moderate (175-300) and severe (>300) symptoms based on the IBS-SSS. The subscores were as follows:

	Prompt	Scoring	UK Biobank coding
Subscore 1	Do you currently (in the last three months) suffer from abdominal (tummy) pain?	How severe is your abdominal pain? 0 meaning “no pain” and 10 meaning “severe pain”?	Prompt 21035, score 21036
Subscore 2	Do you currently (in the last three months) suffer from abdominal (tummy) pain?	Select the number of times you get the pain every 10 days.	Prompt 21035, score 21037
Subscore 3	Do you currently suffer from abdominal distention (bloating, swollen or tight tummy)?	How severe is your abdominal distention/tightness/? 0 meaning “no distention” and 10 meaning “very severe”?	Prompt 21038, score 21039
Subscore 4	No prompt (all participants give a score)	How happy/satisfied are you with your bowel habits? With 0 meaning “very happy” and 10 meaning “very unhappy”.	Score 21040
Subscore 5	No prompt (all participants give a score)	Please indicate how much abdominal pain or discomfort or altered bowel habits are affecting or interfering with your life in general. 0 meaning "not at all" and 10 meaning "completely".	Score 21041

Calculation of PHQ-12 somatic symptom score

We used the PHQ-12 score[8] to assess the overall degree of non-digestive somatic symptoms. The PHQ-12 is a modification of the PHQ-15 score[9], and is specifically designed for patients with digestive complaints by excluding questions that directly apply to measures of digestive distress. Both the PHQ-12 and PHQ-15 are designed to measure somatic (i.e. non-psychiatric) symptoms via patient self-assessment.

Each participant is asked "During the past 3 months, how much have you been bothered by any of the following problems?", followed by a list of 12 somatic complaints, and are asked to pick from three options: "Not bothered at all". "Bothered a little" and "Bothered a lot". The PHQ-12 score increases by 1 for each "Bothered a little" answer, and 2 for each "Bothered a lot" answer, producing a score between 0 and 24. The symptoms are given below:

Symptom	Label	UK Biobank field
Back pain	Back pain	21048
Headaches	Headache	21051
Chest pain	Chest pain	21052
Dizziness	Dizziness	21053
Fainting spells	Fainting	21054
Feeling your heart pound or race	Palpitations	21055
Shortness of breath	Breathless	21056
Pain or problems during sexual intercourse (see below)	Dyspareunia	21057
Pain in your arms, legs, or joints (knees, hips, etc)	Limb pain	21049
Feeling tired or having low energy	Tired	21060
Menstrual cramps or other problems with your periods (see below)	Period	21050
Trouble sleeping	Sleep disorder	21060

Our version of the PHQ-12 score did not include menstrual symptoms, in order to not bias the score by sex, as per Polster et al.,[10] and was therefore limited to a maximum score of 22. We only report PHQ-12 scores among individuals for whom all of the included symptom scores were available, noting that "Pain or problems during sexual intercourse" was scored as 0 when participants responded "Not applicable".

Calculation of GAD-7 anxiety score

To assess symptoms of generalized anxiety disorder (GAD), we used the GAD-7 score.[11] UK Biobank participants answering the "Thoughts and Feelings" mental health questionnaire were asked "Over the last 2 weeks, how often have you been bothered by any of the following problems?", and could respond "Not at all", "Several days", "More than half of the days", "Nearly every day", or "Prefer not to answer". The first four responses were worth 0, 1, 2 and 3 points, respectively. The final GAD-7 score is produced by summing these points across all symptoms, listed below:

Symptom	Label	UK Biobank field
Feeling nervous	Nervous	20506
Not being able to stop or control worrying	Uncontrollable worry	20509
Worrying too much about different things	Excess worry	20520
Trouble relaxing	Trouble relaxing	20515
Being so restless that it is hard to sit still	Restless	20516
Becoming easily annoyed or irritable	Irritable	20505
Feeling afraid as if something awful might happen	Foreboding	20512

We only report GAD-7 scores among individuals for whom all of the included symptom scores were available.

Calculation of PHQ-9 depression score

To quantify symptoms of depression, we calculated PHQ-9 scores[12] amongst UK Biobank participants responding to the "Thoughts and Feelings" mental health questionnaire, administered as in the GAD-7 section above, with the same introductory statement, response options and score weighting. PHQ-9 items are listed below:

Symptom	Label	UK Biobank field
Little interest or pleasure in doing things	Anhedonia	20514
Feeling down	Feeling down	20510
Trouble falling or staying asleep	Sleep disorder	20517
Feeling tired or having little energy	Tired	20519
Poor appetite or overeating	Appetite disorder	20511
Feeling bad about yourself or that you are a failure or have let yourself or your family down	Feeling inadequate	20507
Trouble concentrating on things	Concentration	20508
Moving or speaking so slowly that other people could have noticed? Or the opposite — being so fidgety or restless that you have been moving around a lot more than usual	Tach- or bradykinetic	20518
Thoughts that you would be better off dead or of hurting yourself in some way	Self-harm	20513

We only report PHQ-9 scores among individuals for whom all of the included symptom scores were available.

Median scores among pooled and individual diagnoses

From Supplementary Table 4 and Supplementary Table 6, it may seem counterintuitive that the median of several sets of scores we combine (for the pooled IBS diagnosis) is more extreme than the median observed for any set of scores individually.

However, if we take all scores in set A in sequence, and add to this sequence the scores of set B, we should skip scores we have already observed, such that we are not counting some participants' scores twice in the pooled group.

Consider 4 unique participants with scores 0, 1, 8 and 9. The participants with scores 8 and 9 are members of multiple diagnostic groups (A and B) simultaneously:

Scores for group A: {1,8,9}, median 8

Scores for group B: {0,8,9}, median 8

Scores for the pool of A and B: {0,1,8,9}, median 4.5

Now the median score after pooling is lower than it was for either set individually.

The key is that we would not observe a more extreme median if we naively took {0,1,8,8,9,9} as the pooled set of scores. That is what you may expect the pool to look like at first glance, and it still has a median of 8, but it mistakenly has the patients with scores 8 and 9 appearing twice.

In practice, participants who are in multiple diagnostic groups tend to have higher IBS-SSS/PHQ-12 scores, and so it is predominantly high scores that end up less represented in the combined set than you might expect. The remaining (lower) scores then decrease the median score in the pooled group, as is seen.

Association analyses

Controlling for response bias

There are systematic differences between respondents and non-respondents. Respondents have lower rates of IBS as measured via hospital ICD-10 codes (1.16% vs 1.40% among 171,061 respondents and 317,234 non-respondents, respectively), but not unprompted self-reporting (2.85% vs 2.30%). They also have lower rates of mental health disorders based on hospital ICD-10 codes (schizophrenia: 0.04% vs 0.21%, depression: 1.77% vs 3.40%) and unprompted self-reporting (schizophrenia: 0.05% vs 0.16%, depression: 5.83% vs 6.20%). Respondents also had a lower mean age than non-respondents (64.8 vs 65.8 years when the DHQ data were collected), and were more often female (56.7% vs 52.9%). Response rates also varied by ethnicity, e.g. 15.8% (1205 of 7645) among participants who report a Black or Black British background compared to 36.0% (165243 of 459256) among participants reporting a White ethnic background. We show that responder effects can also cause artifactual differences in genetic signals across analyses if not controlled for (Supplementary Fig. 14). In our genetic association tests, we therefore analyzed DHQ respondents and non-respondents separately, and then meta-analyze the results to eliminate the confounding effect of DHQ response on IBS risk. In non-genetic analysis, e.g. between IBS and clinical risk factors, we control for DHQ response status by adding it as a covariate to our logistic regression models, along with factors such as age and gender.

Non-genetic associations

We tested the association between different IBS diagnoses and a variety of risk factors and comorbidities. These included the outcomes of the risk factor questions from the DHQ (as described in the section "Description of the UK Biobank digestive health questionnaire"), as well as anxiety-related diagnoses (based on the DHQ, unprompted self-report data and hospital ICD-10 codes) and anxiety (GAD-7) and depression (PHQ-9) symptom scores. We also tested for the pooled

definition of atopy (asthma, eczema and hayfever, based on unprompted self-report and hospital ICD-10 codes).

Associations between binary conditions (e.g. IBS case or control status) and non-genetic variables, such as IBS family history, use of childhood antibiotics, birth by caesarean section, IBS-SSS, PHQ-12, PHQ-9 or GAD-7 scores, but also anxiety or depression treatment, were carried out using logistic regression unless otherwise stated. To account for age and gender differences between the two groups being compared, these factors, their transformations and interactions (sex, age, age^2, sex*age, sex*age^2), were added into the model as covariates. Fundamental differences between DHQ respondents and non-respondents were accounted for by adding DHQ response status as an additional covariate. For each analysis, we ultimately report the odds ratio and 95% confidence interval for the explanatory variable (e.g. IBS-SSS). For differences in the association between the predictor for two outcomes (e.g. IBS and functional diarrhea) we calculate the excess odds ratio of the first outcome relative to the second (i.e. the ratio of the odds for the first outcome and the second outcome). For all analyses, only individuals without missing data were analysed.

Genetic association testing and SNP filtering

All genetic association testing was performed using linear mixed models as implemented in BOLT-LMM[13] version 2.3.2. The genetic relationship matrix was derived from 622502 genotyped autosomal SNPs with a minor allele frequency of 0.01 or greater and a genotyping rate of 90% or greater, extracted using PLINK[14] version 1.90b3. We calculated test statistics for a total of 10239733 genotyped and imputed SNPs across the autosomes and the X chromosome, with a minimum allele frequency of 0.01 and, for imputed SNPs, a minimum INFO score of 0.3. Covariates accounted for were 0-mean definitions of sex, age, and sex*age, as well as age^2 and $(sex*age)^2$, along with the first 20 principal components of the genetic data available through UK Biobank field 22009. Association testing was only carried out on individuals with non-missing phenotype data.

Meta-analysis

Association statistics from UK Biobank (from 40,548 cases, 360,845 controls) were combined with those from independent Bellygenes initiative cohorts (12,852 cases, 139,981 controls) using the meta-analysis software METAL[15] based on positional information. Prior to the meta-analysis, association statistics from all datasets were converted to the log odds ratio scale, dividing BOLT-LMM case-control effect sizes and standard errors by m*(1-m), where m is the proportion of cases in the analysis.

Clumping

To differentiate independently associated SNPs, we clumped correlated variants together. All variants to be clumped were required to be well imputed, having a minimum INFO score of 0.9, and a minimum allele frequency of 0.0001 or greater. Such variants were extracted from UK Biobank's

v3 imputation, in bgen format, and converted to bed format using PLINK version 2.00a2. We followed a conservative approach while clumping with PLINK version 1.90b6.7, favoring a few large clumps, rather than many small ones. Lead SNPs were required to be genome-wide significant at $p<5e-8$, and any nominally significant SNPs within 5 Mbp and a minimum r^2 of 0.05 with a lead SNP were added to their clump. LD was calculated in a subset of 10,000 randomly selected QC-positive UK BioBank samples.

Conditional analysis

We extracted sets of all SNPs between the variants marking the boundaries of each clump, and used gcta-select in GCTA[16] 1.92.0 beta 1 to select independently associated SNPs, and to uncover potential signals attenuated via high-LD SNPs with opposite effect sizes. We used 10,000 unrelated individuals passing genetic QC in UK Biobank as a reference LD panel. The lead SNP in each clump best represented the signal in each case, and no additional signals were identified.

We additionally investigated whether the observed influence of HLA allele B*0801 (UK Biobank HLA imputation coding “B_801”) and HLA region SNP rs2736155 on IBS might stem from shared genetic architecture with gastrointestinal disorders such as ulcerative colitis, microscopic colitis and celiac disease, which are known to be influenced by variants in the HLA region. We extracted SNPs associated with gastrointestinal disorders other than IBS (Supplementary Table 11) from UK Biobank bgen data in expected dosage format using qctool 2.0.5 (https://www.well.ox.ac.uk/~gav/qctool_v2/), and simultaneously added all of these as covariates in our BOLT-LMM analysis, separately for each trait. In the case of celiac disease, the expected dosage values of HLA alleles DQ8 (UK Biobank HLA imputation coding "DQA1_301" and "DQB1_302") and DQ2.5 (UK Biobank HLA imputation coding "DQA1_501" and "DQB1_201") were also both used as covariates in a separate analysis. Whether celiac disease-associated HLA alleles or their proxy SNPs were used did not influence the results of the conditional analysis.

Replication

Lead SNPs and two proxies for each independent association from each analysis were sent to 23andMe for replication in a cohort with 205,252 self-reported IBS cases and 1,384,055 controls. Across analyses, we sent 20 independent associations for replication (min. 250kb apart, max. r^2 of 0.2), which included 6 loci from the discovery analysis, 8 loci from the additional key definitions shown in Supplementary Fig. 8, 1 from the severe IBS analysis, and 5 from GWAS of intermediate traits used in the meta-analysis and methodological variations.

All but two of variants submitted for replication, marked with an asterisk in Supplementary Table 13, were matched in the 23andMe dataset. The variants missing from 23andMe data (6:31610189_TAAAG_T, rs746685195) both had proxies with $r^2 \geq 0.965$ in our dataset which were matched.

Associations were considered replicated if SNPs had identical directions of effect in both datasets, were significant in the 23andMe data ($p_{23andMe}$<0.05/N where N=20 associations), and remained genome-wide significant following meta-analysis (p_{meta}<5e-8).

Fine mapping

Fine-mapping was performed using the approximate bayes factor method implemented in the Coloc[17] R package, with a prior probability of association p1=1e-04. We repeated this procedure for all 200kb regions centered around the lead SNPs identified via clumping, under the assumption that each harbored a single causal variant.

HLA association testing

We used the imputed HLA dosages from the v2 UK Biobank release (see UK Biobank field 22182 and Resource 182) to identify HLA allele associations with BOLT-LMM, treating each HLA allele as if it were a SNP (using dummy values for any allele fields). HLA alleles with a frequency below 0.01 were skipped. We used the same covariates and same genetic relationship matrix as for the autosomal analyses.

Functional interpretation of associations

Gene mapping

To map IBS-associated SNPs to genes, we used the positional, chromatin-structure, and eQTL mapping methods implemented in FUMA,[18] as well as MAGMA. For positional mapping, we used pre-defined lead SNPs identified through clumping (above) and proxies in high LD and linked these to genes within 10kb. eQTL mapping was based on exact overlap between the same SNPs and those known to influence gene expression in 12 available datasets (listed online at https://fuma.ctglab.nl/tutorial#eQTLs). For chromatin mapping, all Hi-C datasets available through the platform were used.

We additionally looked for colocalization between our signal for IBS risk and that for changes in the expression of genes in the GTEx v7 dataset.[19] Specifically, we used the Coloc[17] R package within any 2Mbp window of expression data (the maximum range for which expression data is available around genes in GTEx v7) overlapping our hits, with a posterior probability of colocalization > 0.5 implicating the gene. All genes implicated via at least one of these methods are listed in Supplementary Table 10. Those with multiple lines of support were selected for manual review.

Gene expression enrichment

Differential expression scores were computed with Expression Weighted Celltype Enrichment[20] and alternatively with a t-statistic, as described in previous enrichment-testing literature.[21] For the discrete analyses, a top 10% gene score threshold was used. Enrichment testing was performed with LDSC, SNPsea and MAGMA with all the default parameters mentioned in the package papers.[20–23] For multiple comparisons correction, the Benjamini Hochberg procedure was used.

Comparison to previous GWAS

GWAS Catalog

GWAS Catalog[24] results were accessed via FUMA, to which lead SNPs identified via clumping were provided as pre-determined index SNPs. 3 IBS loci, consisting of these index SNPs and any proxies in high LD (r^2>0.8) with them, were matched to genome-wide significant associations in publications previously submitted to GWAS catalog. Alleles were flipped in order to report GWAS catalogue effect sizes and directions relative to the IBS risk allele, with allele frequency used to check the consistency of the alleles (Supplementary Table 22). These were manually reviewed, but not found to contain additional functional interpretations of the implicated genes. Functional insights were subsequently derived following additional literature searches.

Genetic correlation estimates

Genetic correlations between IBS phenotypes and other traits were calculated using LDHUB.[25] Data on all genetic correlation estimates is available only via figshare at https://figshare.com/s/6c7e6717a775196a6440. The initial selection of LDHub disease traits for which genetic correlations are shown in Fig. 3 requires p_{rg}<0.05 in the UK Biobank data, the additional cohorts, and the meta-analysis of these data. Rapid GWAS results (http://www.nealelab.is/uk-biobank) were excluded from this part of the analysis. Where multiple GWAS were published for the same trait, we show data from the GWAS with which the genetic correlation estimate had the smallest standard error, which generally favors more recent GWASes with larger sample sizes. We additionally included data on traits that were defined to be of interest a priori, marked in yellow in Fig. 3. We note that the anxiety/panic attack rapid GWAS we selected has not been peer reviewed.

Genetic correlations between IBS definitions were calculated using LDSC[26] directly. We ensured sample overlap between IBS definitions did not inflate estimates by conducting two GWASes for each definition in randomly partitioned, separate halves of the UK Biobank. Results continued to show high genetic correlations between definitions (median pairwise r_g = 0.90, median SE=0.13).

Phenotypic correlation estimates

To investigate the phenotypic overlap between IBS and the traits for which genetic correlations are shown in Fig. 3, we identified matching self-reported (UK Biobank field 20002) and ICD-10 codes (UK Biobank fields 41202, 41204) in the UK Biobank for each trait. UK Biobank participants flagged by any of these codes were considered cases, and all remaining participants were considered controls. For IBS, the same case and control definitions were identical to those in the GWAS. Odds ratios were calculated using logistic regression for respondents and non-respondents separately, while accounting for the same covariates as in the GWAS (aside from principal components), and subsequently meta-analyzed. Following the observation that sex and DHQ response influenced phenotypic overlap with IBS, we calculated phenotypic correlations using a liability model based on contigency tables for non-respondent males, non-respondent females, respondent males and respondent females, and then meta-analyzed the results. In the case of neuroticism, we used the neuroticism score as a continuous trait in the liability model.

Mendelian randomization analyses

We used unidirectional Mendelian randomization (inverse-variance weighted, IVW) and bidirectional Mendelian randomization (MR-Steiger),[27] implemented in the R package TwoSampleMR[28] (https://github.com/MRCIEU/TwoSampleMR) to test for evidence of causal effects of anxiety on IBS , using data from an orthogonal study of anxiety (measured via the GAD-2) in the Million Veterans Program.[29] We also carried out both MR analyses on all significant phenotypes from Fig. 3 with non-UK Biobank summary statistics publicly available. The PubMed IDs of the publication that the non-IBS summary statistics were taken from are shown in Supplementary Table 19 (only non-UK Biobank summary statistics were used to avoid sample overlap). When IBS was the exposure, the six discovery loci were used, and when IBS was the outcome, all independent genome-wide significant associations reported in the corresponding paper were used.

We also used our IBS discovery summary statistics and UK Biobank anxiety summary statistics to fit a latent hidden variable model using LHC-MR,[30] using default parameters for UK Biobank analyses. Models were compared using likelihood ratio tests for embedded models and the Akaike information criterion (AIC) for non-embedded models.

References

1. Bengtson, M.-B., Aamodt, G., Vatn, M. H. & Harris, J. R. Co-occurrence of IBS and symptoms of anxiety or depression, among Norwegian twins, is influenced by both heredity and intrauterine growth. *BMC Gastroenterol.* **15**, 9 (2015).
2. Bonfiglio, F. *et al.* Female-Specific Association Between Variants on Chromosome 9 and Self-Reported Diagnosis of Irritable Bowel Syndrome. *Gastroenterology* **155**, 168–179 (2018).
3. Pérez-Silva, J. G., Araujo-Voces, M. & Quesada, V. nVenn: generalized, quasi-proportional Venn and Euler diagrams. *Bioinformatics* **34**, 2322–2324 (2018).
4. Conway, J. R., Lex, A. & Gehlenborg, N. UpSetR: an R package for the visualization of intersecting sets and their properties. *Bioinformatics* **33**, 2938–2940 (2017).
5. Longstreth, G. F. *et al.* Functional bowel disorders. *Gastroenterology* **130**, 1480–1491 (2006).
6. Scholtens, S. *et al.* Cohort Profile: LifeLines, a three-generation cohort study and biobank. *Int. J. Epidemiol.* **44**, 1172–1180 (2015).
7. Francis, C. Y., Morris, J. & Whorwell, P. J. The irritable bowel severity scoring system: a simple method of monitoring irritable bowel syndrome and its progress. *Aliment. Pharmacol. Ther.* **11**, 395–402 (1997).
8. Spiller, R. C. *et al.* The Patient Health Questionnaire 12 Somatic Symptom scale as a predictor of symptom severity and consulting behaviour in patients with irritable bowel syndrome and symptomatic diverticular disease. *Aliment. Pharmacol. Ther.* **32**, 811–820 (2010).
9. Kroenke, K., Spitzer, R. L. & Williams, J. B. W. The PHQ-15: validity of a new measure for evaluating the severity of somatic symptoms. *Psychosom. Med.* **64**, 258–266 (2002).
10. Polster, A. V. *et al.* Subgroups of IBS patients are characterized by specific, reproducible profiles of GI and non-GI symptoms and report differences in healthcare utilization: A population-based

study. *Neurogastroenterology & Motility* **31**, e13483 (2019).

11. Spitzer, R. L., Kroenke, K., Williams, J. B. W. & Löwe, B. A brief measure for assessing generalized anxiety disorder: the GAD-7. *Arch. Intern. Med.* **166**, 1092–1097 (2006).
12. Kroenke, K. & Spitzer, R. L. The PHQ-9: A New Depression Diagnostic and Severity Measure. *Psychiatr. Ann.* **32**, 509–515 (2002).
13. Loh, P.-R., Kichaev, G., Gazal, S., Schoech, A. P. & Price, A. L. Mixed-model association for biobank-scale datasets. *Nat. Genet.* **50**, 906–908 (2018).
14. Purcell, S. *et al.* PLINK: a tool set for whole-genome association and population-based linkage analyses. *Am. J. Hum. Genet.* **81**, 559–575 (2007).
15. Willer, C. J., Li, Y. & Abecasis, G. R. METAL: fast and efficient meta-analysis of genomewide association scans. *Bioinformatics* **26**, 2190–2191 (2010).
16. Yang, J., Lee, S. H., Goddard, M. E. & Visscher, P. M. GCTA: a tool for genome-wide complex trait analysis. *Am. J. Hum. Genet.* **88**, 76–82 (2011).
17. Giambartolomei, C. *et al.* Bayesian test for colocalisation between pairs of genetic association studies using summary statistics. *PLoS Genet.* **10**, e1004383 (2014).
18. Watanabe, K., Taskesen, E., van Bochoven, A. & Posthuma, D. Functional mapping and annotation of genetic associations with FUMA. *Nat. Commun.* **8**, 1826 (2017).
19. GTEx Consortium. The Genotype-Tissue Expression (GTEx) project. *Nat. Genet.* **45**, 580–585 (2013).
20. Skene, N. G. & Grant, S. G. N. Identification of Vulnerable Cell Types in Major Brain Disorders Using Single Cell Transcriptomes and Expression Weighted Cell Type Enrichment. *Front. Neurosci.* **10**, 16 (2016).
21. Finucane, H. K. *et al.* Heritability enrichment of specifically expressed genes identifies disease-relevant tissues and cell types. *Nat. Genet.* **50**, 621–629 (2018).

22. de Leeuw, C. A., Mooij, J. M., Heskes, T. & Posthuma, D. MAGMA: generalized gene-set analysis of GWAS data. *PLoS Comput. Biol.* **11**, e1004219 (2015).

23. Slowikowski, K., Hu, X. & Raychaudhuri, S. SNPsea: an algorithm to identify cell types, tissues and pathways affected by risk loci. *Bioinformatics* **30**, 2496–2497 (2014).

24. Buniello, A. *et al.* The NHGRI-EBI GWAS Catalog of published genome-wide association studies, targeted arrays and summary statistics 2019. *Nucleic Acids Res.* **47**, D1005–D1012 (2019).

25. Zheng, J. *et al.* LD Hub: a centralized database and web interface to perform LD score regression that maximizes the potential of summary level GWAS data for SNP heritability and genetic correlation analysis. *Bioinformatics* **33**, 272–279 (2017).

26. Bulik-Sullivan, B. K. *et al.* LD Score regression distinguishes confounding from polygenicity in genome-wide association studies. *Nat. Genet.* **47**, 291–295 (2015).

27. Hemani, G., Tilling, K. & Davey Smith, G. Orienting the causal relationship between imprecisely measured traits using GWAS summary data. *PLoS Genet.* **13**, e1007081 (2017).

28. Hemani, G. *et al.* The MR-Base platform supports systematic causal inference across the human phenome. *Elife* **7**, (2018).

29. Levey, D. F. *et al.* Reproducible Genetic Risk Loci for Anxiety: Results From ∼200,000 Participants in the Million Veteran Program. *Am. J. Psychiatry* **177**, 223–232 (2020).

30. Darrous, L., Mounier, N. & Kutalik, Z. Simultaneous estimation of bi-directional causal effects and heritable confounding from GWAS summary statistics. *Genetic and Genomic Medicine* (2020) doi:10.1101/2020.01.27.20018929.

Supplementary figures

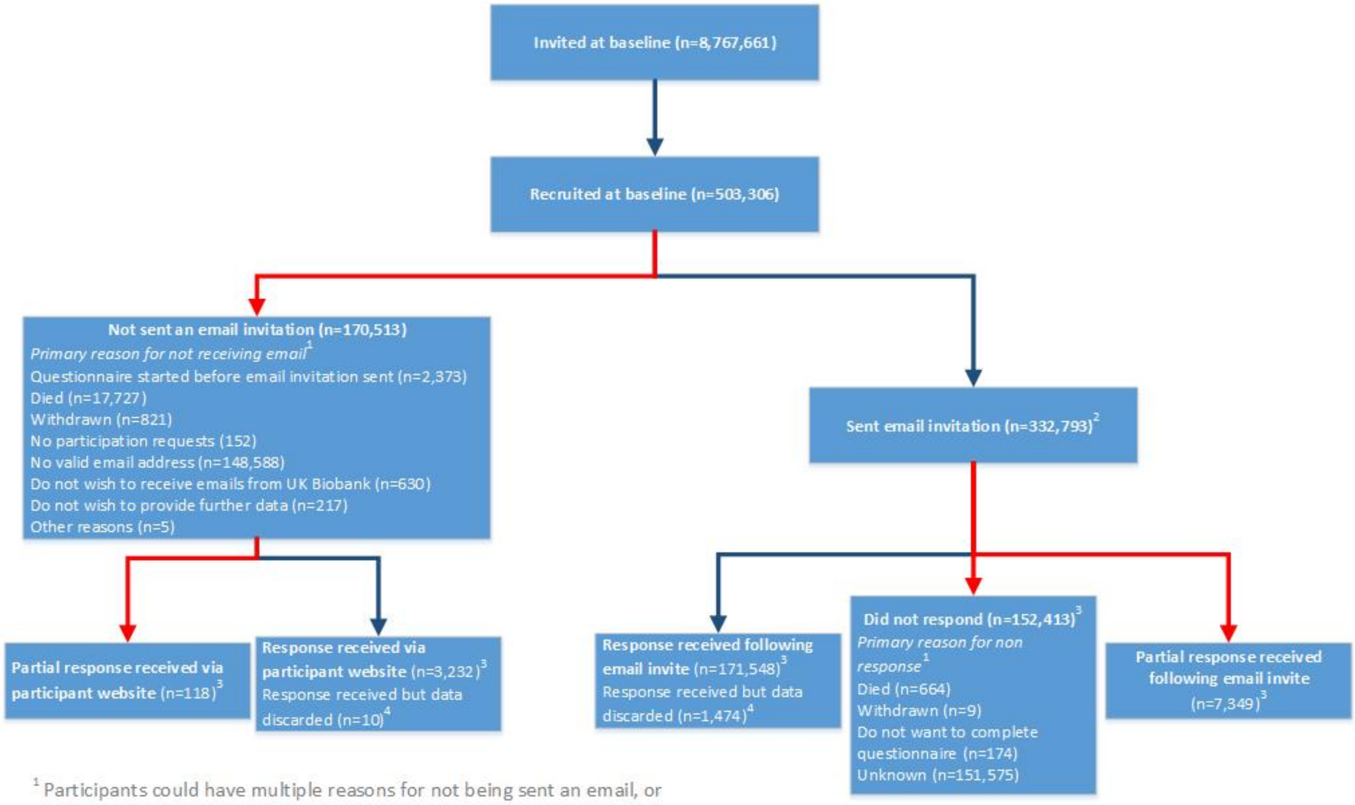

[1] Participants could have multiple reasons for not being sent an email, or for not responding. For the purposes of this flowchart, we have identified the most important reason why people were not sent an email.
[2] Emails were sent up to and including 18th July 2018. Email invites have been sent since this date but any resultant data are not included here.
[3] Cut off date for providing responses was 18th July 2018.
[4] Data were discarded where personal details entered did not match those of the participant.

Supplementary Figure 1: Flowchart of UK Biobank participants from invitation to completion of the DHQ. Invitations were based on NHS registration, Age and location. Numbers correct for July 2018. At the time of our data cut for analysis 171,061 complete responses had been received (487 arrived after this cut).

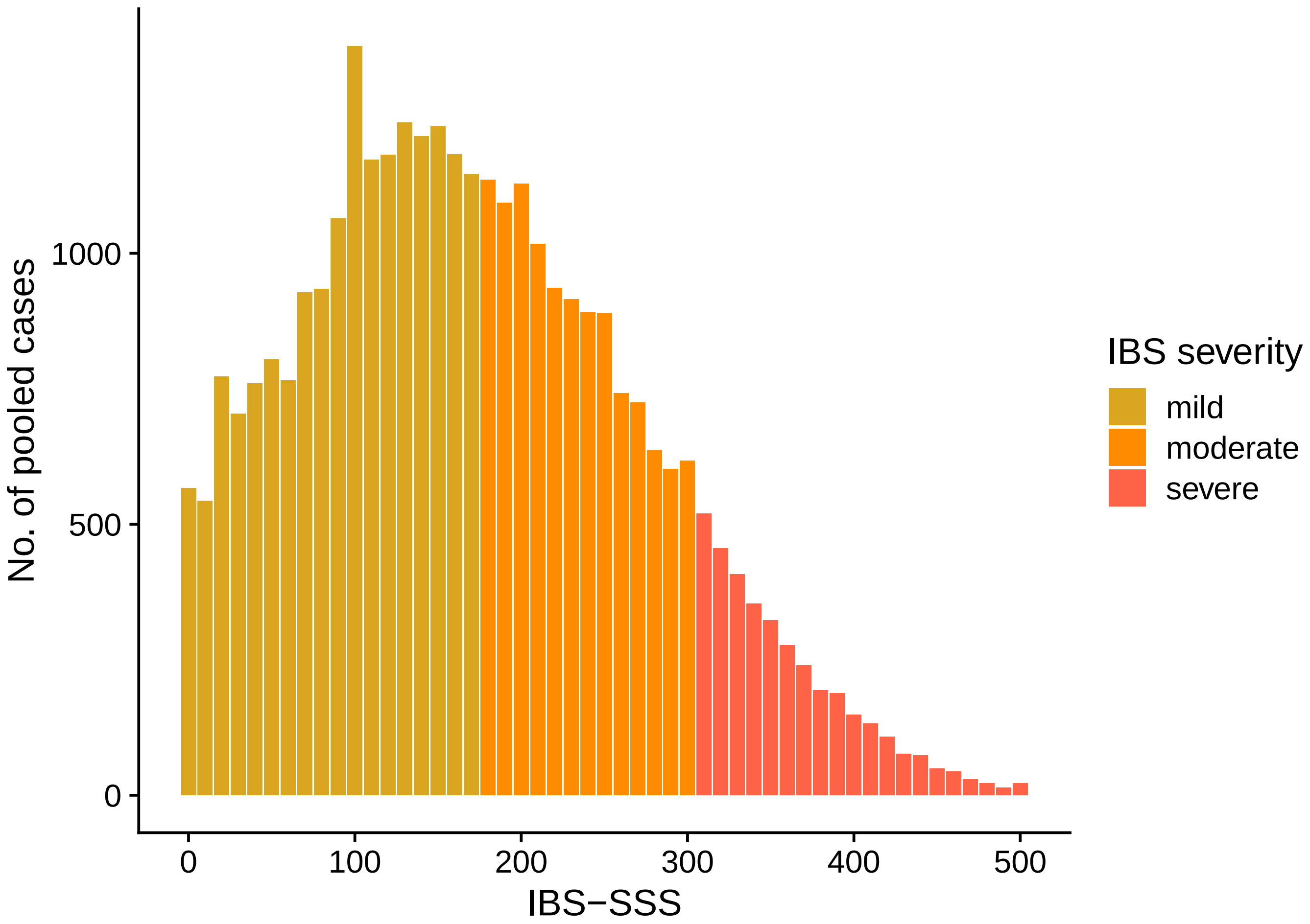

Supplementary Figure 2: Distribution of gastrointestinal symptom severity amongst the IBS cases of the discovery cohort who completed the IBS symptom severity score (IBS-SSS) questions in the DHQ.

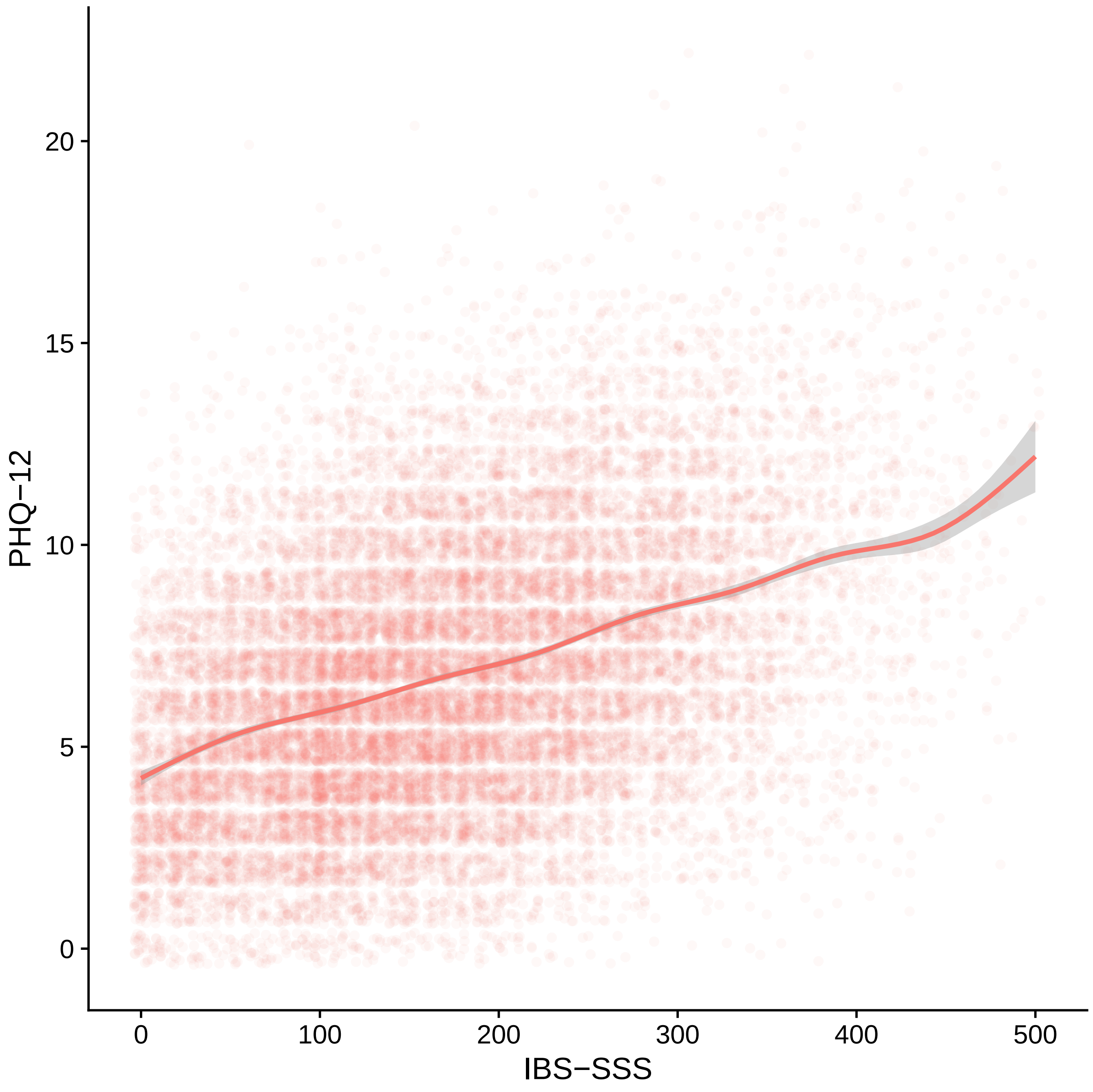

Supplementary Figure 3: Correlation between gastrointestinal symptoms (as assessed by the IBS-SSS) and somatic symptoms (PHQ-12 score) among IBS cases. Jittered points for 10,000 randomly sampled cases are shown, along with smoothed conditional means (banded by a 95% CI) for all cases. Pearson's correlation was 0.40 [95% CI: 0.39 - 0.41] among 31,402 IBS cases completing all PHQ-12 SS and IBS-SSS questions.

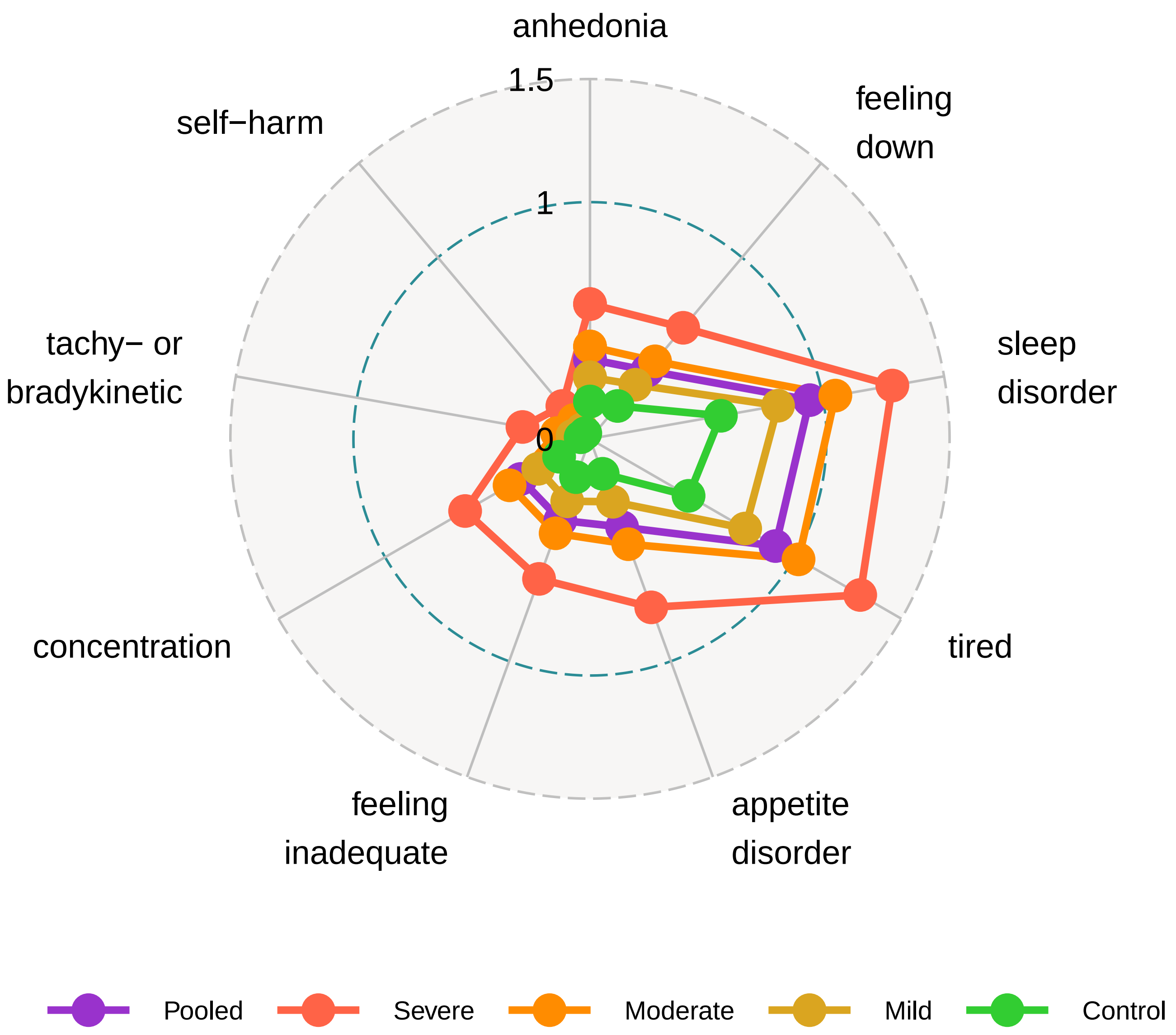

Supplementary Figure 4: Symptoms of depression are worse in individuals with more severe IBS symptoms (classified as mild, moderate, severe based on IBS-SSS). Mean scores for PHQ-9 items (ranked from 0=not bothered at all to 3=bothered a lot) are shown.

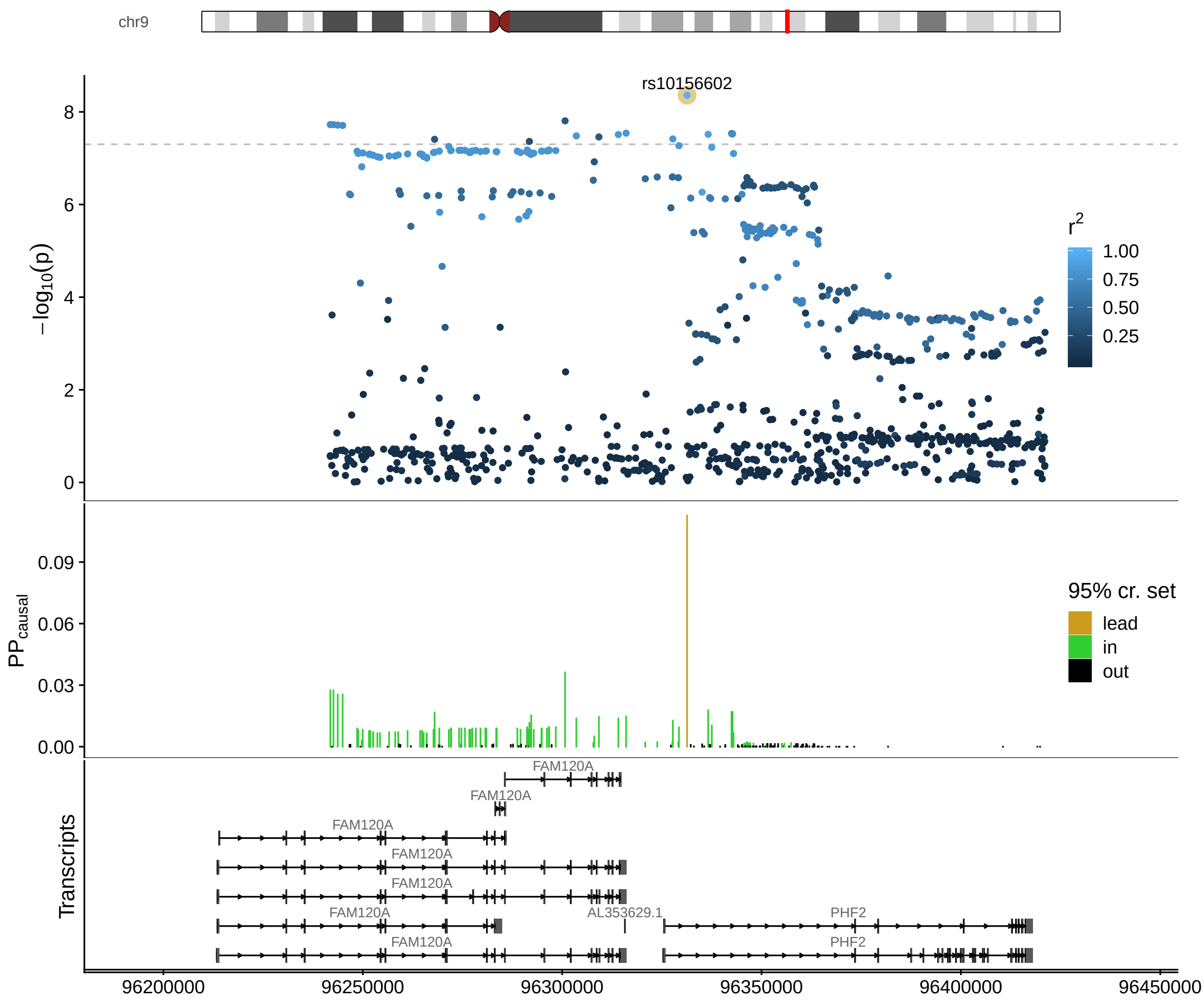

(a) rs10156602

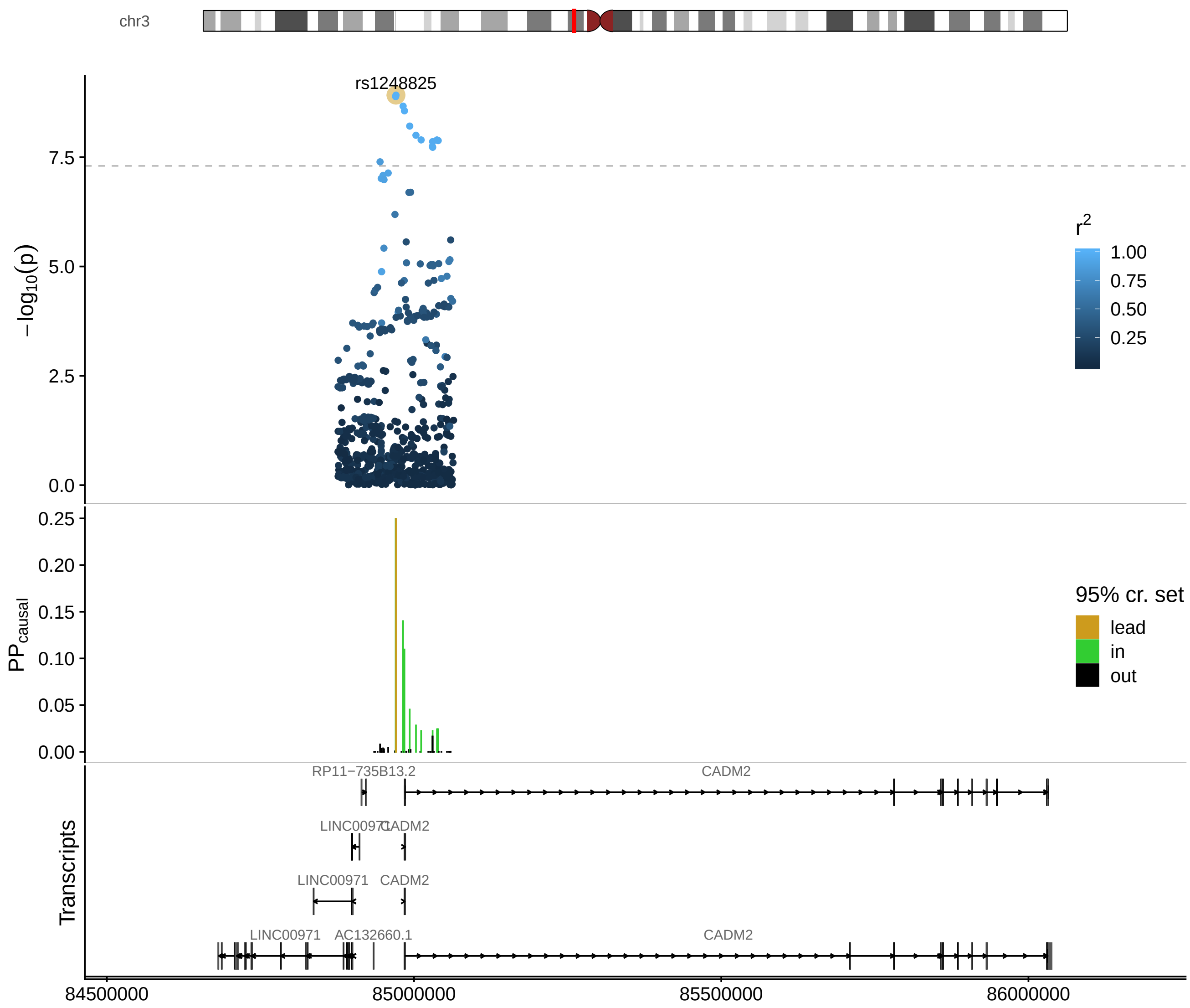

chr3
rs1248825
$-\log_{10}(p)$
7.5
5.0
2.5
0.0
r^2
1.00
0.75
0.50
0.25
PP_{causal}
0.25
0.20
0.15
0.10
0.05
0.00
95% cr. set
lead
in
out
Transcripts
RP11−735B13.2
CADM2
LINC00971
CADM2
LINC00971
CADM2
LINC00971
AC132660.1
CADM2
84500000
85000000
85500000
86000000

(b) rs1248825

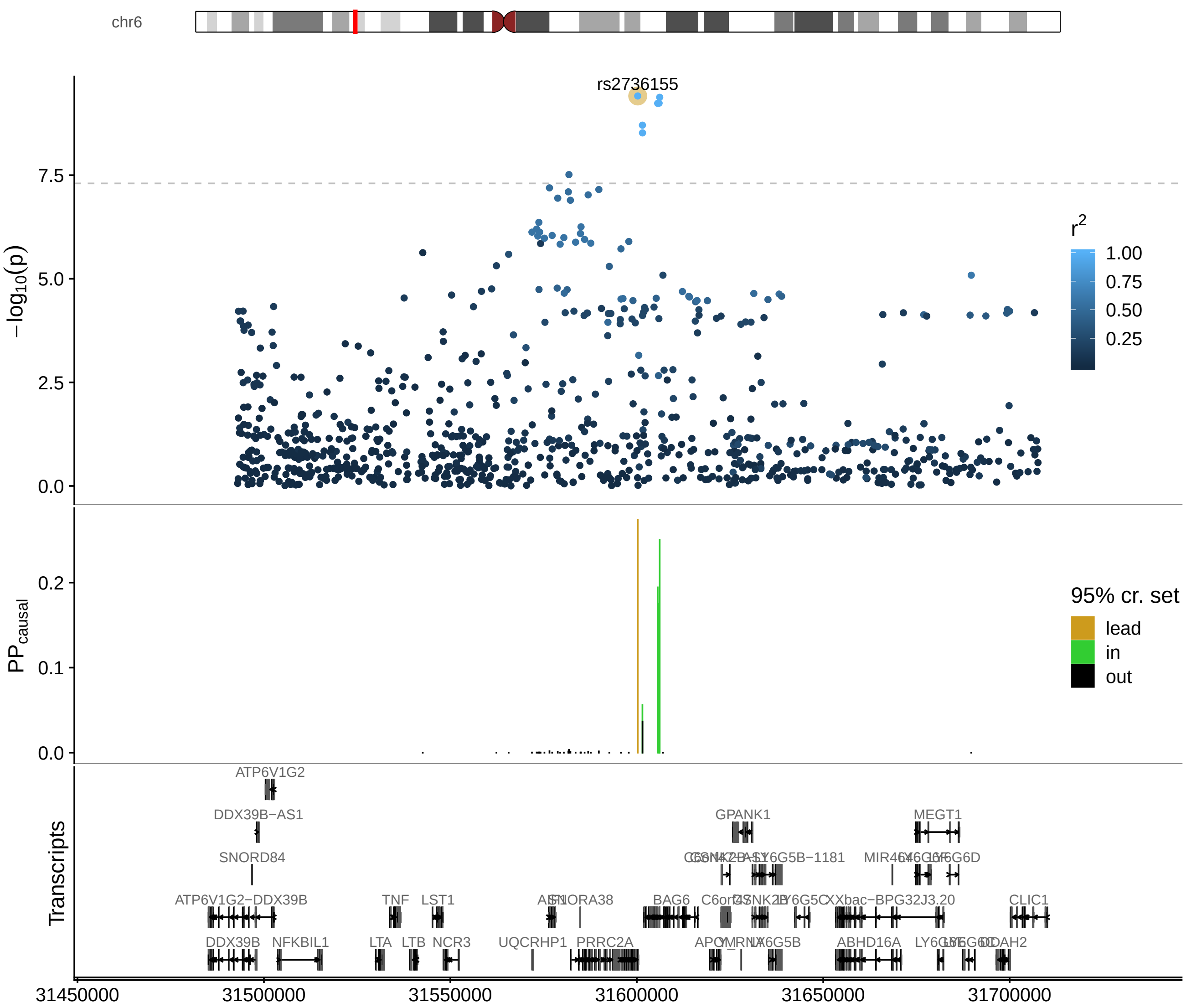
chr6
rs2736155
−log10(p)
r2
1.00
0.75
0.50
0.25
PPcausal
95% cr. set
lead
in
out
Transcripts
ATP6V1G2
DDX39B−AS1
SNORD84
ATP6V1G2−DDX39B
DDX39B
NFKBIL1
TNF
LST1
LTA
LTB
NCR3
UQCRHP1
PRRC2A
BAG6
GPANK1
ABHD16A
MEGT1
CLIC1
31450000
31500000
31550000
31600000
31650000
31700000

(c) rs2736155

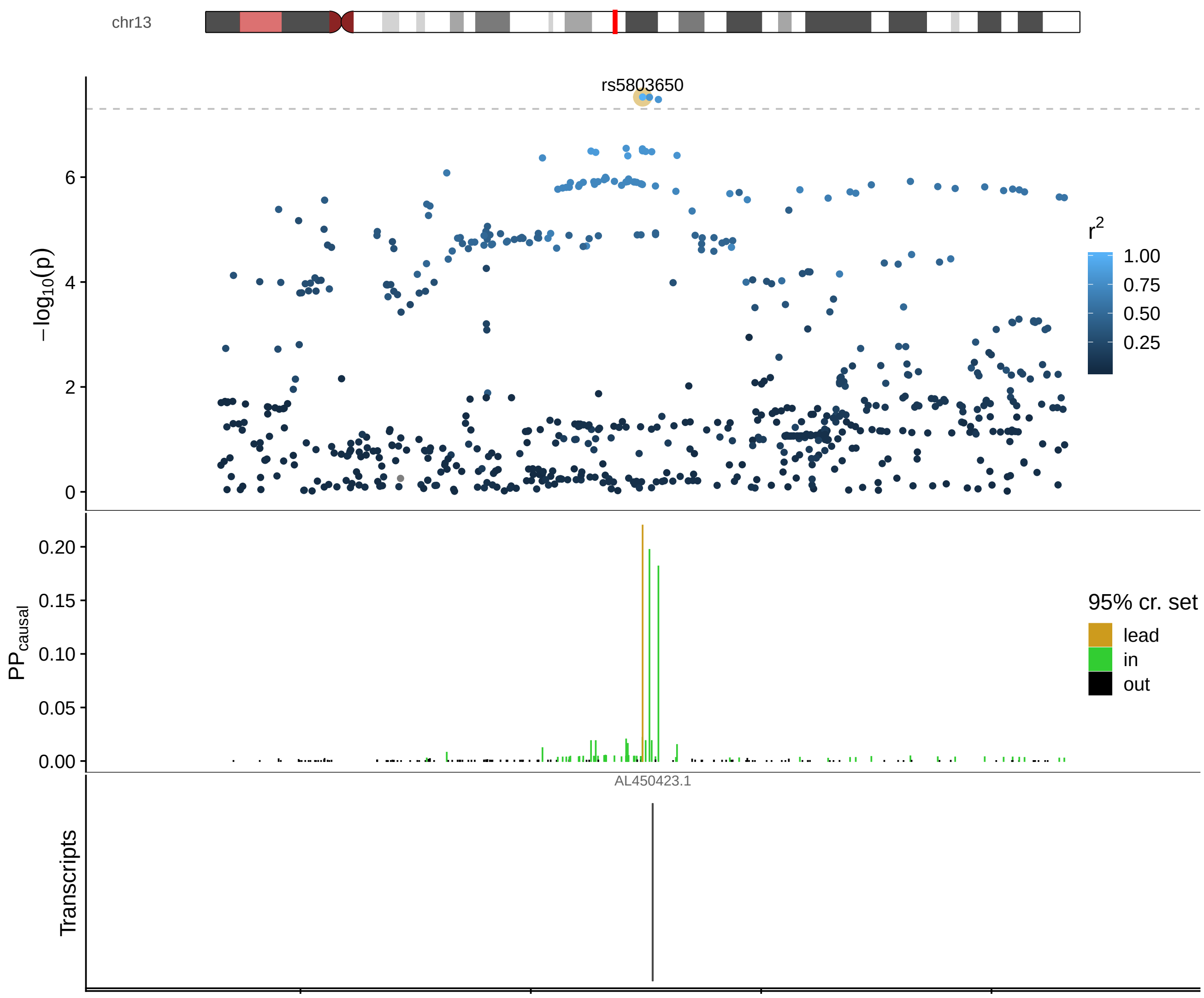
chr13
rs5803650
6
4
2
0
−log10(p)
r2
1.00
0.75
0.50
0.25
0.20
0.15
0.10
0.05
0.00
PPcausal
95% cr. set
lead
in
out
AL450423.1
Transcripts
53850000
53875000
53900000
53925000

(d) rs5803650

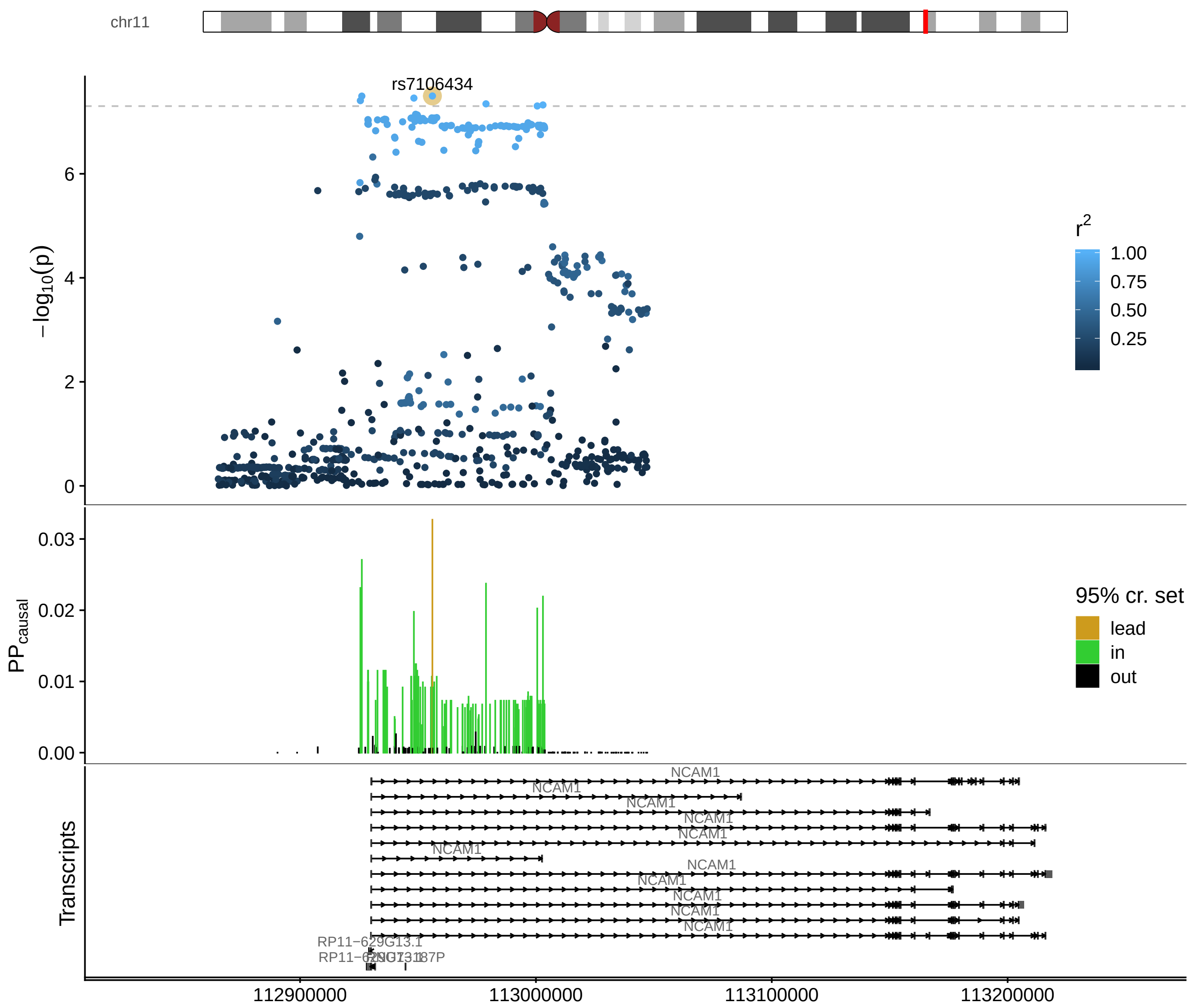

chr11
rs7106434
−log10(p)
r2
1.00
0.75
0.50
0.25
PPcausal
95% cr. set
lead
in
out
Transcripts
NCAM1
RP11−629G13.1
112900000
113000000
113100000
113200000

(e) rs7106434

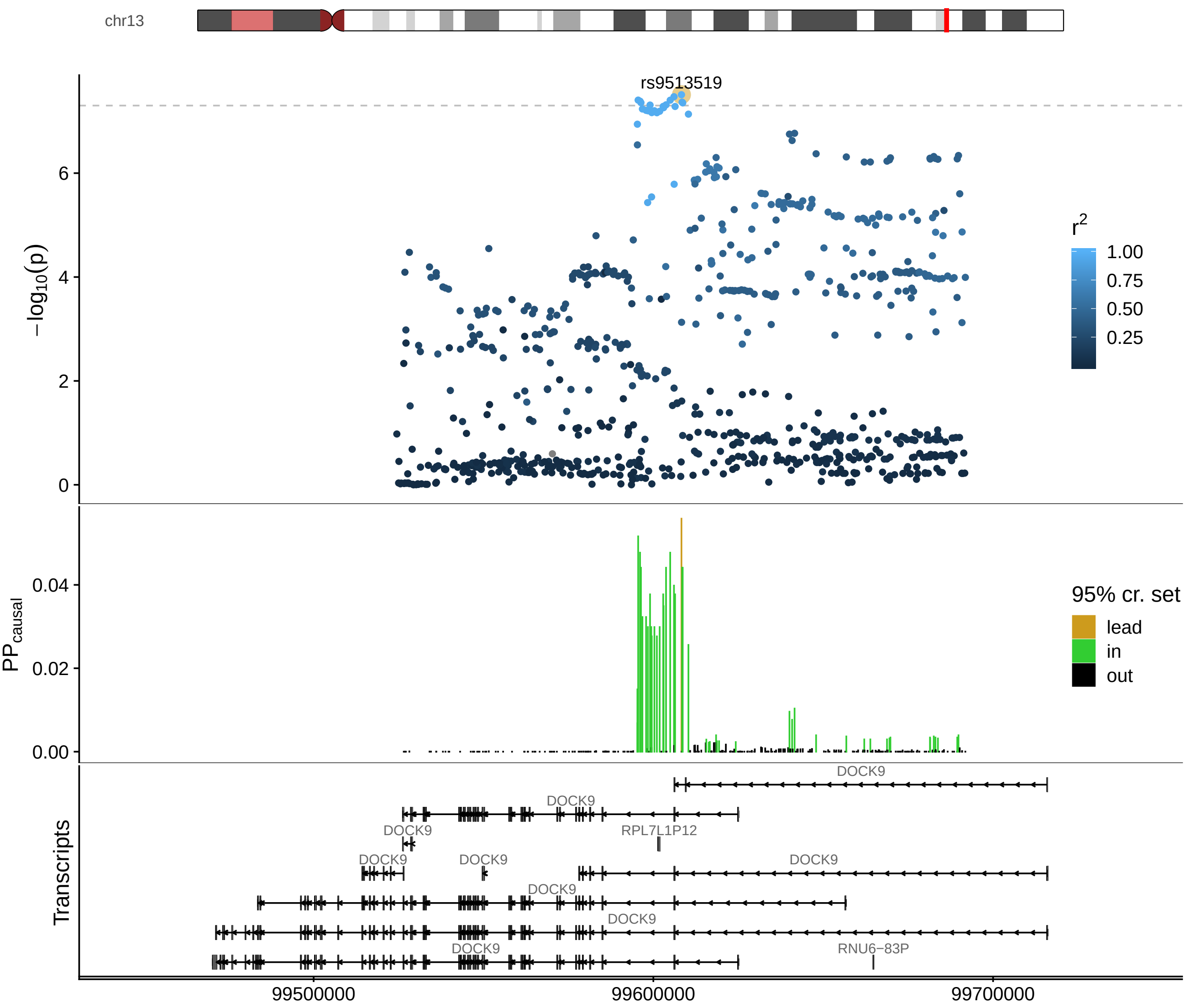

(f) rs9513519

Supplementary Figure 5: The position and local genetic environment of lead SNPs associated with IBS (highlighted in yellow) after clumping, and LD with neighboring SNPs (top pane). The dashed line indicates the genome-wide significance threshold. The middle pane shows the posterior probability of each variant in the window being causal (PP_{causal}), and whether or not it is contained in the 95% credible set (gold and green) of causal variants. Transcripts sourced from Ensembl are shown in the bottom pane. For rs2736155 on chromosome 6, only canonical transcripts are shown, considering the high gene density in the region.

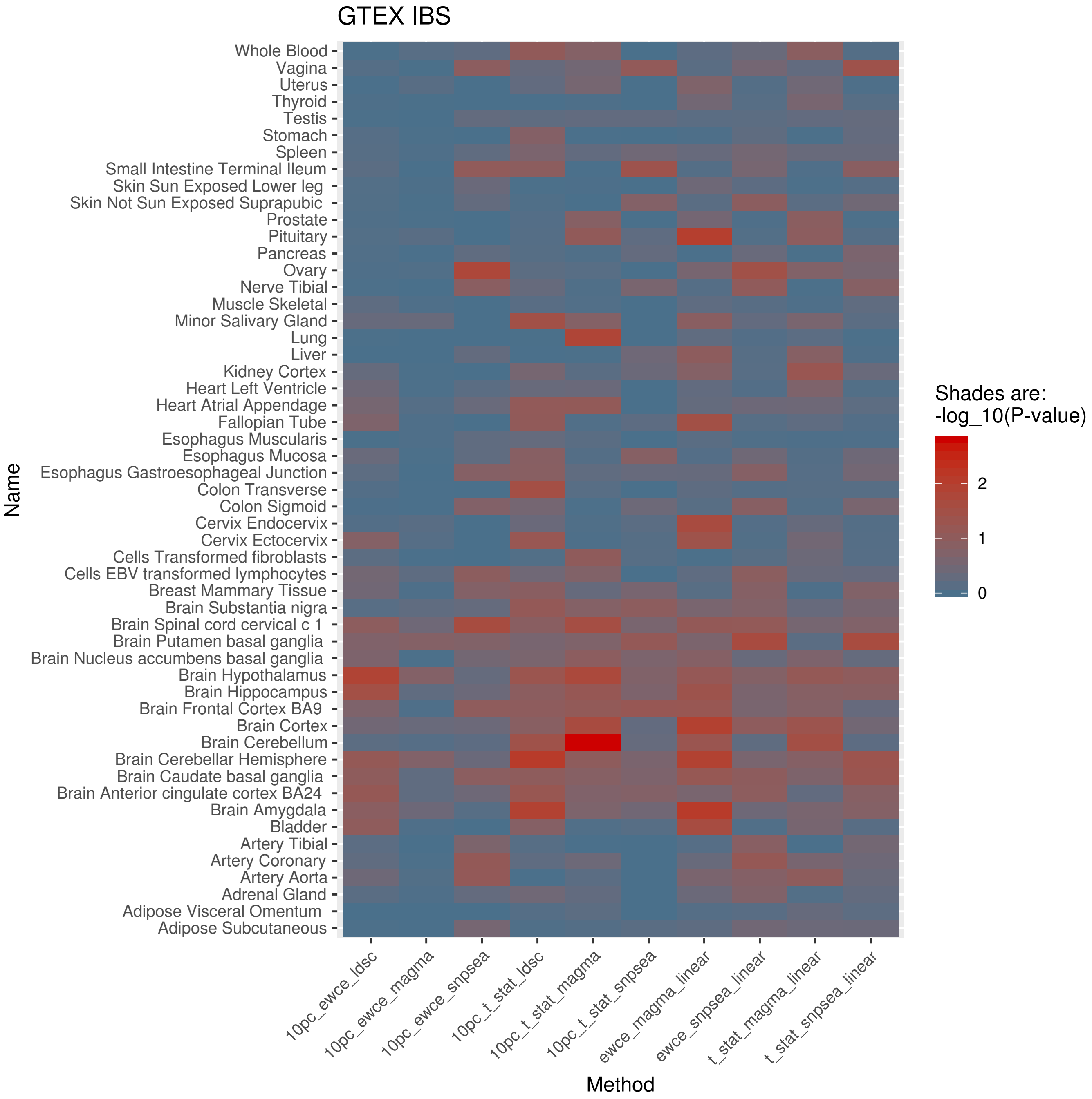

Supplementary Figure 6: Enrichment of IBS risk variants in genes specifically expressed in the brain and other tissues in GTEx. Cells shown in red are indicative of tissue-specific enrichment, while blue marks an absence of signal. These colours map to p-values from two-sided tests via various enrichment testing methods (LDSC-SEG, MAGMA and SNPsea). Genes specific to each GTEx tissue shown were determined based on either EWCE or t-statistic, in linear or top 10% (discrete gene-set) mode. No significant enrichment was observed after Benjamini-Hochberg multiple testing correction (neither per method nor across all tests), though brain tissues showed the strongest signal overall.

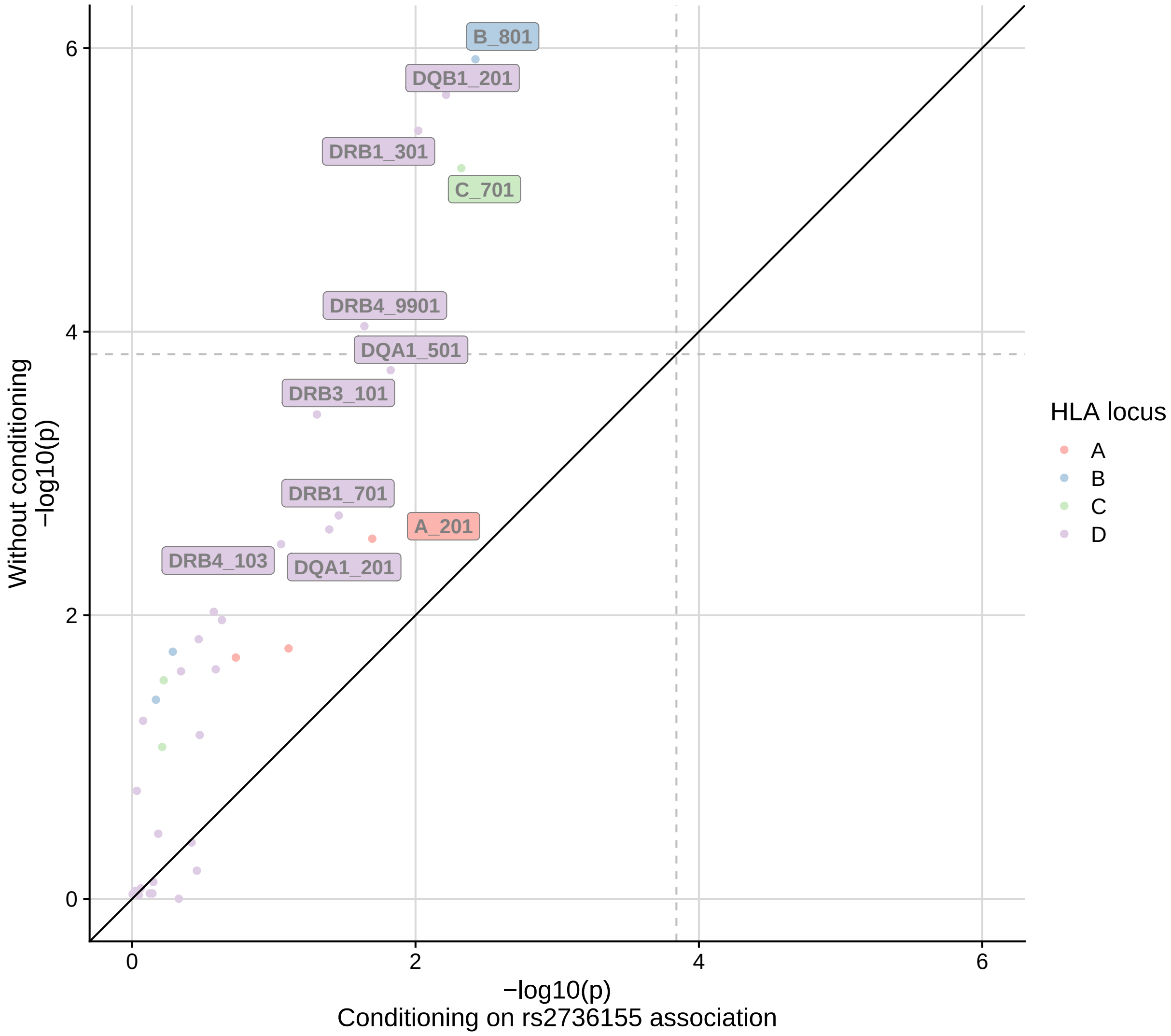

Supplementary Figure 7: HLA allele associations with IBS, with and without conditioning on the effect of rs2736155 (an IBS-associated SNP that is not in an HLA gene but is in the MHC region). UK Biobank codings for the alleles are shown for any association with $p<0.005$. Dashed line indicates the significance threshold after multiple testing correction (Bonferroni, $p=0.05/347$). Five HLA alleles were significant without conditioning on rs2736155, while none were significant with conditioning.

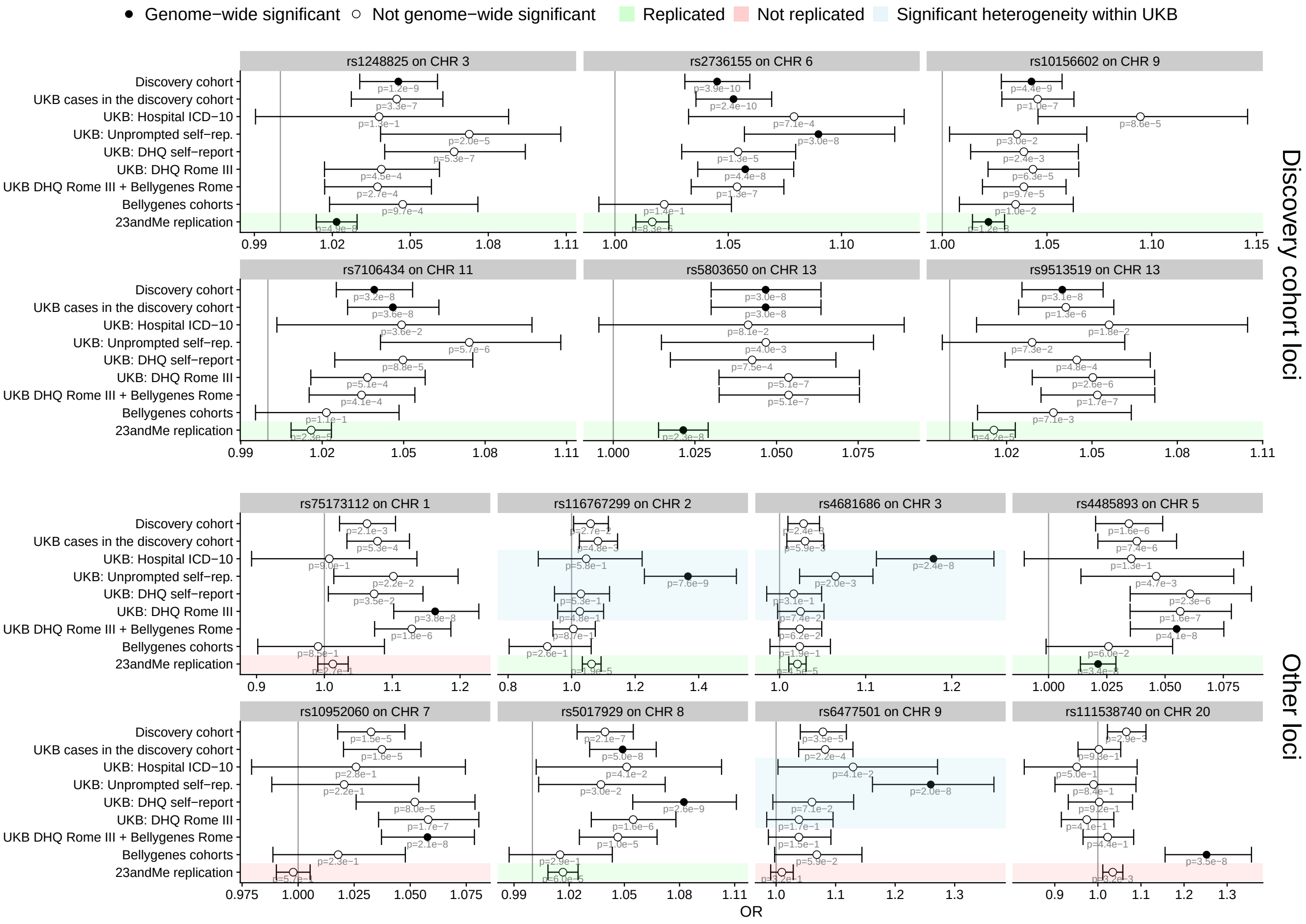

Supplementary Figure 8: Concordance between IBS definitions at variant level. Independent variants significantly associated with IBS in at least one of the IBS definitions along the y-axis, and their effect on IBS risk (OR and 95% CI) across these (p-values for two-sided tests using a linear mixed model for UK Biobank, an inverse-variance-weighted fixed-effect meta-analysis for the discovery cohort and BellyGenes, and a linear model for 23andMe). While the direction of effect was generally conserved between IBS definitions, some associations are only detected under one IBS definition (see e.g. rs116767299 on CHR 2, or r4681686 on CHR 3) and still replicated in an independent dataset (highlighted in green). Genetic effects at three loci varied significantly between the four UK Biobank (UKB) definitions of IBS (highlighted in blue, all with $p_{Q\neq 0}$ <0.05 for Cochran's Q as a measure of heterogeneity, Supplementary Table 14). Case and control samples sizes (in this order) for the definitions shown were as follows: Discovery cohort: 53400, 433201; UKB cases in the discovery cohort: 40548, 293220; UKB: Hospital ICD-10: 4237, 293220; UKB: Unprompted self-rep.: 9309, 293220; UKB: DHQ self-report: 16289, 293220; UKB: DHQ Rome III: 24845, 293220; UKB: DHQ Rome III + Bellygenes Rome: 28734, 317278; Bellygenes cohorts: 12852, 139981; 23andMe replication: 205252, 1384055.

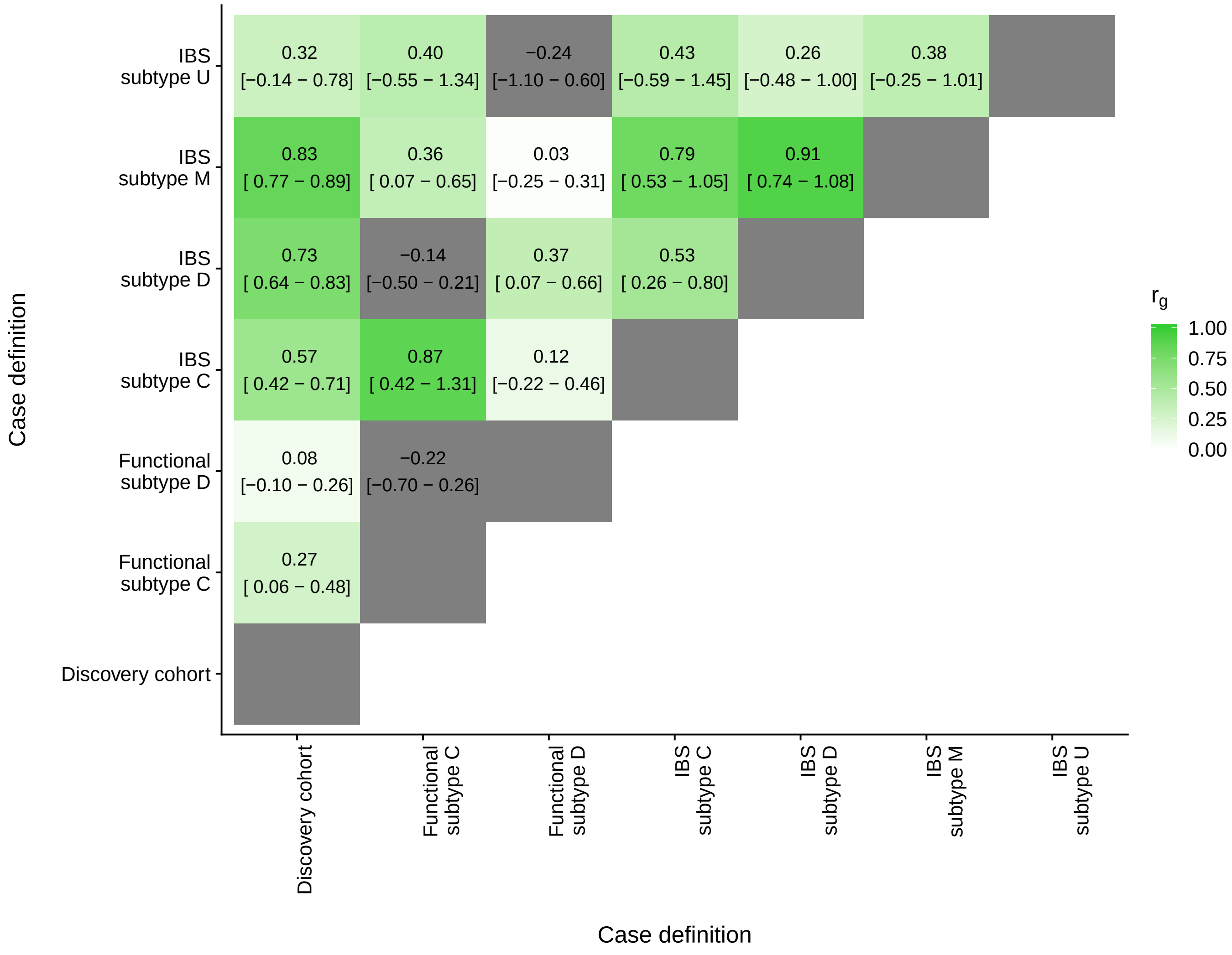

Supplementary Figure 9: Genetic correlations (r_g) between risk profiles for IBS subtypes, functional constipation (functional subtype C) and functional diarrhea (functional subtype D). Point estimates with 95% CI are shown. Negative values are shown in grey. The heritability of IBS subtype-U is not easily distinguishable from null (h2 Z-score < 4), so its values require careful interpretation.

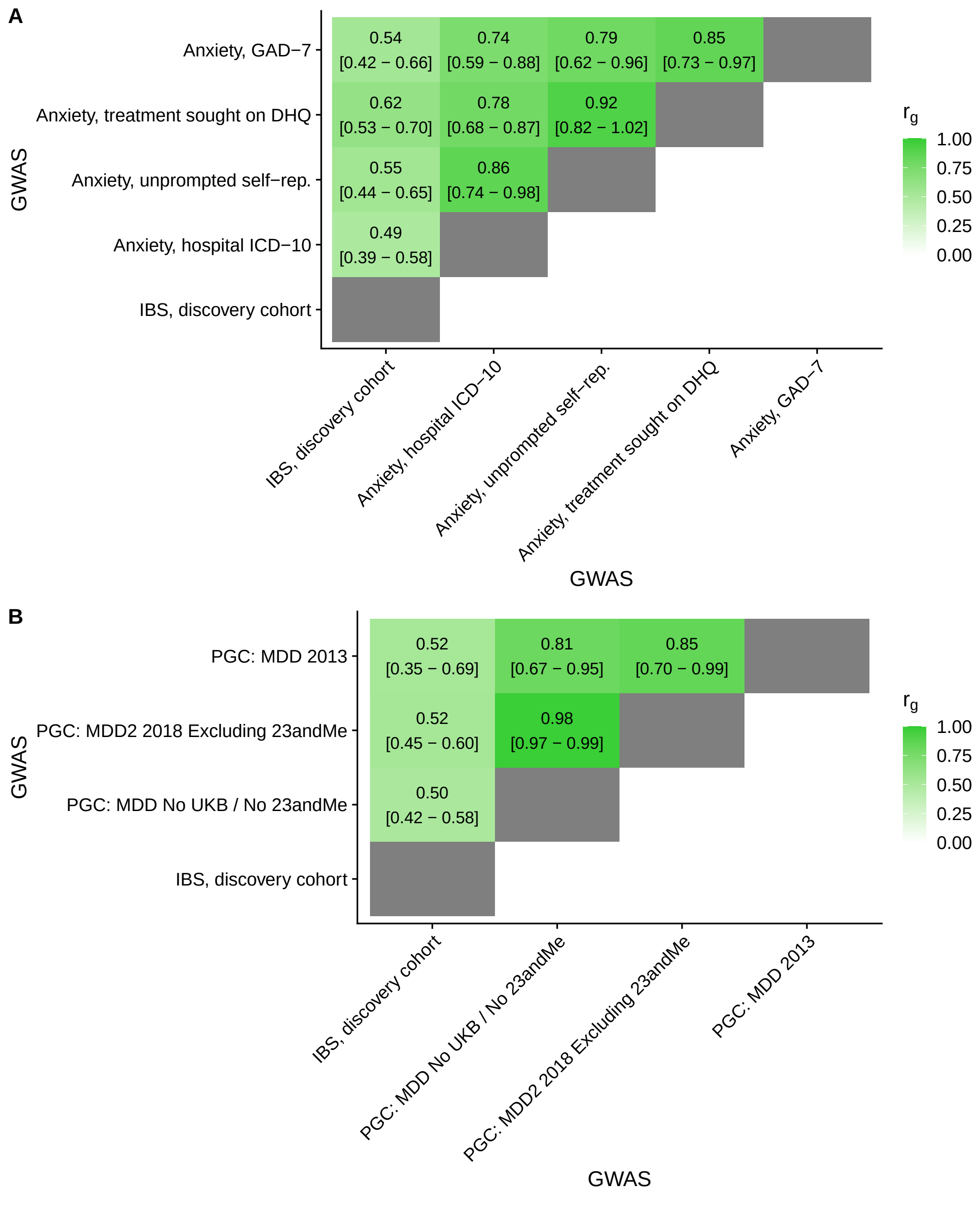

Supplementary Figure 10: Genetic correlations (r_g) between IBS and various definitions of anxiety or major depressive disorder (MDD) **A**. IBS was robustly correlated with anxiety in UK Biobank, independently of whether anxiety cases were defined by a GAD-7 score$\geq$10, by having sought treatment for anxiety, unprompted self-reporting of anxiety/panic attacks upon UK Biobank enrolment, or hospital records data in the form of ICD-10 codes. Controls were required not to have anxiety by any of these definitions. **B.** Genetic correlations between IBS and MDD were consistent across different definitions of MDD. Wray et al. (2018) cases "met standard criteria for MDD, were directly interviewed [...], or had medical record review by an expert diagnostician", and were supplemented by data employing "typical" case inclusion criteria from other consortia (see publication). We observed no significant difference depending on whether UK Biobank cases are or are not added to the above (PGC: MDD2 2018 Excluding 23andMe, PGC MDD No UKB / No 23andMe). In data from the Major Depressive Disorder Working Group of the Psychiatric GWAS consortium (2013, PGC: MDD 2013), cases were required to have a "diagnosis of DSM-IV lifetime MDD established using structured diagnostic instruments from direct interviews by trained interviewers, or clinician-administered DSM-IV checklists". All MDD data are available from the Psychiatric Genomics Consortium (PGC) website under the exact names provided here.

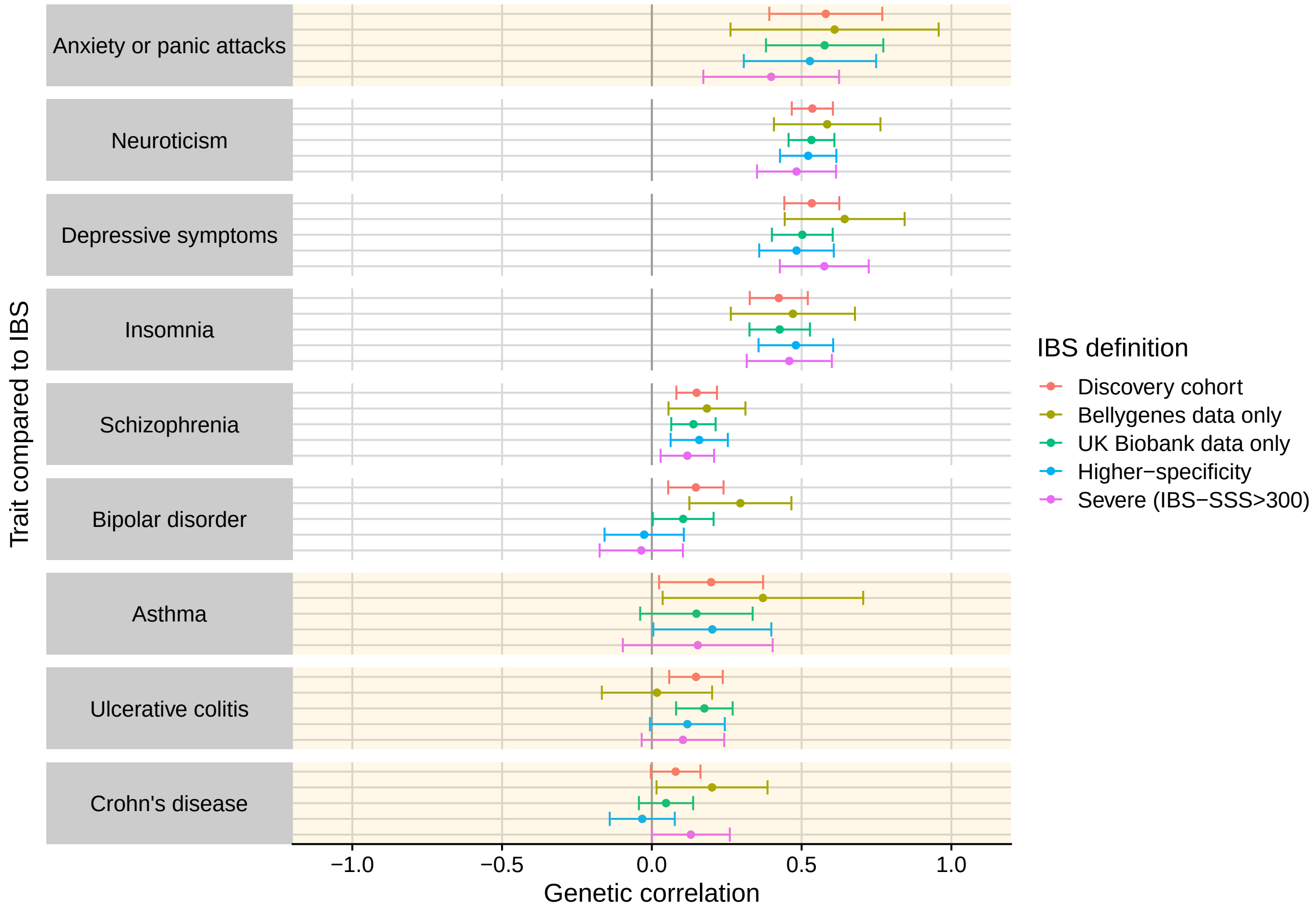

Supplementary Figure 11: Genetic correlations between various definitions of IBS and other traits. The selection of traits is identical to that in Figure 3, i.e. traits from peer-reviewed publications in LD Hub most genetically correlated with IBS, supplemented by traits selected for their clinical relevance (yellow). In the higher-specificity analysis, we have restricted cases to those meeting at least two of the four UK Biobank case definitions (Methods). The analysis of severe IBS was limited to DHQ respondents, with cases having an IBS-SSS>300. Across these definitions of IBS, the pattern of genetic correlation with mental health and personality traits remains consistent. Error bars represent a 95% CI. Case and control samples sizes (in this order) for the definitions shown were as follows: Discovery cohort: 53400, 433201; Bellygenes data only: 12852, 139981; UK Biobank data only: 40548, 293220; Higher-specificity: 11201, 293220; Severe (IBS-SSS>300): 4296, 72356.

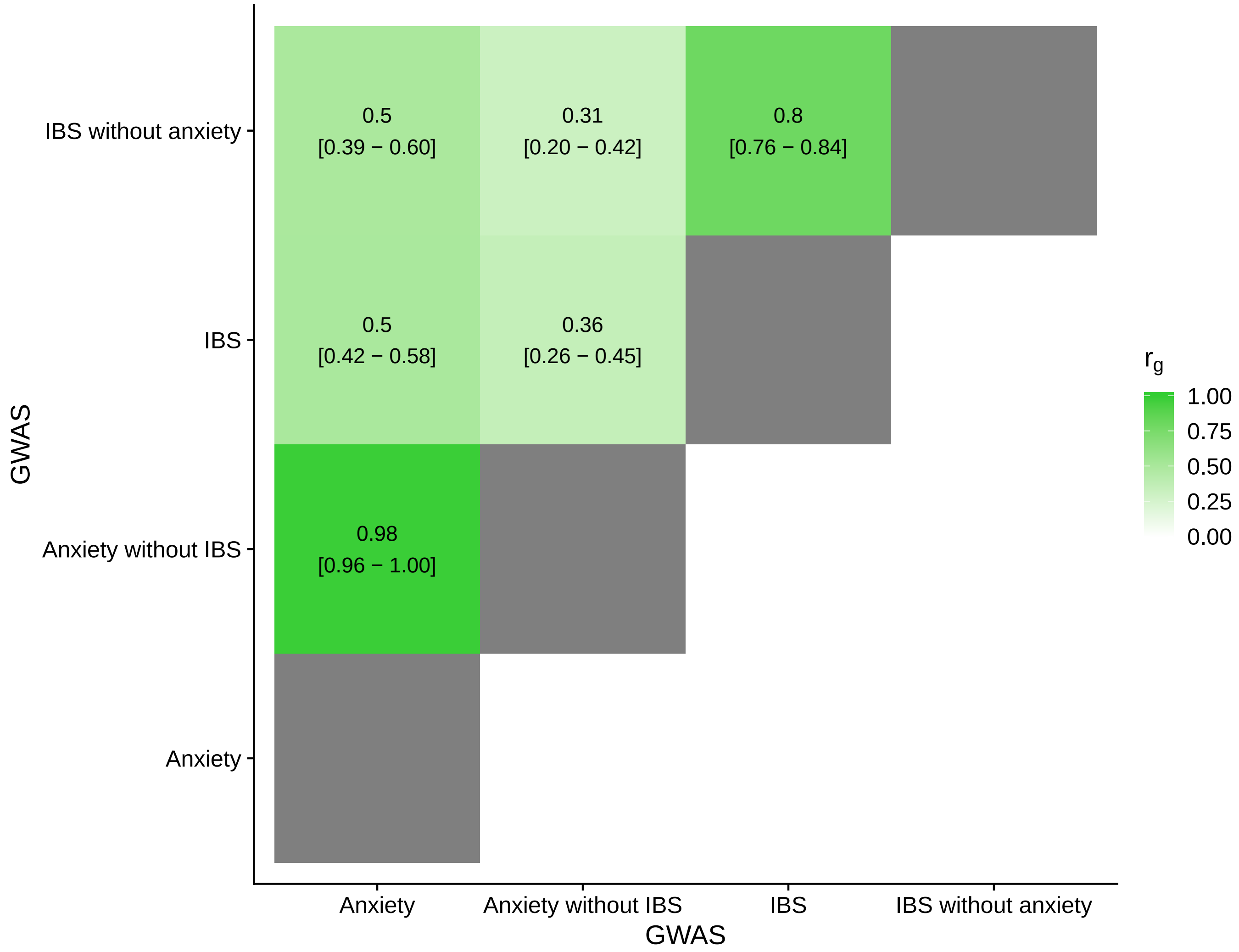

Supplementary Figure 12: Genetic correlations (r_g) between risk profiles for IBS with and without anxiety, and anxiety with and without IBS. Point estimates and 95% CIs are shown.

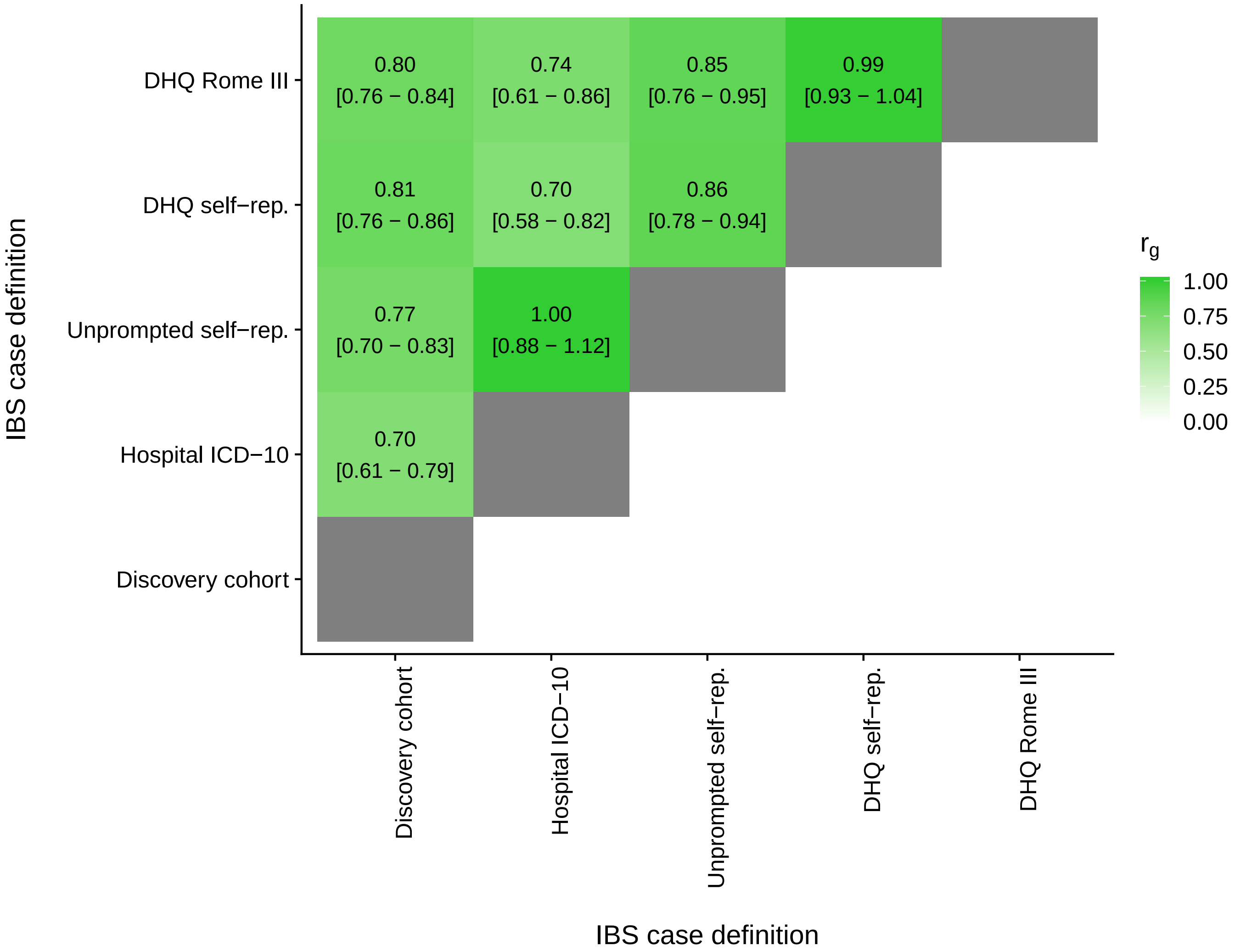

Supplementary Figure 13: High genetic correlations (r_g) between associations derived under different definitions of IBS, with a shared set of controls. Point estimates and 95% CIs are shown.

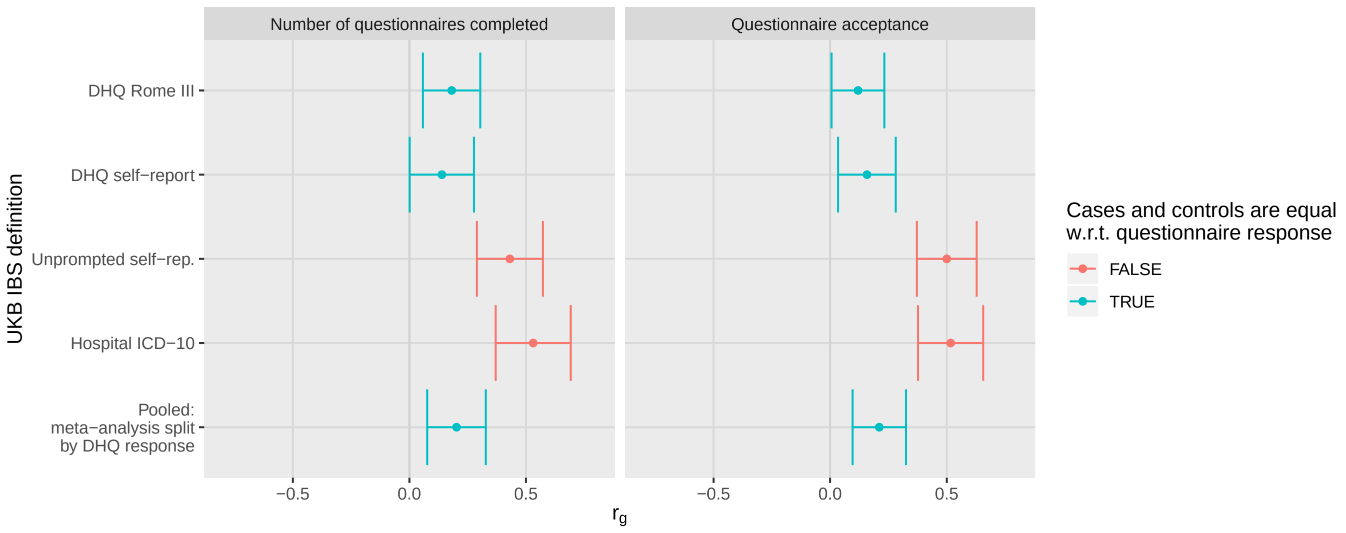

Supplementary Figure 14: The response to questionnaires correlated with genetic background, as exemplified by the response to a previous diet questionnaire run within UK Biobank (UKB, Rapid GWAS results from the Neale lab). Genetic correlations (shown with 95% CI) between IBS and questionnaire response vary based on how IBS cases were ascertained. Comparing controls who are DHQ respondents to cases that may not be (for Hospital ICD-10, Unprompted self-rep.) introduced a distinct shift in genetic correlation values. While the true genetic correlation between these traits (grey panels) and IBS may be non-zero, its value should be consistent if all diagnoses capture an identical IBS phenotype. Conducting the GWAS separately in DHQ respondents and non-respondents followed by meta-analysis (bottom label) should remove the impact of this potential source of bias. Case and control samples sizes (in this order) for the IBS definitions shown were as follows: DHQ Rome III: 24845, 293220; DHQ self-report: 16289, 293220; Unprompted self-rep.: 9309, 293220; Hospital ICD-10: 4237, 293220; Pooled: meta-analysis split by DHQ response: 40548, 293220.
