# Supplementary material for: Genome-wide analysis of 53,400 people with irritable bowel syndrome highlights shared genetic pathways with mood and anxiety disorders
Source: Nat Genet. 2021 Nov 5;53(11):1543–52. doi: 10.1038/s41588-021-00950-8 (PMC8571093; doi:10.1038/s41588-021-00950-8)
Supplement: Supplementary file 2 — Reporting Summary [file 41588_2021_950_MOESM2_ESM.pdf]

nature research

Corresponding author(s): Dr. Luke Jostins

Last updated by author(s): Jul 1, 2021

# Reporting Summary

Nature Research wishes to improve the reproducibility of the work that we publish. This form provides structure for consistency and transparency in reporting. For further information on Nature Research policies, see our Editorial Policies and the Editorial Policy Checklist.

## Statistics

For all statistical analyses, confirm that the following items are present in the figure legend, table legend, main text, or Methods section.

| n/a | Confirmed | |
|---|---|---|
| ☐ | ☒ | The exact sample size ($n$) for each experimental group/condition, given as a discrete number and unit of measurement |
| ☐ | ☒ | A statement on whether measurements were taken from distinct samples or whether the same sample was measured repeatedly |
| ☐ | ☒ | The statistical test(s) used AND whether they are one- or two-sided<br>*Only common tests should be described solely by name; describe more complex techniques in the Methods section.* |
| ☐ | ☒ | A description of all covariates tested |
| ☐ | ☒ | A description of any assumptions or corrections, such as tests of normality and adjustment for multiple comparisons |
| ☐ | ☒ | A full description of the statistical parameters including central tendency (e.g. means) or other basic estimates (e.g. regression coefficient) AND variation (e.g. standard deviation) or associated estimates of uncertainty (e.g. confidence intervals) |
| ☐ | ☒ | For null hypothesis testing, the test statistic (e.g. $F$, $t$, $r$) with confidence intervals, effect sizes, degrees of freedom and $P$ value noted<br>*Give P values as exact values whenever suitable.* |
| ☒ | ☐ | For Bayesian analysis, information on the choice of priors and Markov chain Monte Carlo settings |
| ☒ | ☐ | For hierarchical and complex designs, identification of the appropriate level for tests and full reporting of outcomes |
| ☐ | ☒ | Estimates of effect sizes (e.g. Cohen's $d$, Pearson's $r$), indicating how they were calculated |

*Our web collection on statistics for biologists contains articles on many of the points above.*

## Software and code

Policy information about availability of computer code

| | |
|---|---|
| Data collection | No relevant software was used to collect data used in the study. |
| Data analysis | BOLT-LMM was used to conduct the GWAS in the UK Biobank sample. Results were meta-analyzed with Bellygenes data using METAL (March 2011 version). We assigned loci to candidate genes using annotations from FUMA (v1.3.4), as well as from a colocalization analysis using Coloc (v3.2-1). To perform conditional analyses, we extracted SNPs associated with gastrointestinal disorders other than IBS from UK Biobank bgen data in expected dosage format using qctool 2.0.5 (https://www.well.ox.ac.uk/~gav/qctool_v2/). For clumping, we used PLINK version 2.00a2 and PLINK version 1.90b6.7. Use of qctool and PLINK is detailed in the Supplementary Note, as these tools were used for file format conversions rather than key analyses. We calculated SNP heritability and co-heritability (rg, genetic correlation) using univariate and bivariate LDSC (v1.0.0) against a range of traits via the LD Hub (v1.9.0) website. Further statistical analyses were carried out using R version 3.6.1. |

For manuscripts utilizing custom algorithms or software that are central to the research but not yet described in published literature, software must be made available to editors and reviewers. We strongly encourage code deposition in a community repository (e.g. GitHub). See the Nature Research guidelines for submitting code & software for further information.

## Data

Policy information about availability of data

All manuscripts must include a data availability statement. This statement should provide the following information, where applicable:

- Accession codes, unique identifiers, or web links for publicly available datasets
- A list of figures that have associated raw data
- A description of any restrictions on data availability

Genome-wide summary statistics have been deposited to the EBI GWAS catalogue (https://www.ebi.ac.uk/gwas/), under accession GCST90016564. Individual-level data on the DHQ responses, along with matching genotype, electronic health record and survey data, are available via an application to the UK Biobank Access

Management System (https://bbams.ndph.ox.ac.uk/ams/). Individual-level data for the23andMe were not shared as part of this project to protect the privacy of 23andMe participants. Data used for the analyses pertinent to the Bellygenes initiative include both individual-level and aggregate data. Individual-level data from the following sources can be obtained via applications to the respective biobanks and cohorts: TWINGENE (https://ki.se/en/research/swedish-twin-registry-for-researchers), HUNT (https://www.ntnu.edu/hunt/data), Michigan Genomics Initiative (https://precisionhealth.umich.edu/our-research/michigangenomics/), EGCUT (https://genomics.ut.ee/en/biobank.ee/data-access), LifeLines (https://www.lifelines.nl/researcher/how-to-apply), GEIRA (dbGaP Study Accession:phs000674.v2.p2). Data from IBS patients from tertiary centers can be requested from Mauro D'Amato at mdamato@cicbiogune.es, and may be made available depending on specific Material and Data Transfer Agreements with principal investigators at respective collaborating institutions.

# Field-specific reporting

Please select the one below that is the best fit for your research. If you are not sure, read the appropriate sections before making your selection.

☒ Life sciences ☐ Behavioural & social sciences ☐ Ecological, evolutionary & environmental sciences

For a reference copy of the document with all sections, see nature.com/documents/nr-reporting-summary-flat.pdf

# Life sciences study design

All studies must disclose on these points even when the disclosure is negative.

Sample size

This study did not recruit participants up until a target sample size was reached. Instead, this study uses the pre-defined UK Biobank sample, allowing participants to supply additional data on digestive health.

Data exclusions

UK Biobank participants with conditions such as coeliac disease, inflammatory bowel disease or previous intestinal resectional surgery which could result in IBS-like symptoms were excluded from both cases and digestively healthy controls to avoid signal contamination. These exclusion criteria were pre-established.

Replication

All genetic associations passing the genome-wide significance threshold in the discovery cohort were successfully replicated in an independent 23andMe dataset. The discovery analysis was performed once, and its results were replicated once.

Randomization

Participants were not randomly allocated into experiment groups. Instead, case and control groups for the genetic analyses were defined to have IBS or be digestively healthy. Differences between cases and controls were accounted for by including covariates such as age, sex, and transformations thereof in the genetic and phenotypic association analyses. Survey response bias was accounted for by analyzing respondents and non-respondents separately, then meta-analyzing the results.

Blinding

Data collectors were blinded to the genotype status of the participants, and participants were not made aware of their genotype status by UK Biobank. Unblinded genotype data was available to data analysts, but standard quality control and analysis software was used which makes blinding not relevant for this study.

# Behavioural & social sciences study design

All studies must disclose on these points even when the disclosure is negative.

Study description

*Briefly describe the study type including whether data are quantitative, qualitative, or mixed-methods (e.g. qualitative cross-sectional, quantitative experimental, mixed-methods case study).*

Research sample

*State the research sample (e.g. Harvard university undergraduates, villagers in rural India) and provide relevant demographic information (e.g. age, sex) and indicate whether the sample is representative. Provide a rationale for the study sample chosen. For studies involving existing datasets, please describe the dataset and source.*

Sampling strategy

*Describe the sampling procedure (e.g. random, snowball, stratified, convenience). Describe the statistical methods that were used to predetermine sample size OR if no sample-size calculation was performed, describe how sample sizes were chosen and provide a rationale for why these sample sizes are sufficient. For qualitative data, please indicate whether data saturation was considered, and what criteria were used to decide that no further sampling was needed.*

Data collection

*Provide details about the data collection procedure, including the instruments or devices used to record the data (e.g. pen and paper, computer, eye tracker, video or audio equipment) whether anyone was present besides the participant(s) and the researcher, and whether the researcher was blind to experimental condition and/or the study hypothesis during data collection.*

Timing

*Indicate the start and stop dates of data collection. If there is a gap between collection periods, state the dates for each sample cohort.*

Data exclusions

*If no data were excluded from the analyses, state so OR if data were excluded, provide the exact number of exclusions and the rationale behind them, indicating whether exclusion criteria were pre-established.*

Non-participation

*State how many participants dropped out/declined participation and the reason(s) given OR provide response rate OR state that no participants dropped out/declined participation.*

Randomization

*If participants were not allocated into experimental groups, state so OR describe how participants were allocated to groups, and if*

| | |
|---|---|
| Randomization | *allocation was not random, describe how covariates were controlled.* |

# Ecological, evolutionary & environmental sciences study design

All studies must disclose on these points even when the disclosure is negative.

| | |
|---|---|
| Study description | *Briefly describe the study. For quantitative data include treatment factors and interactions, design structure (e.g. factorial, nested, hierarchical), nature and number of experimental units and replicates.* |
| Research sample | *Describe the research sample (e.g. a group of tagged Passer domesticus, all Stenocereus thurberi within Organ Pipe Cactus National Monument), and provide a rationale for the sample choice. When relevant, describe the organism taxa, source, sex, age range and any manipulations. State what population the sample is meant to represent when applicable. For studies involving existing datasets, describe the data and its source.* |
| Sampling strategy | *Note the sampling procedure. Describe the statistical methods that were used to predetermine sample size OR if no sample-size calculation was performed, describe how sample sizes were chosen and provide a rationale for why these sample sizes are sufficient.* |
| Data collection | *Describe the data collection procedure, including who recorded the data and how.* |
| Timing and spatial scale | *Indicate the start and stop dates of data collection, noting the frequency and periodicity of sampling and providing a rationale for these choices. If there is a gap between collection periods, state the dates for each sample cohort. Specify the spatial scale from which the data are taken* |
| Data exclusions | *If no data were excluded from the analyses, state so OR if data were excluded, describe the exclusions and the rationale behind them, indicating whether exclusion criteria were pre-established.* |
| Reproducibility | *Describe the measures taken to verify the reproducibility of experimental findings. For each experiment, note whether any attempts to repeat the experiment failed OR state that all attempts to repeat the experiment were successful.* |
| Randomization | *Describe how samples/organisms/participants were allocated into groups. If allocation was not random, describe how covariates were controlled. If this is not relevant to your study, explain why.* |
| Blinding | *Describe the extent of blinding used during data acquisition and analysis. If blinding was not possible, describe why OR explain why blinding was not relevant to your study.* |

Did the study involve field work? ☐ Yes ☐ No

## Field work, collection and transport

| | |
|---|---|
| Field conditions | *Describe the study conditions for field work, providing relevant parameters (e.g. temperature, rainfall).* |
| Location | *State the location of the sampling or experiment, providing relevant parameters (e.g. latitude and longitude, elevation, water depth).* |
| Access & import/export | *Describe the efforts you have made to access habitats and to collect and import/export your samples in a responsible manner and in compliance with local, national and international laws, noting any permits that were obtained (give the name of the issuing authority, the date of issue, and any identifying information).* |
| Disturbance | *Describe any disturbance caused by the study and how it was minimized.* |

# Reporting for specific materials, systems and methods

We require information from authors about some types of materials, experimental systems and methods used in many studies. Here, indicate whether each material, system or method listed is relevant to your study. If you are not sure if a list item applies to your research, read the appropriate section before selecting a response.

## Materials & experimental systems

| n/a | Involved in the study | |
|---|---|---|
| ☒ | ☐ | Antibodies |
| ☒ | ☐ | Eukaryotic cell lines |
| ☒ | ☐ | Palaeontology and archaeology |
| ☒ | ☐ | Animals and other organisms |
| ☐ | ☒ | Human research participants |
| ☒ | ☐ | Clinical data |
| ☒ | ☐ | Dual use research of concern |

## Methods

| n/a | Involved in the study | |
|---|---|---|
| ☒ | ☐ | ChIP-seq |
| ☒ | ☐ | Flow cytometry |
| ☒ | ☐ | MRI-based neuroimaging |

## Antibodies

| | |
|---|---|
| Antibodies used | *Describe all antibodies used in the study; as applicable, provide supplier name, catalog number, clone name, and lot number.* |
| Validation | *Describe the validation of each primary antibody for the species and application, noting any validation statements on the manufacturer's website, relevant citations, antibody profiles in online databases, or data provided in the manuscript.* |

## Eukaryotic cell lines

Policy information about cell lines

| | |
|---|---|
| Cell line source(s) | *State the source of each cell line used.* |
| Authentication | *Describe the authentication procedures for each cell line used OR declare that none of the cell lines used were authenticated.* |
| Mycoplasma contamination | *Confirm that all cell lines tested negative for mycoplasma contamination OR describe the results of the testing for mycoplasma contamination OR declare that the cell lines were not tested for mycoplasma contamination.* |
| Commonly misidentified lines (See ICLAC register) | *Name any commonly misidentified cell lines used in the study and provide a rationale for their use.* |

## Palaeontology and Archaeology

| | |
|---|---|
| Specimen provenance | *Provide provenance information for specimens and describe permits that were obtained for the work (including the name of the issuing authority, the date of issue, and any identifying information).* |
| Specimen deposition | *Indicate where the specimens have been deposited to permit free access by other researchers.* |
| Dating methods | *If new dates are provided, describe how they were obtained (e.g. collection, storage, sample pretreatment and measurement), where they were obtained (i.e. lab name), the calibration program and the protocol for quality assurance OR state that no new dates are provided.* |

☐ Tick this box to confirm that the raw and calibrated dates are available in the paper or in Supplementary Information.

| | |
|---|---|
| Ethics oversight | *Identify the organization(s) that approved or provided guidance on the study protocol, OR state that no ethical approval or guidance was required and explain why not.* |

Note that full information on the approval of the study protocol must also be provided in the manuscript.

## Animals and other organisms

Policy information about studies involving animals; ARRIVE guidelines recommended for reporting animal research

| | |
|---|---|
| Laboratory animals | *For laboratory animals, report species, strain, sex and age OR state that the study did not involve laboratory animals.* |
| Wild animals | *Provide details on animals observed in or captured in the field; report species, sex and age where possible. Describe how animals were caught and transported and what happened to captive animals after the study (if killed, explain why and describe method; if released, say where and when) OR state that the study did not involve wild animals.* |
| Field-collected samples | *For laboratory work with field-collected samples, describe all relevant parameters such as housing, maintenance, temperature, photoperiod and end-of-experiment protocol OR state that the study did not involve samples collected from the field.* |
| Ethics oversight | *Identify the organization(s) that approved or provided guidance on the study protocol, OR state that no ethical approval or guidance was required and explain why not.* |

Note that full information on the approval of the study protocol must also be provided in the manuscript.

## Human research participants

Policy information about studies involving human research participants

| | |
|---|---|
| Population characteristics | UK Biobank participants were between ages 40-69 upon recruitment, and predominantly of European ancestry. A subset responded to the digestive health questionnaire (DHQ), one of the means used to identify IBS cases. Respondents have lower rates of IBS as measured via hospital ICD-10 codes (1.16% vs 1.40% among 171,061 respondents and 317,234 non-respondents, respectively), but not unprompted self-reporting (2.85% vs 2.30%). They also have lower rates of mental health disorders based on hospital ICD-10 codes (schizophrenia: 0.04% vs 0.21%, depression: 1.77% vs 3.40%) and unprompted self-reporting (schizophrenia: 0.05% vs 0.16%, depression: 5.83% vs 6.20%). Respondents also had a lower mean age than non-respondents (64.8 vs 65.8 years when the DHQ data were collected), and were more often female (56.7% vs 52.9%). Response rates also varied by ethnicity, e.g. 15.8% (1205 of 7645) among participants who report a Black or Black British background compared to 36.0% (165243 of 459256) among participants reporting a White ethnic background. Ultimately, the GWAS was conducted in a sample of European ancestry (this includes the Bellygenes population described in Table S9). |

| | |
|---|---|
| Recruitment | UK Biobank recruitment has been documented elsewhere, but in brief, it is known that its participants are disproportionately older, healthier and wealthier than the UK population as a whole. This, combined with the fact that the individuals analyzed were of European ancestry, means results may not generalize to the entire population, or populations from other ancestries.<br><br>This study expanded the UK Biobank dataset via a digestive health questionnaire (DHQ). The final digestive health questionnaire (available online as UK Biobank resource 595) was incorporated into the UK Biobank Questionnaire platform and advertised via email as an online questionnaire entitled 'digestive health'. Participants who had agreed to email contact were sent a hyperlink to their questionnaire, enabling linkage of results to other data in the UK Biobank dataset. The DHQ was also available on the participant area of the UK Biobank website and participants without email addresses received a flyer with their annual postal newsletter encouraging them to login to the participants' area of the UK Biobank website, and complete the questionnaire. Given that this resulted in a sample with a predominantly white British background (as is true for UK Biobank generally), genetic associations with IBS identified in this study may not generalize to non-European populations. Additionally, the fact that the sample consists of older individuals means that genetic association specific to IBS as it presents in younger individuals may be missed.<br><br>Recruitment of the Bellygenes cohorts is as described in the supplement. Briefly, the Bellygenes initiative is a large international collaboration that includes data from multiple biobanks, population-based cohorts, and patients from tertiary gastroenterology clinics worldwide. Three independent datasets were included in this study, based on different definitions of IBS: Rome Criteria for population-based cohorts HUNT, TWINGENE and LifeLines; ICD10 diagnoses from healthcare records for Mayo Genome Consortium, Estonia Genome Center at the University of Tartu, Michigan Genomics Initiative, and Genetic Epidemiology Research on Ageing from the Kasier Permanente biobank; gastroenterologist diagnosis for tertiary center patients. Except for patients from tertiary centers (who were recruited into IBS-specific studies at respective sites) the recruitment of participants into other cohorts was not focused on IBS or any other disease, as these are population-based cohorts (whose implementation has been described previously, see Supplementary Methods). As in the case of UK Biobank (whose "healthy volunteer" selection bias has been described) self-selection bias cannot be excluded for individuals from the Bellygenes initiative (comprising multiple population-based cohorts). Given the observed genetic link between mood/anxiety disorders and IBS, an overestimation of genetic risk effects may not be ruled out if increased propensity to volunteering data for research were assumed as common denominator for mood/anxiety disorders and IBS. |
| Ethics oversight | This study meta-analyzies UK Biobank and Bellygenes data. The UK Biobank digestive health questionnaire was approved as a substantial amendment to the UK Biobank protocol by the North West - Haydock REC, reference 11/NW/038e. The Bellygenes initiative study received ethical approval from Stockholm Ethics Examination Authority (EPN ID 2016/1620-31/2) and Monash University Human Research Ethics Committee (MUHREC ID 20326). Informed consent was obtained from all human participants. This study did not award compensation to any participants. |

Note that full information on the approval of the study protocol must also be provided in the manuscript.

## Clinical data

Policy information about clinical studies

All manuscripts should comply with the ICMJE guidelines for publication of clinical research and a completed CONSORT checklist must be included with all submissions.

| | |
|---|---|
| Clinical trial registration | *Provide the trial registration number from ClinicalTrials.gov or an equivalent agency.* |
| Study protocol | *Note where the full trial protocol can be accessed OR if not available, explain why.* |
| Data collection | *Describe the settings and locales of data collection, noting the time periods of recruitment and data collection.* |
| Outcomes | *Describe how you pre-defined primary and secondary outcome measures and how you assessed these measures.* |

## Dual use research of concern

Policy information about dual use research of concern

### Hazards

Could the accidental, deliberate or reckless misuse of agents or technologies generated in the work, or the application of information presented in the manuscript, pose a threat to:

| No | Yes | |
|---|---|---|
| ☐ | ☐ | Public health |
| ☐ | ☐ | National security |
| ☐ | ☐ | Crops and/or livestock |
| ☐ | ☐ | Ecosystems |
| ☐ | ☐ | Any other significant area |

## Experiments of concern

Does the work involve any of these experiments of concern:

| No | Yes | |
|---|---|---|
| ☐ | ☐ | Demonstrate how to render a vaccine ineffective |
| ☐ | ☐ | Confer resistance to therapeutically useful antibiotics or antiviral agents |
| ☐ | ☐ | Enhance the virulence of a pathogen or render a nonpathogen virulent |
| ☐ | ☐ | Increase transmissibility of a pathogen |
| ☐ | ☐ | Alter the host range of a pathogen |
| ☐ | ☐ | Enable evasion of diagnostic/detection modalities |
| ☐ | ☐ | Enable the weaponization of a biological agent or toxin |
| ☐ | ☐ | Any other potentially harmful combination of experiments and agents |

# ChIP-seq

## Data deposition

☐ Confirm that both raw and final processed data have been deposited in a public database such as GEO.

☐ Confirm that you have deposited or provided access to graph files (e.g. BED files) for the called peaks.

| | |
|---|---|
| Data access links<br>*May remain private before publication.* | *For "Initial submission" or "Revised version" documents, provide reviewer access links. For your "Final submission" document, provide a link to the deposited data.* |
| Files in database submission | *Provide a list of all files available in the database submission.* |
| Genome browser session<br>(e.g. UCSC) | *Provide a link to an anonymized genome browser session for "Initial submission" and "Revised version" documents only, to enable peer review. Write "no longer applicable" for "Final submission" documents.* |

## Methodology

| | |
|---|---|
| Replicates | *Describe the experimental replicates, specifying number, type and replicate agreement.* |
| Sequencing depth | *Describe the sequencing depth for each experiment, providing the total number of reads, uniquely mapped reads, length of reads and whether they were paired- or single-end.* |
| Antibodies | *Describe the antibodies used for the ChIP-seq experiments; as applicable, provide supplier name, catalog number, clone name, and lot number.* |
| Peak calling parameters | *Specify the command line program and parameters used for read mapping and peak calling, including the ChIP, control and index files used.* |
| Data quality | *Describe the methods used to ensure data quality in full detail, including how many peaks are at FDR 5% and above 5-fold enrichment.* |
| Software | *Describe the software used to collect and analyze the ChIP-seq data. For custom code that has been deposited into a community repository, provide accession details.* |

# Flow Cytometry

## Plots

Confirm that:

☐ The axis labels state the marker and fluorochrome used (e.g. CD4-FITC).

☐ The axis scales are clearly visible. Include numbers along axes only for bottom left plot of group (a 'group' is an analysis of identical markers).

☐ All plots are contour plots with outliers or pseudocolor plots.

☐ A numerical value for number of cells or percentage (with statistics) is provided.

## Methodology

| | |
|---|---|
| Sample preparation | *Describe the sample preparation, detailing the biological source of the cells and any tissue processing steps used.* |
| Instrument | *Identify the instrument used for data collection, specifying make and model number.* |

| | |
|---|---|
| Software | *Describe the software used to collect and analyze the flow cytometry data. For custom code that has been deposited into a community repository, provide accession details.* |
| Cell population abundance | *Describe the abundance of the relevant cell populations within post-sort fractions, providing details on the purity of the samples and how it was determined.* |
| Gating strategy | *Describe the gating strategy used for all relevant experiments, specifying the preliminary FSC/SSC gates of the starting cell population, indicating where boundaries between "positive" and "negative" staining cell populations are defined.* |

☐ Tick this box to confirm that a figure exemplifying the gating strategy is provided in the Supplementary Information.

# Magnetic resonance imaging

## Experimental design

| | |
|---|---|
| Design type | *Indicate task or resting state; event-related or block design.* |
| Design specifications | *Specify the number of blocks, trials or experimental units per session and/or subject, and specify the length of each trial or block (if trials are blocked) and interval between trials.* |
| Behavioral performance measures | *State number and/or type of variables recorded (e.g. correct button press, response time) and what statistics were used to establish that the subjects were performing the task as expected (e.g. mean, range, and/or standard deviation across subjects).* |

## Acquisition

| | |
|---|---|
| Imaging type(s) | *Specify: functional, structural, diffusion, perfusion.* |
| Field strength | *Specify in Tesla* |
| Sequence & imaging parameters | *Specify the pulse sequence type (gradient echo, spin echo, etc.), imaging type (EPI, spiral, etc.), field of view, matrix size, slice thickness, orientation and TE/TR/flip angle.* |
| Area of acquisition | *State whether a whole brain scan was used OR define the area of acquisition, describing how the region was determined.* |

Diffusion MRI ☐ Used ☐ Not used

## Preprocessing

| | |
|---|---|
| Preprocessing software | *Provide detail on software version and revision number and on specific parameters (model/functions, brain extraction, segmentation, smoothing kernel size, etc.).* |
| Normalization | *If data were normalized/standardized, describe the approach(es): specify linear or non-linear and define image types used for transformation OR indicate that data were not normalized and explain rationale for lack of normalization.* |
| Normalization template | *Describe the template used for normalization/transformation, specifying subject space or group standardized space (e.g. original Talairach, MNI305, ICBM152) OR indicate that the data were not normalized.* |
| Noise and artifact removal | *Describe your procedure(s) for artifact and structured noise removal, specifying motion parameters, tissue signals and physiological signals (heart rate, respiration).* |
| Volume censoring | *Define your software and/or method and criteria for volume censoring, and state the extent of such censoring.* |

## Statistical modeling & inference

| | |
|---|---|
| Model type and settings | *Specify type (mass univariate, multivariate, RSA, predictive, etc.) and describe essential details of the model at the first and second levels (e.g. fixed, random or mixed effects; drift or auto-correlation).* |
| Effect(s) tested | *Define precise effect in terms of the task or stimulus conditions instead of psychological concepts and indicate whether ANOVA or factorial designs were used.* |

Specify type of analysis: ☐ Whole brain ☐ ROI-based ☐ Both

| | |
|---|---|
| Statistic type for inference (See Eklund et al. 2016) | *Specify voxel-wise or cluster-wise and report all relevant parameters for cluster-wise methods.* |
| Correction | *Describe the type of correction and how it is obtained for multiple comparisons (e.g. FWE, FDR, permutation or Monte Carlo).* |

## Models & analysis

| n/a | Involved in the study |
| --- | --- |
| ☐ | ☐ Functional and/or effective connectivity |
| ☐ | ☐ Graph analysis |
| ☐ | ☐ Multivariate modeling or predictive analysis |

| | |
| --- | --- |
| Functional and/or effective connectivity | *Report the measures of dependence used and the model details (e.g. Pearson correlation, partial correlation, mutual information).* |
| Graph analysis | *Report the dependent variable and connectivity measure, specifying weighted graph or binarized graph, subject- or group-level, and the global and/or node summaries used (e.g. clustering coefficient, efficiency, etc.).* |
| Multivariate modeling and predictive analysis | *Specify independent variables, features extraction and dimension reduction, model, training and evaluation metrics.* |
