# Supplementary material for: Genome-wide analysis of 53,400 people with irritable bowel syndrome highlights shared genetic pathways with mood and anxiety disorders
Source: Nat Genet. 2021 Nov 5;53(11):1543–52. doi: 10.1038/s41588-021-00950-8 (PMC8571093; doi:10.1038/s41588-021-00950-8)
Supplement: Supplementary file 3 — Peer Review Information [file 41588_2021_950_MOESM3_ESM.pdf]

natureresearch

Peer Review Information

Journal: Nature Genetics
Manuscript Title: Genome-wide analysis of 53,400 people with irritable bowel syndrome highlights shared genetic pathways with mood and anxiety disorders
Corresponding author name(s): Dr Luke Jostins

Reviewer Comments & Decisions:

Decision Letter, initial version:

23rd February 2021

Dear Miles,

Your Article "Genome-wide analysis of 53,400 people with irritable bowel syndrome highlights shared pathways with neuroaffective traits" has been seen by two referees. You will see from their comments below that, while they find your work of interest, they have raised some relevant points. We are interested in the possibility of publishing your study in Nature Genetics, but we would like to consider your response to these points in the form of a revised manuscript before we make a final decision on publication.

To guide the scope of the revisions, the editors discuss the referee reports in detail within the team, including with the chief editor, with a view to identifying key priorities that should be addressed in revision and sometimes overruling referee requests that are deemed beyond the scope of the current study. In this case, we ask that you address all technical queries related to the association analyses and present further details on how the signals compare across different subgroups and cohorts with different diagnostic criteria. We also encourage you to examine sub-genome-wide significant variants from the discovery stage in the 23andMe cohort to potentially increase the yield of genome-wide significant loci in the combined analysis as suggested by Reviewer #1. We hope you will find this prioritized set of referee points to be useful when revising your study. Please do not hesitate to get in touch if you would like to discuss these issues further.

We therefore invite you to revise your manuscript taking into account all reviewer and editor comments. Please highlight all changes in the manuscript text file. At this stage we will need you to upload a copy of the manuscript in MS Word .docx or similar editable format.

We are committed to providing a fair and constructive peer-review process. Do not hesitate to contact

us if there are specific requests from the reviewers that you believe are technically impossible or unlikely to yield a meaningful outcome.

When revising your manuscript:

*1) Include a “Response to referees” document detailing, point-by-point, how you addressed each referee comment. If no action was taken to address a point, you must provide a compelling argument. This response will be sent back to the referees along with the revised manuscript.

*2) If you have not done so already please begin to revise your manuscript so that it conforms to our Article format instructions, available
here.
Refer also to any guidelines provided in this letter.

*3) Include a revised version of any required Reporting Summary:
https://www.nature.com/documents/nr-reporting-summary.pdf
It will be available to referees (and, potentially, statisticians) to aid in their evaluation if the manuscript goes back for peer review.
A revised checklist is essential for re-review of the paper.

Please be aware of our guidelines on digital image standards.

Please use the link below to submit your revised manuscript and related files:

[REDACTED]

Note: This URL links to your confidential home page and associated information about manuscripts you may have submitted, or that you are reviewing for us. If you wish to forward this email to co-authors, please delete the link to your homepage.

We hope to receive your revised manuscript within 6-8 weeks. If you cannot send it within this time, please let us know.

Please do not hesitate to contact me if you have any questions or would like to discuss these revisions further.

Nature Genetics is committed to improving transparency in authorship. As part of our efforts in this direction, we are now requesting that all authors identified as ‘corresponding author’ on published papers create and link their Open Researcher and Contributor Identifier (ORCID) with their account on the Manuscript Tracking System (MTS), prior to acceptance. ORCID helps the scientific community achieve unambiguous attribution of all scholarly contributions. You can create and link your ORCID from the home page of the MTS by clicking on ‘Modify my Springer Nature account’. For more information please visit please visit www.springernature.com/orcid.

We look forward to seeing the revised manuscript and thank you for the opportunity to review your

work.

Sincerely,
Kyle

Kyle Vogan, PhD
Senior Editor
Nature Genetics
https://orcid.org/0000-0001-9565-9665

Referee expertise:

Referee #1: Genetics, common diseases, gastrointestinal disorders

Referee #2: Genetics, common diseases, neuroaffective disorders

Reviewers' Comments:

Reviewer #1:
Remarks to the Author:

Previous GWAS of IBS have yielded little in terms of robust genetic associations. This is an important study that identified some of the first genome-wide significant loci associated with IBS risk. The authors combined IBS GWAS results from UK Biobank with the Bellygenes consortium to identify six genome-wide significant loci, all of which replicated in an independent 23andMe cohort. Additional analyses suggested there was little sharing of genetic risk with other gastrointestinal diseases, but significant sharing with neuro-related phenotypes such as anxiety and depression.

Major comments:

At ~200k cases, the 23andMe cohort is almost four times larger than the discovery cohorts. This seems like a missed opportunity to uncover a greater number loci and improve understanding of IBS biology. I can sympathize that there may be roadblocks in accessing full 23andMe summary statistics and performing meta-analysis, but was it not possible to look up sub genome-wide significant variants from the UKB+Bellygenes results in 23andMe, many of which would likely easily exceed p<5e-8 when combined?

Related to the point above, it is a little unclear how many variants were looked up in 23andMe and how these variants were selected. Page 19 of the supplemental text states that "all but two of the 109 variants submitted were matched in the 23andMe dataset." Later on page 26 in the supplement, it implies that 22 independent associations were looked up ("N where N=22 associations"). In the main text, it implies that 12 variants (along with their proxies) were looked up - six loci from the primary analysis, and six loci that reached genome-wide significance in one of the subgroups.

Given the different IBS definitions, I am interested in how the effect sizes at the genome-wide

significant loci differ between various subgroups. Some of this information is captured in Figure S8, though I wonder whether using ORs and 95%CIs can better represent these data than Z-scores. For instance, it is not obvious whether a Z-score in one group is lower than another because of a larger SE or because the OR is closer to 1. Knowing whether the CIs overlap between certain groups would also be informative. To that end, I wonder if a Forest plot-like representation of this would be more suitable?

Similarly, what was the degree of heterogeneity between the subgroups presented in Table S8 and the Bellygenes and 23andMe cohorts? The authors note that some associations detected in one subgroup do "Surprisingly... replicate in an independent dataset". What were the effect sizes at these variants in Bellygenes and 23andMe? Is it possible to include these in the Forest plot as suggested above?

I believe the lack of replication of the female-only association with unprompted self-report IBS from Bonfiglio et al. (2018) warrants further discussion. This seems more odd since the previous study used largely the same UKB dataset with a noisier definition of IBS. Did the authors also perform sex-stratified analysis at this variant using their more comprehensive case definition? Were the results at this variant even nominally significant? Was it significant in their subgroup analysis using only unprompted self reports?

Please clarify the number of controls in the discovery cohort. On line 173, it says there were 433,201 controls in the discovery cohort (also repeated on line 344). However, summing the number of controls in UKB (360,845) and Bellygenes (139,981) gives a total of 500,826 controls (lines 175-176).

Table S20 - please include an effect size column, or some other indication about direction of effect of the related phenotype relative to IBS (e.g. does the IBS risk-increasing allele of rs10156602 increase or decrease childhood BMI?).

Given the strong genetic correlations with a range of neuro-related phenotypes, why did the authors only perform MR analysis on anxiety? Were MR results similar for IBS and the other phenotypes in Figure 3 with significant rg?

Minor comments

In Table S13, some SE numbers are 0.00E+00

Line 270-271. I understand what the authors are trying to convey, but I disagree with the statement that low SNP-heritability is reflected in the low ORs from this study. It is entirely possible in theory to have low SNP-heritability and large ORs if variants are rare.

In the description of the GCTA COJO analysis in the supplemental text page 25, please provide the definition of "upstream"/"downstream", and specify what individual-level genotype data were used as the LD reference.

Reviewer #2:
Remarks to the Author:

This paper reports the results of a large GWAS study of Irritable Bowel Syndrome (IBS) in the UK Biobank. The authors added a questionnaire to the UK Biobank to collect diagnostic information on the symptoms, subtypes and severity of IBS. GWAS of a broad phenotypic definition of IBS in the UK Biobank combined with another large hospital registry consortium, Bellygenes yielded 6 significant associations with SNPS that explained a small proportion of the variance of IBS (i.e., 5.6%). These findings were replicated in the large 23 and me sample using self-reported diagnosis of IBS. The other major focus of the paper was investigation of genetic overlap between IBS and anxiety, neuroticism and depression, which in the aggregate were found to share some genetic risk with IBS.

This is an interesting paper that seeks to identify the genomics of gut diseases unexplained by well characterized genetic diseases such as Crohn's and Ulcerative Colitis. As such, it is an idiopathic and likely heterogeneous group of conditions that have shared environmental and genetic factors with a range of other conditions as demonstrated in this report. The finding and replication of 6 loci advances the field and is of heuristic value for future studies of etiologic mechanisms underlying subsets of IBS.

However, I found that the second component of the paper that linked IBS to "neuroaffective" conditions was less compelling. The phenotypes ranged from current symptom checklists for anxiety and depression that yield highly nonspecific rates of significant mood and anxiety disorders across varying time periods. Some of the questions are queried over the past 3 months, whereas others ask about the current two weeks. These scales can all be elevated due to multiple physical and psychological stressors and have very little relevance to true mental disorders including mood and anxiety disorders. To date, GWAS have not explained a substantial proportion of these conditions likely due to the heterogeneity of disorders that may lead to elevated symptoms of distress. Thus, taken together with the very low explanatory findings for IBS, the link with an even more heterogeneous group of conditions that may affect the brain does not really advance our understanding of etiologic factors underlying IBS.

There are several specific issues that require clarification or discussion.

1. It is unclear why the authors chose to combine across all of the subgroups of IBS for the GWAS analyses rather than examine the subtypes with greatest diagnostic certainty or severity. The exceedingly high prevalence rate of IBS of 14.5% was surprising and likely indicates a mixture of truly heritable cases and those that may be time limited symptom elevations.

2. It was difficult to understand the samples and phenotypes for the IBS as well as psychological symptoms in the 3 samples, and how subsets were combined in the analyses. The Methods could include a more clear description so that the reader does not have to search the supplementary materials for this broad overview.

3. The diagnoses of IBS were quite different in the three samples, ranging from medical records in the Bellygenes cohort to self-reported questionnaires with dome of the Rome criteria I the UK Biobank to a few questions in 23 and me. How confident are the authors that they are really tapping the same disorder using these diverse methods?

4. The epidemiologic associations with IBS in the UK Biobank were quite interesting and could provide further insight into potential sources of heterogeneity. Could the subtypes of IBS be further characterized by potential environmental correlates that were described in this report?

5. The authors should discuss how the 50% response rate to the IBS questionnaire in the UK Biobank may have led to bias in the findings.

Author Rebuttal to Initial comments

Dr Kyle Vogan
Senior Editor
Nature Genetics

April 17th, 2021

Re: *Genome-wide analysis of 53,400 people with irritable bowel syndrome highlights shared pathways with neuroaffective traits*

Dear Kyle,

As per your invitation, we have attached a revised version of our manuscript, with changes and additions highlighted in green. Below, we have included a point-by-point response to the reviewers' comments.

We have introduced new data and clarified details in response to reviewers' questions on the impact of diagnostic criteria on our results. We are particularly happy with the forest plot suggested by reviewer 1, which makes interpreting this data much easier. We have also included summary data across all case definitions for all independent loci as a new table. In response to reviewer 2, we have analysed new case definitions (a high-specificity analysis and a severe IBS analysis), which produce similar results to previous analyses. We have re-written the Methods section to make our analyses easier to follow. We have clarified other technical details requested by the reviewers. None of these alterations change the overall conclusions of our manuscript.

We were unfortunately unable to obtain additional replication data from 23andMe on sub-genome-wide significant associations. Both the academic authors and our coauthors at 23andMe made significant efforts to request this data in light of reviewer comments, via external and internal applications, but ultimately discussions within the company concluded that providing this data would not be possible. This is obviously disappointing, as we agree with the reviewer on the value this data would add. However, our analysis remains, by far, the largest IBS genetic analysis ever performed, both in terms of sample size and yield of loci.

We have introduced other new analyses in response to reviewer comments. These include a more substantial Mendelian Randomization analysis, demonstrating that the complex bidirectional causal effects seen between anxiety and IBS generalizes to depression and neuroticism (though not to bipolar disorder and schizophrenia). They also include a new analysis, in response to reviewer 2's comments, demonstrating that genetic correlations between IBS and anxiety and major depressive disorder are robust to case definition used, and are not likely to be driven by effects unrelated to true mental disorders.

We are grateful to both reviewers for their detailed and constructive reviews, and for Nature Genetics for inviting us to resubmit a revised manuscript. We feel that our paper has been substantially improved as a result of the revisions that we have made, and (in our humble opinion) that this revised version would be a good candidate for publication in your journal.

Yours sincerely,

Prof Miles Parkes
University of Cambridge, UK

Dr Luke Jostins
University of Oxford, UK

Prof Mauro D'Amato
Karolinska Institute, Sweden
and Monash University,
Australia

Response to referees

Genome-wide analysis of 53,400 people with irritable bowel syndrome highlights shared pathways with neuroaffective traits

Reviewer #1:

Remarks to the Author:

Previous GWAS of IBS have yielded little in terms of robust genetic associations. This is an important study that identified some of the first genome-wide significant loci associated with IBS risk. The authors combined IBS GWAS results from UK Biobank with the Bellygenes consortium to identify six genome-wide significant loci, all of which replicated in an independent 23andMe cohort. Additional analyses suggested there was little sharing of genetic risk with other gastrointestinal diseases, but significant sharing with neuro-related phenotypes such as anxiety and depression.

We thank the reviewer for their positive comments on our manuscript.

Major comments:

At ~200k cases, the 23andMe cohort is almost four times larger than the discovery cohorts. This seems like a missed opportunity to uncover a greater number loci and improve understanding of IBS biology. I can sympathize that there may be roadblocks in accessing full 23andMe summary statistics and performing meta-analysis, but was it not possible to look up sub genome-wide significant variants from the UKB+Bellygenes results in 23andMe, many of which would likely easily exceed p<5e-8 when combined?

We agree that study of sub-genome-wide significant variants from the discovery cohort in the 23andMe panel would uncover a greater number of bona fide IBS loci. However, 23andMe have indicated that they are unable to provide details on sub-genome-wide significant associations as part of this study due to their policy on replication and data sharing. We had previously requested this at the point we were establishing our collaboration with them three years ago. Following this specific reviewer comment our 23andMe collaborators made a further internal request for replication data on the 24 additional independent loci in our discovery cohort associated with IBS at p<1e-6. Unfortunately, after discussions within 23andMe, this request was again declined. 23andMe did, however, provide additional data regarding a new genome-wide significant locus identified in the course of our analysis by 'severe IBS' as recommended by Reviewer 2 (see below).

Related to the point above, it is a little unclear how many variants were looked up in 23andMe and how these variants were selected. Page 19 of the supplemental text states that "all but two of the 109 variants submitted were matched in the 23andMe dataset." Later on page 26 in the supplement, it implies that 22 independent associations were looked up ("N where N=22 associations"). In the main text, it implies that 12 variants (along with their proxies) were looked up - six loci from the primary analysis, and six loci that reached genome-wide significance in one of the subgroups.

We apologise for the confusion that these different numbers have caused, in retrospect this was not well described. Below we have given a rather lengthy response (apologies again) to explain where the numbers of 109 and 22 came from in the previous version of the manuscript and fully explain our approach to this in the revised manuscript.

To consolidate genome-wide significant associations across our original analyses, we carried out clumping these for each analysis individually. This yielded 36 sets of three variants (the lead SNPs and two proxies), or 108 variants total (109 when the IBS-C analysis was included). We then removed sets between which variants overlapped (retaining the set with the best lead p-value), so as not to request data in duplicate. This produced 23 sets of variants (69 variants including proxies) that we sent to 23andMe for replication, of which 67 variants were returned with data. These 67 SNPs contained or tagged all of the original 36 clumps with r^2>0.965. This is the data that is present in Table S13.

Two of these 23 sets of variants were in high LD (r^2>0.9), so we decided to call this "22 independent loci" for the purposes of correcting for multiple testing in the previous version of the manuscript. In practice, however, we do not know that these 22 sets of variants are independent, as three other sets were in moderate LD (r^2=0.2-0.9). On reflection, given that our conditional analysis did not provide strong evidence that these loci in weak LD represented multiple signals at the same locus, we have decided to merge these moderate LD associations together into a list of 19 independent loci across all analyses. After adding in the new locus from the severe IBS analysis suggested by Reviewer 2, this gives 20 independent loci.

As a result, the current version of our paper corrects for N=20 associations. All our successfully replicated loci would replicate after correcting for 20, 22 or indeed 36 independent associations, so the results of the analysis are not sensitive to this choice.

We have now enumerated these 20 independent loci in Table S13 to make this easier to follow. They include 14 from key analyses, which includes the discovery cohort (six, all replicating) and other IBS definitions (eight, of which five replicate), all illustrated in the forest plot requested by the reviewer (Figure S8, below). For clarity, we show the summary statistics of these loci across these analyses in a new table (Table S14).

The 6 remaining loci came from GWAS of methodological variations (UKB cases in the discovery cohort without correcting for DHQ response), IBS subtypes (severe IBS), or intermediate traits used in the meta-analysis (Bellygenes tertiary care cohorts, DHQ-respondents in the discovery cohort). Only this last definition yielded a replicating hit. These data remain available in Table S13.

We have amended the Replication section in the supplement to more clearly explain which variants were sent to 23andMe for replication:

"Lead SNPs and two proxies for each independent association from each analysis were sent to 23andMe for replication in a cohort with 205,252 self-reported IBS cases and 1,384,055 controls. Across analyses,we sent 20 independent associations for replication (min. 250kb apart, max. r^2 of 0.2), which included 6 loci from the discovery analysis, 8 loci from the additional key definitions shown in Fig. S8, 1 from the severe IBS analysis, and 5 from GWAS of intermediate traits used in the meta-analysis and methodological variations.

All but two of variants submitted for replication, marked with an asterisk in Table S13, were matched in the 23andMe dataset. The variants missing from 23andMe data (6:31610189_TAAAG_T, rs746685195) both had proxies with $r^2 \geq 0.965$ in our dataset which were matched.

Associations were considered replicated if SNPs had identical directions of effect in both datasets, were significant in the 23andMe data ($p_{23andMe}<0.05/N$ where N=20 associations), and remained genome-wide significant following meta-analysis ($p_{meta}<5e-8$)."

We have also updated the results section to provide clarity:

"Eight additional loci showed genome-wide significant association with various IBS definitions (see methods) but not the whole discovery cohort, of which five replicated in 23andMe data (Fig. S8, Table S13, Table S14)."

Given the different IBS definitions, I am interested in how the effect sizes at the genome-wide significant loci differ between various subgroups. Some of this information is captured in Figure S8, though I wonder whether using ORs and 95%CIs can better represent these data than Z-scores. For instance, it is not obvious whether a Z-score in one group is lower than another because of a larger SE or because the OR is closer to 1. Knowing whether the CIs overlap between certain groups would also be informative. To that end, I wonder if a Forest plot-like representation of this would be more suitable?

We thank the reviewer for this suggestion, and have produced a forest plot that allows readers to clearly see how odds ratios and their 95% confidence intervals vary across IBS definitions. Whether or not hits from individual definitions replicate is still shown. Where there is significant heterogeneity between the associations observed under different UK Biobank-based definitions of IBS, we highlight this. The updated figure, a preview of which is shown below, replaces the previous Figure S8.

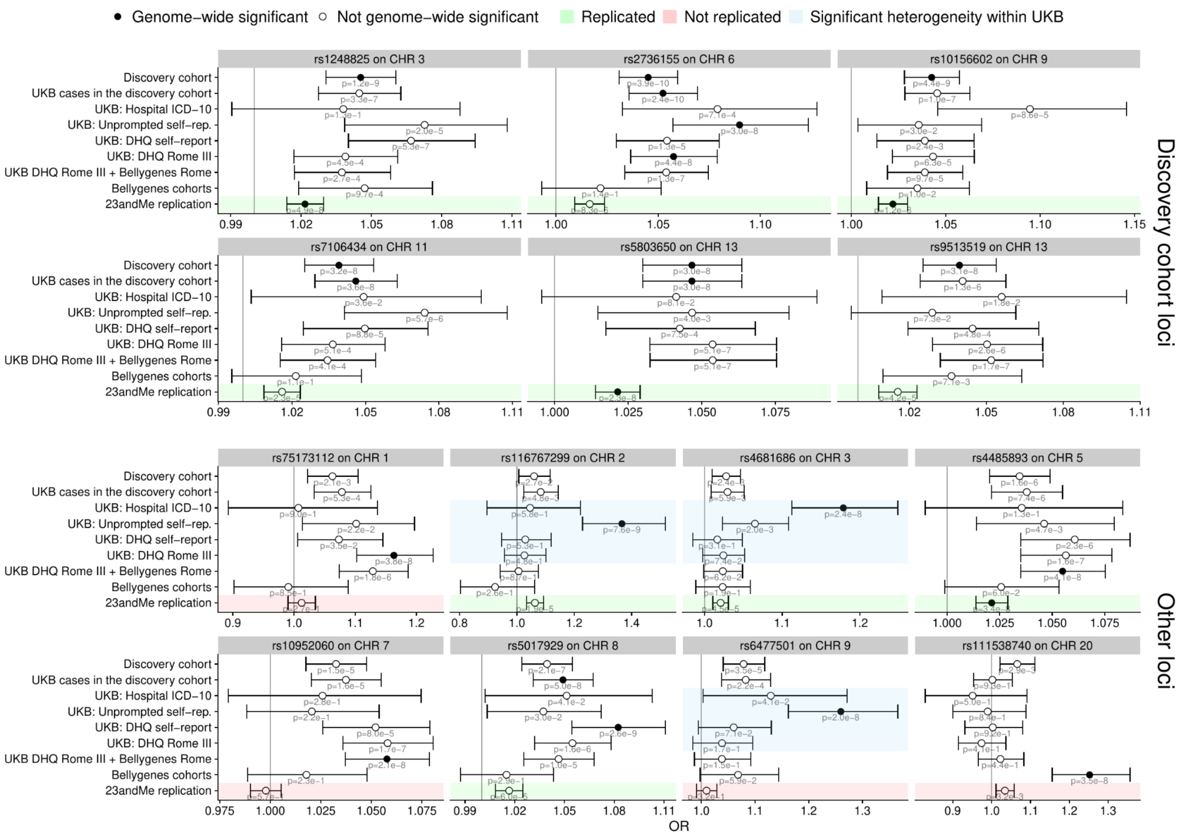

Figure S8: Concordance between IBS definitions at variant level. Independent variants significantly associated with IBS in at least one of the IBS definitions along the y-axis, and their effect on IBS risk (OR and 95% CI, p-value below) across these. While the direction of effect was generally conserved between IBS definitions, some associations were only detected under one IBS definition (see e.g. rs116767299 on CHR 2, or r4681686 on CHR 3) and still replicated in an independent dataset (highlighted in green). Genetic effects at three loci varied significantly between the four UK Biobank (UKB) definitions of IBS (highlighted in blue, all with $p_{Q\neq0}<0.05$ for Cochran's Q as a measure of heterogeneity, Table S14).

Similarly, what was the degree of heterogeneity between the subgroups presented in Table S8 and the Bellygenes and 23andMe cohorts? The authors note that some associations detected in one subgroup do "Surprisingly... replicate in an independent dataset". What were the effect sizes at these variants in Bellygenes and 23andMe? Is it possible to include these in the Forest plot as suggested above?

We have included the Bellygenes and 23andMe definitions of IBS in the same forest plot (Figure S8), as requested. Additionally, we now show data for the entire pool of UK Biobank IBS cases and the meta-analysis of Rome-based cases across UK Biobank and the Bellygenes cohort. Directions of effect for significant hits in any definition remain conserved in these datasets, as they were across individual definitions of IBS in UK Biobank.

We would like to thank the reviewer for their suggestions on this figure, as we feel that the new version is a substantial improvement and makes interpretation of the data across definitions substantially easier.

I believe the lack of replication of the female-only association with unprompted self-report IBS from Bonfiglio et al. (2018) warrants further discussion. This seems more odd since the previous study used largely the same UKB dataset with a noisier definition of IBS. Did the authors also perform sex-stratified analysis at this variant using their more comprehensive case definition? Were the results at this variant even nominally significant? Was it significant in their subgroup analysis using only unprompted self reports?

We agree this is an important point and have spent significant time trying to validate and understand the female-specific association for IBS reported in Bonfiglio et al. (2018). Specifically, we have run individual GWAS for each UK Biobank IBS case definition and subtype (hospital ICD-10, unprompted self-report, DHQ self-report, DHQ Rome III, DHQ Rome III type C, D and M) with cases and controls restricted to females only, as well as GWAS attempting to maximize and minimize overlap with the cases used in Bonfiglio et al.

The results of these analyses are now included in Table S15, and are referenced in the Result section of the main text:

"The female-specific signal previously identified(Bonfiglio et al. 2018) for unprompted self-reported IBS in UK Biobank was also observed in our female-specific analysis of unprompted self-reported data, but was not detected in female-specific analyses of any other case definitions from UK Biobank or the Bellygenes initiative, nor replicated in 23andMe unstratified analyses of both sexes (Table S15), possibly suggesting survey-specific factors playing a role."

While we were able to reproduce the previously reported association using the same case definition as in the original paper (unprompted self-report in females, p=1.00e-9 for N=6,918 cases, or p=4.29e-10 under 7,130 cases in Bonfiglio et al.), we did not observe the association in female-specific GWAS where that specific group of cases was excluded (p=0.63, in a larger group of N=22,309 cases).

Across other UK Biobank case definitions, partially overlapping the self-reported IBS cases, female-specific p-values ranged between 4.4e-3 and 0.89. Notably, although the SNP was associated with *unprompted* self-report of IBS diagnosis at UK Biobank enrollment as indicated above, it was not associated with *prompted* self-report of IBS diagnosis as ascertained in the digestive health questionnaire.

The unprompted self-report and DHQ self-report cases are sourced from responses to different questions: while the DHQ focuses on gastrointestinal disease and directly asks about a previous diagnosis of IBS, the IBS cases identified from the unprompted self-report definition had to have declared they "... had .. serious medical conditions or disabilities", which they then identified as IBS in a verbal interview with a qualified nurse. To ascertain

cases in this way requires the participant to perceive IBS as a 'serious medical condition' or to be more prone to reporting any medical condition as serious. In line with this hypothesis, the Bonfiglio et al. locus was also reported to associate with the "number of unprompted self-report illnesses" ($p=7.33\times10^{-3}$ in the Rapid GWAS from the Neale Lab, covering both sexes).

Please clarify the number of controls in the discovery cohort. On line 173, it says there were 433,201 controls in the discovery cohort (also repeated on line 344). However, summing the number of controls in UKB (360,845) and Bellygenes (139,981) gives a total of 500,826 controls (lines 175-176).

There were indeed 433,201 controls in the discovery cohort, and 139,981 controls across the Bellygenes data. The 360,845 figure is incorrect (that is, in fact, the sum of cohort controls and UK Biobank controls who did not respond to the DHQ). The correct number of UK Biobank controls, covering both respondents and non-respondent, is 293,220. We thank the reviewer for pointing this out, and have corrected it in the text:

> "We identified six independent IBS susceptibility loci at genome-wide significance ($p<5\times10^{-8}$) in a discovery cohort totaling 53,400 cases and 433,201 controls (Fig. 2 and Fig. S5). This results from pooling IBS cases across all case definitions to maximise power, in a meta-analysis of data from the UK Biobank (40,548 cases, 293,220 controls, Table S1, Table S2) and the international collaborative Bellygenes initiative (12,852 cases, 139,981 controls, see Methods and Table S9)."

Table S20 - please include an effect size column, or some other indication about direction of effect of the related phenotype relative to IBS (e.g. does the IBS risk-increasing allele of rs10156602 increase or decrease childhood BMI?).

We thank the reviewer for this suggestion, which is undoubtedly helpful for readers interested in these specific loci. We have added columns showing the effect and reference alleles for the association with IBS and the other trait in GWAS catalog (where reported), the effect allele frequency, and the reported effect (which, in GWAS catalog, varies between Z-scores and unit decrease/increase). We have also added a column that explicitly states whether directions of effects are opposite or identical between IBS and the GWAS catalog trait. This is now Table S22. We added a brief description to the Comparison to previous GWAS section of the Supplementary Methods:

> "Alleles were flipped in order to report GWAS catalogue effect sizes and directions relative to the IBS risk allele, with allele frequency used to check the consistency of the alleles (Table S22)."

Given the strong genetic correlations with a range of neuro-related phenotypes, why did the authors only perform MR analysis on anxiety? Were MR results similar for IBS and the other phenotypes in Figure 3 with significant rg?

We have now included Steiger MR analyses on depression, neuroticism, schizophrenia and bipolar disorder in Table S19 (well-powered non-UK Biobank derived publicly available summary statistics for insomnia are not available to our knowledge). The finding of bidirectional causality between IBS<->depression and IBS<->neuroticism are comparable to IBS<->anxiety (which we picked out as an exemplar), whereas schizophrenia and bipolar disorder do not show this pattern.

We have added a (very brief) mention of this to the main text:

> "Multiple models could explain our data (Table S19), but they were best explained by shared genetic risk pathways rather than causal effects between the two traits. Similar complex causal relationships are evident between IBS and other neuroaffective traits (Table S19)."

The methodology for these analyses is detailed in a new "Mendelian randomization analyses" section in the Supplementary Text.

> "We used unidirectional Mendelian randomization (inverse-variance weighted, IVW) and bidirectional Mendelian randomization (MR-Steiger),[27] implemented in the R package TwoSampleMR[28] (https://github.com/MRCIEU/TwoSampleMR) to test for evidence of causal effects of anxiety on IBS , using data from an orthogonal study of anxiety (measured via the GAD-2) in the Million Veterans Program.[29] We also carried out both MR analyses on all significant phenotypes from Figure 3 with non-UK Biobank summary statistics publicly available. The PubMed IDs of the publication that the non-IBS summary statistics were taken from are shown in Table S19 (only non-UK Biobank summary statistics were used to avoid sample overlap). When IBS was the exposure, the six discovery loci were used, and when IBS was the outcome, all independent genome-wide significant associations reported in the corresponding paper were used."

In practice, there is a significant amount of additional follow-up that could be carried out integrating IBS GWAS results with mental health, behavioural and other neuro-related phenotypes. Ultimately, while our results demonstrate clear shared genetic risk pathways between mental health and digestive health, they do not pinpoint the precise components of neuroaffective phenotypes that most strongly associate with IBS. Interpreting these second-order effects (comparing differences in the genetic relationship between different traits) would require considerable work on a trait-by-trait basis. We believe that this larger project is beyond the scope of this paper.

Minor comments

In Table S13, some SE numbers are 0.00E+00

We thank the reviewer for pointing out this formatting error and have corrected this in a new version of Table S13.

Line 270-271. I understand what the authors are trying to convey, but I disagree with the statement that low SNP-heritability is reflected in the low ORs from this study. It is entirely possible in theory to have low SNP-heritability and large ORs if variants are rare.

We agree that there is not necessarily a 1:1 mapping between effect sizes at common variants and SNP heritability. The use of the term "reflected" in the text was intended to indicate that these two findings were consistent with each other, rather than that one caused the other. To add clarity we have now changed the sentence to state these two findings separately:

> "IBS genome-wide SNP heritability was just 5.8% (SE<0.01) in the European ancestry population studied here, and the effect sizes of our susceptibility loci were modest (OR's <1.05)."

In the description of the GCTA COJO analysis in the supplemental text page 25, please provide the definition of "upstream"/"downstream", and specify what individual-level genotype data were used as the LD reference.

The upstream and downstream boundaries were defined by the clumping, i.e. the leftmost and rightmost SNPs that had $p < 0.05$ and at least $r^2>0.05$ with a lead SNP. The reference LD panel included 10,000 unrelated individuals passing genetic QC. We have updated the supplementary text to clarify and reflect this:

> "We extracted sets of all SNPs between the variants marking the boundaries of each clump, and used gcta-select in GCTA[16] 1.92.0 beta 1 to select independently associated SNPs, and to uncover potential signals attenuated via high-LD SNPs with opposite effect sizes. We used 10,000 unrelated individuals passing genetic QC in UK Biobank as a reference LD panel."

Reviewer #2:

Remarks to the Author:

This paper reports the results of a large GWAS study of Irritable Bowel Syndrome (IBS) in the UK Biobank. The authors added a questionnaire to the UK Biobank to collect diagnostic information on the symptoms, subtypes and severity of IBS. GWAS of a broad phenotypic definition of IBS in the UK Biobank combined with another large hospital registry consortium, Bellygenes yielded 6 significant associations with SNPS that explained a small proportion of the variance of IBS (i.e., 5.6%). These findings were replicated in the large 23 and me sample using self-reported diagnosis of IBS. The other major focus of the paper was investigation of genetic overlap between IBS and anxiety, neuroticism and depression, which in the aggregate were found to share some genetic risk with IBS.

This is an interesting paper that seeks to identify the genomics of gut diseases unexplained by well characterized genetic diseases such as Crohn's and Ulcerative Colitis. As such, it is an idiopathic and likely heterogeneous group of conditions that have shared environmental and genetic factors with a range of other conditions as demonstrated in this report. The finding and replication of 6 loci advances the field and is of heuristic value for future studies of etiologic mechanisms underlying subsets of IBS.

However, I found that the second component of the paper that linked IBS to "neuroaffective" conditions was less compelling. The phenotypes ranged from current symptom checklists for anxiety and depression that yield highly nonspecific rates of significant mood and anxiety disorders across varying time periods. Some of the questions are queried over the past 3 months, whereas others ask about the current two weeks. These scales can all be elevated due to multiple physical and psychological stressors and have very little relevance to true mental disorders including mood and anxiety disorders. To date, GWAS have not explained a substantial proportion of these conditions likely due to the heterogeneity of disorders that may lead to elevated symptoms of distress. Thus, taken together with the very low explanatory findings for IBS, the link with an even more heterogeneous group of conditions that may affect the brain does not really advance our understanding of etiologic factors underlying IBS.

> We thank the reviewer for their comments and observations. We agree with the reviewer that mental health (and the reasons why people fill out a GAD-7 form in a particular way on a particular day) is complex and multifactorial, and interpreting phenotypic or genetic correlations with specific measures of mental health is challenging. We do believe that for researchers studying IBS, the finding of a strong genetic link between IBS and anxiety, and the demonstration that this is driven, at least in part, by shared genetic pathways rather than mere co-occurrence, represents a step forward in our understanding of the etiologic factors of IBS.
>
> In response to this comment we have carried out further analyses to confirm that the genetic correlation with anxiety and depression that we observe is robust to the way these

conditions are defined, and does not merely reflect transient increases in scores from symptom checklists.

First, we have run GWASes of anxiety cases ascertained by four different means:

1. individuals who self-report being diagnosed with anxiety or panic attacks by a doctor,
2. individuals who self-report ever having sought treatment for anxiety,
3. individuals who have a diagnosis of an anxiety disorder (either phobic anxiety disorder or generalized anxiety disorder) coded in an electronic hospital record since 1997 and
4. individuals who on a GAD-7 symptomatic screening test for generalized anxiety scored highly (≥10) .

We find nearly identical coheritability estimates (r_g point estimates 0.49-0.62) between IBS and all of the above case definitions.

Second, we have looked at the genetic correlation between IBS and various additional definitions of major depressive disorder used by the Psychiatric Genomics Consortium (PGC). This includes definitions from the psychiatric GWAS consortium working group (2013), with cases required to have "diagnoses of DSM-IV lifetime MDD established using structured diagnostic instruments from direct interviews by trained interviewers, or clinician-administered DSM-IV checklists", and more recent depression GWAS data from Wray et al. (2018) in which cases ascertained using UK Biobank's questionnaires (or any other UK Biobank data) had been excluded. Also from these data, we find high and consistent genetic correlation between IBS and major depressive disorder (r_g point estimates 0.50-0.52).

The above analyses demonstrate that the finding of a genetic correlation between IBS and anxiety or depression is not related to a particular way of ascertaining the presence of these conditions. We have added a statement on the robustness of the genetic correlations to the main text and show the corresponding analyses in a new figure:

> "Across the genome, the same alleles that predispose to IBS also predispose to neuroaffective traits. The correlations were consistent regardless of the mode of diagnosis of anxiety or depression (Fig. S10).[34,35]"

A preview of Figure S10 is shown below:

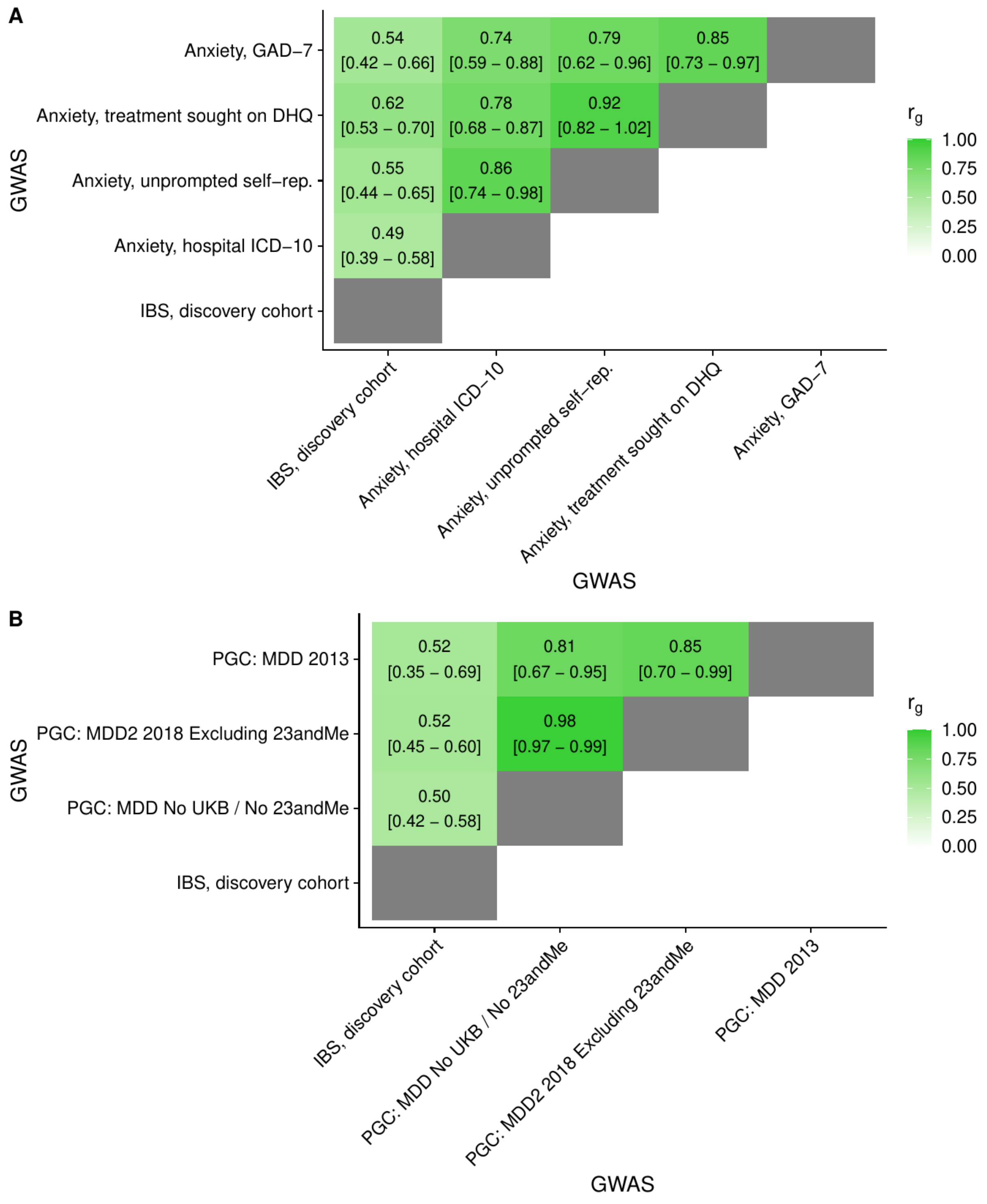

Figure S10: Genetic correlations (r_g) between IBS and various definitions of anxiety or major depressive disorder (MDD) **A.** IBS was robustly correlated with anxiety in UK Biobank, independently of whether anxiety cases were defined by a GAD-7 score≥10, by having sought treatment for anxiety, unprompted self-reporting of anxiety/panic attacks upon UK Biobank enrolment, or hospital records data in the form of ICD-10 codes. Controls were required not to have anxiety by any of these definitions. **B.** Genetic correlations between IBS and MDD were consistent across different definitions of MDD. Wray et al. (2018) cases "met standard criteria for MDD, were directly interviewed [...], or had medical record review by an expert diagnostician", and

were supplemented by data employing "typical" case inclusion criteria from other consortia (see publication). We observed no significant difference depending on whether UK Biobank cases are or are not added to the above (PGC: MDD2 2018 Excluding 23andMe, PGC MDD No UKB / No 23andMe). In data from the Major Depressive Disorder Working Group of the Psychiatric GWAS consortium (2013, PGC: MDD 2013), cases were required to have "diagnosis of DSM-IV lifetime MDD established using structured diagnostic instruments from direct interviews by trained interviewers, or clinician-administered DSM-IV checklists". All MDD data are available from the Psychiatric Genomics Consortium (PGC) website under the exact names provided here.

There are several specific issues that require clarification or discussion.

1. It is unclear why the authors chose to combine across all of the subgroups of IBS for the GWAS analyses rather than examine the subtypes with greatest diagnostic certainty or severity. The exceedingly high prevalence rate of IBS of 14.5% was surprising and likely indicates a mixture of truly heritable cases and those that may be time limited symptom elevations.

The rationale for combining across all of the subgroups is described below. However, we have also now run two additional analyses on subgroups with greater diagnostic certainty (we have phrased this as 'higher-specificity') and greater severity. For the higher-specificity IBS diagnosis GWAS, cases had to meet two or more of our case definitions in UK Biobank; and for the severe IBS GWAS, cases required severe symptoms, defined as IBS-SSS>300. Through the higher-specificity analysis, we found two replicated loci already presented in this manuscript (rs150079703 and rs4455799, both found in Table S13, the latter as rs5017929, with which it is in high LD, r^2=0.93). Via the severe IBS GWAS, we found a single locus, rs9947289, which did not replicate in the 23andMe general IBS dataset (which may reflect the fact that the 23andMe cases are not specifically severe IBS). Both the high-specificity and the severe IBS risk profiles are genetically correlated with multiple neuroaffective phenotypes, echoing the result from the overall discovery cohort. We have amended the main text and Fig. S11 accordingly:

> "We also ran higher-specificity (IBS cases meeting at least two of the four UK Biobank case definitions, 11,201 cases and 293,220 controls) and high severity (IBS-SSS>300, 4,296 cases and 72,356 controls) analyses in UK Biobank. The former produced no novel associations. The latter, while being more heritable (liability-scale h2=0.42, SE=0.05, Cochran's Q: 51.7, $p=6.31\times10^{-13}$ compared to discovery cohort IBS), produced one association (rs9947289, $p=2.80\times10^{-8}$) that did not replicate (p=0.57 in the 23andMe data, Table S13). Both of these phenotypes recapitulated the same genetic correlation with neuroaffective traits as found in the discovery cohort (Fig. S11)."

A preview of Fig. S11 is shown below:

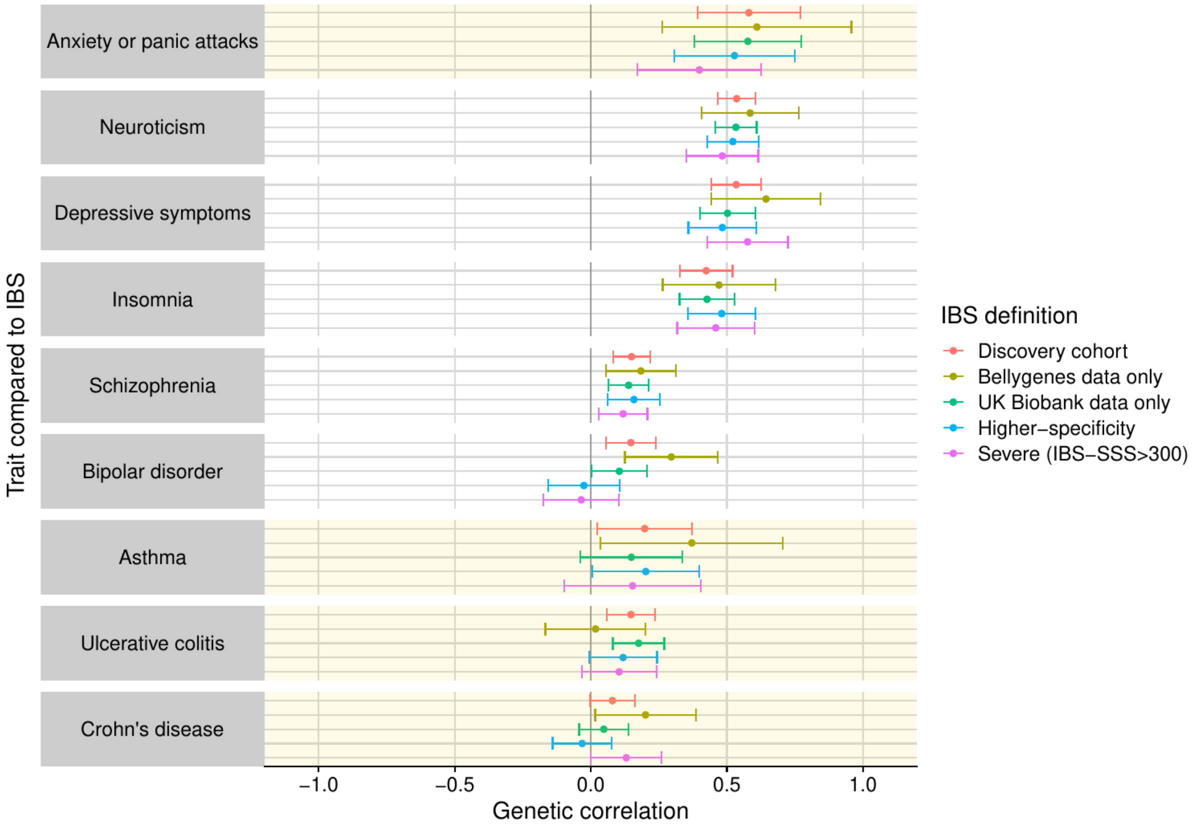

Figure S11: Genetic correlations and 95% confidence intervals between various definitions of IBS and other traits. The selection of traits is identical to that in Figure 3, i.e. traits from peer-reviewed publications in LD Hub most genetically correlated with IBS, supplemented by traits selected for their clinical relevance (yellow). In the higher-specificity analysis, we have restricted cases to those meeting at least two of the four UK Biobank case definitions (Methods). The analysis of severe IBS was limited to DHQ respondents, with cases having an IBS-SSS>300. Across these definitions of IBS, the pattern of genetic correlation with neuroaffective traits remains consistent.

For our discovery cohort we chose to combine across all of the case definition subgroups for a number of key reasons:

1. In order to maximise our sample size – reported by DeBoever et al. (2020) as the most powerful strategy for detecting susceptibility loci associated with common traits and diseases.
2. Inclusion of cases identified through our DHQ by the gold standard (at the time our study was designed) Rome III criteria for IBS allowed identification of 16,009 new cases compared to those with an existing diagnosis in UK Biobank. We believe it was the substantial increase in sample size and power that underpinned the success of our study compared to previously published reports of IBS genetics. Of note epidemiological studies of IBS have indicated a global prevalence of 11% (Lovell and Ford 2012) – with estimates of UK prevalence ranging from 6 to 21% depending on definition (Canavan, West, and Card 2014). Hence the prevalence of IBS that we observed in UK Biobank is in keeping with the published literature.

3. Use of the broad, combined definition also allowed generalizability between the UK Biobank, Bellygenes and 23andMe cohorts. Data relating to diagnostic certainty and symptom severity were not consistently available for the latter two cohorts.

This approach of combining across all diagnostic definitions was validated by our finding of high genetic correlation between them:

> "To maximise sample size, cases from the four UK Biobank groups were pooled (n=40,548). This approach was supported by demonstrating high genetic correlations between them using Linkage Disequilibrium Score Regression (LDSC)[48] following a separate genome-wide association study (GWAS) on each (min. pairwise r_g=0.70, SE=0.06, Fig. S11) and by previous literature on the consistency of genetic results obtained from different diagnostic definitions in UK Biobank.[16]"

2. It was difficult to understand the samples and phenotypes for the IBS as well as psychological symptoms in the 3 samples, and how subsets were combined in the analyses. The Methods could include a more clear description so that the reader does not have to search the supplementary materials for this broad overview.

We thank the reviewer for highlighting this lack of clarity and have adjusted the text in the methods section accordingly to provide an introductory 'broad overview:

> "Our discovery cohort combined cases of IBS identified in the UK Biobank with cases from the Bellygenes initiative. Replication was sought in an independent panel from 23andMe. Cases ascertained in UK Biobank met at least one of the following four conditions (the DHQ, or digestive health questionnaire, is viewable online - UK Biobank resource 595):"

To provide additional information on the ascertainment of IBS subtypes and anxiety and depression cases, we have added the following text to the methods section:

> "Analyses of IBS subtypes were conducted solely using UK Biobank DHQ data based on standard definitions of IBS-C, IBS-D, IBS-M and IBS-U according to the frequency of hard or lumpy stools vs loose, mushy or watery stools. Functional constipation and functional diarrhoea cases were identified similarly, and with the same exclusions per IBS cases, but (in contrast to the Rome III definition of IBS) needed to have responded "Never" when asked about the frequency of abdominal pain in the last 3 months. Likewise, analyses of IBS severity (using the IBS-SSS) and associated somatic symptoms (using PHQ-12) were restricted to DHQ respondents. Anxiety and depression were identified among UK Biobank participants based on previously surveyed responses to GAD-7 anxiety and PHQ-9 depression questionnaires, self-report of diagnosis with depression or anxiety/panic attack, diagnostic codes for major depression and phobic or generalized anxiety disorder in electronic healthcare records, or reporting of treatment being sought or offered for these conditions in our DHQ. See Supplementary Text for details."

3. The diagnoses of IBS were quite different in the three samples, ranging from medical records in the Bellygenes cohort to self-reported questionnaires with dome of the Rome criteria I the UK Biobank to a few questions in 23 and me. How confident are the authors that they are really tapping the same disorder using these diverse methods?

IBS is a heterogeneous disorder and given the subjective nature of the symptom-based diagnostic criteria the extent to which any two people with IBS have the "same disorder" is debatable. Our strategy for identifying cases was to take a deliberately broad and inclusive approach that relied on real-world diagnostic criteria for IBS while also maximizing sample size (key to the success of a genetic study of this type) and generalizability, rather than, e.g., restricting ourselves to individuals with the same symptomatically more homogenous subtype. As a result we do expect to see (and do see) some average differences in presentation or risk factors across the different case definitions.

However, we do have good evidence that, both phenotypically and genetically, these different case definitions show substantial overlap, and more importantly that the results that we present are robust to the specific method of defining IBS.

By examining cases in the UK Biobank who have taken the digestive health questionnaire and have matched medical records (Figure 1A), we can see that most individuals who had a medical record for IBS on file also answer "Yes" to the question "Have you been diagnosed with IBS?" (79%), and are often detected based on Rome III symptom scores at the time of survey (61%). However, there are also patient subsets within this dataset that are tagged by the different case definition methods: for instance, the largest single group (48%) is individuals who have IBS symptoms on the survey but no previous IBS diagnosis. We know that this group differs in its profile of IBS severity and comorbidities on average from the other cases, though they are more similar to diagnosed IBS than they are to digestively healthy controls (Figure 1B, Table S4). Overall, we believe that these methods pick up a strongly but not perfectly overlapping set of cases with comparable but not identical disease manifestation.

Regardless of the degree of actual overlap in samples, it is clear that these different case definitions produce sets of samples that show a substantial genetic overlap. The genetic correlation, or coheritability, between these different case definitions within UK Biobank ranges from 70-100% (Figure S13), and the genetic correlation between UK Biobank and Bellygenes was also very high (99.8%).

Our findings were also consistent across these different case definitions. The six replicating loci identified in the whole discovery dataset showed consistent evidence of association across case definitions (shown in a new Figure S8), and the genetic correlation with neuroaffective traits was significant and highly consistent across all case definitions (Table S16).

In conclusion, we are confident that these case definitions encompass phenotypically and genetically comparable, if not identical, sets of IBS patients. With the approach taken we are confident that the results we present are not specific to a particular case definition and generalize across different methods of defining IBS.

4. The epidemiologic associations with IBS in the UK Biobank were quite interesting and could provide further insight into potential sources of heterogeneity. Could the subtypes of IBS be further characterized by potential environmental correlates that were described in this report?

We likewise found the epidemiological associations to be interesting. None of these evidently showed higher correlation with any of the established IBS subtypes (IBS-D, IBS-C or IBS-M).

Regarding whether the epidemiological characteristics of the patients could be used to define novel, distinct (and possibly genetically differentiated) subtypes of IBS, we agree that this would be a potentially valuable use of the UK Biobank digestive health questionnaire data, but we feel that it is beyond the scope of the current paper. There are multiple other UK Biobank datasets that could also be used in such an analysis (such as the food frequency questionnaire and serological screens for pathogens), and we hope that by making the DHQ available to the community such larger integrative analyses of the genetic, epidemiological and environmental basis of digestive health will be made possible in the future.

5. The authors should discuss how the 50% response rate to the IBS questionnaire in the UK Biobank may have led to bias in the findings.

We agree that response bias, both in terms of response rate to the DHQ and in terms of recruitment biases to UK Biobank itself, could result in the generation of biased or non-representative findings. Had we not controlled for response bias in our analyses, we would have seen false positives due to genetic or environmental predictors of survey response (rather than IBS) - essentially confusing the effects of survey response and of IBS itself.

We use data for both responders and non-responders in our analyses (where we have data on both), and correct for differences using a stratified meta-analysis approach. We believe that this will minimise the effects of bias, and in general we do not see strong heterogeneity of effect in the association between IBS and genetics for those that did and did not fill out the survey (for analyses where we have data on both).

While this approach controls for bias, there is also the broader question of how the drop-out at each stage (recruitment to UK Biobank, followed by responding to the DHQ) will create a non-representative sample set (previously described in the “Population characteristics” field of the Nature Research Reporting Summary form). As we allude to in the text, further studies will be required to test how well these results generalize beyond our dataset to other (younger, more ethnically diverse) datasets.

To address this issue we have added the following section ("Controlling for response bias") to the supplementary material discussing response bias and how we controlled for it:

> "There are systematic differences between respondents and non-respondents. Respondents have lower rates of IBS as measured via hospital ICD-10 codes (1.16% vs 1.40% among 171,061 respondents and 317,234 non-respondents, respectively), but not unprompted self-reporting (2.85% vs 2.30%). They also have lower rates of neuroaffective conditions based on hospital ICD-10 codes (schizophrenia: 0.04% vs 0.21%, depression: 1.77% vs 3.40%) and unprompted self-reporting (schizophrenia: 0.05% vs 0.16%, depression: 5.83% vs 6.20%). Respondents also had a lower mean age than non-respondents (64.8 vs 65.8 years when the DHQ data were collected), and were more often female (56.7% vs 52.9%). Response rates also varied by ethnicity, e.g. 15.8% (1205 of 7645) among participants who report a Black or Black British background compared to 36.0% (165243 of 459256) among participants reporting a White ethnic background. We show that responder effects can also cause artifactual differences in genetic signals across analyses if not controlled for (Fig. S14). In our genetic association tests, we therefore analyzed DHQ respondents and non-respondents separately, and then meta-analyze the results to eliminate the confounding effect of DHQ response on IBS risk. In non-genetic analysis, e.g. between IBS and clinical risk factors, we control for DHQ response status by adding it as a covariate to our logistic regression models, along with factors such as age and gender."

References

Bonfiglio, Ferdinando, Tenghao Zheng, Koldo Garcia-Etxebarria, Fatemeh Hadizadeh, Luis Bujanda, Francesca Bresso, Lars Agreus, et al. 2018. “Female-Specific Association Between Variants on Chromosome 9 and Self-Reported Diagnosis of Irritable Bowel Syndrome.” *Gastroenterology* 155 (1): 168–79.

Canavan, Caroline, Joe West, and Timothy Card. 2014. “The Epidemiology of Irritable Bowel Syndrome.” *Clinical Epidemiology* 6 (February): 71–80.

DeBoever, Christopher, Yosuke Tanigawa, Matthew Aguirre, Greg McInnes, Adam Lavertu, and Manuel A. Rivas. 2020. “Assessing Digital Phenotyping to Enhance Genetic Studies of Human Diseases.” *American Journal of Human Genetics* 106 (5): 611–22.

Lovell, Rebecca M., and Alexander C. Ford. 2012. “Global Prevalence of and Risk Factors for Irritable Bowel Syndrome: A Meta-Analysis.” *Clinical Gastroenterology and Hepatology: The Official Clinical Practice Journal of the American Gastroenterological Association* 10 (7): 712–21.e4.

Major Depressive Disorder Working Group of the Psychiatric GWAS Consortium, Stephan Ripke, Naomi R. Wray, Cathryn M. Lewis, Steven P. Hamilton, Myrna M. Weissman, Gerome Breen, et al. 2013. “A Mega-Analysis of Genome-Wide Association Studies for Major Depressive Disorder.” *Molecular Psychiatry* 18 (4): 497–511.

Wray, Naomi R., Stephan Ripke, Manuel Mattheisen, Maciej Trzaskowski, Enda M. Byrne, Abdel Abdellaoui, Mark J. Adams, et al. 2018. “Genome-Wide Association Analyses Identify 44 Risk Variants and Refine the Genetic Architecture of Major Depression.” *Nature Genetics* 50 (5): 668–81.

Decision Letter, first revision:

Our ref: NG-A56757R

28th May 2021

Dear Miles,

Your revised manuscript "Genome-wide analysis of 53,400 people with irritable bowel syndrome highlights shared pathways with neuroaffective traits" (NG-A56757R) has been seen by the original referees. As you will from their comments below, they are broadly satisfied with the changes made in response to their previous comments, and therefore we will be happy in principle to publish your study in Nature Genetics as an Article pending final revisions to satisfy Reviewer #2's remaining point and to comply with our editorial and formatting guidelines.

We are now performing detailed checks on your paper and will send you a checklist detailing our editorial and formatting requirements in about a week. Please do not upload the final materials and make any revisions until you receive this additional information from us.

Thank you again for your interest in Nature Genetics Please do not hesitate to contact me if you have any questions.

Sincerely,
Kyle

Kyle Vogan, PhD
Senior Editor
Nature Genetics
https://orcid.org/0000-0001-9565-9665

Reviewer #1 (Remarks to the Author):

Thanks to the authors for their thoughtful responses and changes to the manuscript. I agree it is a shame more could not be done in terms of obtaining 23andMe summary data. I have no further comments.

Reviewer #2 (Remarks to the Author):

The authors have carefully and thoughtfully addressed the issues raised by my review and that of the other reviewers and the paper has improved substantially.

There is one important point regarding terminology that should be addressed.

Since the conclusion is shared etiology between the mood and anxiety symptoms, the use of the term "neuroaffective" is misleading because it implies that other neurologic indices were included when the phenotypes including mood and anxiety symptoms. These are current symptoms scales rather than trait measures so it would be more accurate to refer to them as "Mood/Anxiety Symptoms" rather than adopting in a new term that is no commonly used to refer to these widely used measures, and may be misleading.

Author Rebuttal, first revision:

Response to referees

Genome-wide analysis of 53,400 people with irritable bowel syndrome highlights shared genetic pathways with mood and anxiety disorders

Reviewer #1:

Remarks to the Author:

Thanks to the authors for their thoughtful responses and changes to the manuscript. I agree it is a shame more could not be done in terms of obtaining 23andMe summary data. I have no further comments.

We thank the reviewer for their feedback on the paper. We would also have liked to include more 23andMe data in this paper still, and hope these data will be released in the future.

Reviewer #2:

Remarks to the Author:

The authors have carefully and thoughtfully addressed the issues raised by my review and that of the other reviewers and the paper has improved substantially.

We thank the reviewer for their detailed and spirited review.

There is one important point regarding terminology that should be addressed.
Since the conclusion is shared etiology between the mood and anxiety symptoms, the use of the term "neuroaffective" is misleading because it implies that other neurologic indices were included when the phenotypes including mood and anxiety symptoms. These are current symptoms scales rather than trait measures so it would be more accurate to refer to them as "Mood/Anxiety Symptoms" rather than adopting in a new term that is no commonly used to refer to these widely used measures, and may be misleading.

We thank the reviewer for helping to make the paper more accurate and also more accessible by replacing the word “neuroaffective”. We spent a long time during the initial paper writing trying to find the right word to describe exactly what we meant, and settled on “neuroaffective”, but it is clear in retrospect that this caused more confusion than understanding. The terms “mood and anxiety”, while they do not capture everything we are trying to describe explicitly, do capture most of it, and are clearly more recognizable.

We prefer the use of “disorders” over “symptoms”, given that the traits IBS was compared to were clinical syndromes rather than individual symptoms (as per the most recent version of Supplementary Fig. 10, which contains various definitions of major depressive disorder and anxiety disorders).

We have replaced the use of “neuroaffective” by “anxiety and mood disorders” (or, in some places, “mental health and personality traits”, when that was more accurate) throughout the manuscript.

Final Decision Letter:

In reply please quote: NG-A56757R1 Jostins

8th September 2021

Dear Luke,

I am delighted to say that your manuscript "Genome-wide analysis of 53,400 people with irritable bowel syndrome highlights shared genetic pathways with mood and anxiety disorders" has been accepted for publication in an upcoming issue of Nature Genetics.

Prior to setting your manuscript, we may make minor changes to enhance the lucidity of the text and with reference to our house style. We therefore ask that you examine the proofs most carefully to ensure that we have not inadvertently altered the sense of your text in any way.

Once your manuscript is typeset and you have completed the appropriate grant of rights, you will receive a link to your electronic proof via email with a request to make any corrections within 48 hours. If, when you receive your proof, you cannot meet this deadline, please inform us at rjsproduction@springernature.com immediately.

Your paper will be published online after we receive your corrections and will appear in print in the next available issue. You can find out your date of online publication by contacting the Nature Press Office (press@nature.com) after sending your e-proof corrections. Now is the time to inform your Public Relations or Press Office about your paper, as they might be interested in promoting its publication. This will allow them time to prepare an accurate and satisfactory press release. Include your manuscript tracking number (NG-A56757R1) and the name of the journal, which they will need when they contact our Press Office.

Before your paper is published online, we will be distributing a press release to news organizations worldwide, which may very well include details of your work. We are happy for your institution or funding agency to prepare its own press release, but it must mention the embargo date and Nature Genetics. Our Press Office may contact you closer to the time of publication, but if you or your Press Office have any enquiries in the meantime, please contact press@nature.com.

Acceptance is conditional on the data in the manuscript not being published elsewhere, or announced in the print or electronic media, until the embargo/publication date. These restrictions are not intended to deter you from presenting your data at academic meetings and conferences, but any enquiries from the media about papers not yet scheduled for publication should be referred to us.

Please note that Nature Genetics is a Transformative Journal (TJ). Authors may publish their research with us through the traditional subscription access route or make their paper immediately open access through payment of an article-processing charge (APC). Authors will not be required to make a final decision about access to their article until it has been accepted. Find out more

about Transformative Journals

<B>Authors may need to take specific actions to achieve compliance with funder and institutional open access mandates. For submissions from January 2021, if your research is supported by a funder that requires immediate open access (e.g. according to Plan S principles) then you should select the gold OA route, and we will direct you to the compliant route where possible. For authors selecting the subscription publication route, our standard licensing terms will need to be accepted, including our self-archiving policies. Those standard licensing terms will supersede any other terms that the author or any third party may assert apply to any version of the manuscript.

Please note that Nature Research offers an immediate open access option only for papers that were first submitted after 1 January 2021.

You will not receive your proofs until the publishing agreement has been received through our system.

If you have any questions about our publishing options, costs, Open Access requirements, or our legal forms, please contact ASJournals@springernature.com

If you have posted a preprint on any preprint server, please ensure that the preprint details are updated with a publication reference, including the DOI and a URL to the published version of the article on the journal website.

To assist our authors in disseminating their research to the broader community, our SharedIt initiative provides you with a unique shareable link that will allow anyone (with or without a subscription) to read the published article. Recipients of the link with a subscription will also be able to download and print the PDF.

As soon as your article is published, you will receive an automated email with your shareable link.

You can now use a single sign-on for all your accounts, view the status of all your manuscript submissions and reviews, access usage statistics for your published articles and download a record of your refereeing activity for the Nature journals.

An online order form for reprints of your paper is available at https://www.nature.com/reprints/author-reprints.html. Please let your coauthors and your institutions' public affairs office know that they are also welcome to order reprints by this method.

If you have not already done so, we invite you to upload the step-by-step protocols used in this manuscript to the Protocols Exchange, part of our on-line web resource, natureprotocols.com. If you complete the upload by the time you receive your manuscript proofs, we can insert links in your article that lead directly to the protocol details. Your protocol will be made freely available upon publication of your paper. By participating in natureprotocols.com, you are enabling researchers to more readily

reproduce or adapt the methodology you use. Natureprotocols.com is fully searchable, providing your protocols and paper with increased utility and visibility. Please submit your protocol to https://protocolexchange.researchsquare.com/. After entering your nature.com username and password you will need to enter your manuscript number (NG-A56757R1). Further information can be found at https://www.nature.com/nprot/.

Sincerely,
Kyle

Kyle Vogan, PhD
Senior Editor
Nature Genetics
https://orcid.org/0000-0001-9565-9665
